# Understanding the Role of NHC Ditopic Ligand Substituents in the Molecular Diversity and Emissive Properties of Silver Complexes

**DOI:** 10.1021/acs.inorgchem.4c02940

**Published:** 2024-10-30

**Authors:** Irati Barriendos, Olga Crespo, M. Concepción Gimeno

**Affiliations:** Departamento de Química Inorgánica, Instituto de Síntesis Química y Catálisis Homogénea (ISQCH), Universidad de Zaragoza-CSIC, E-50009 Zaragoza, Spain

## Abstract

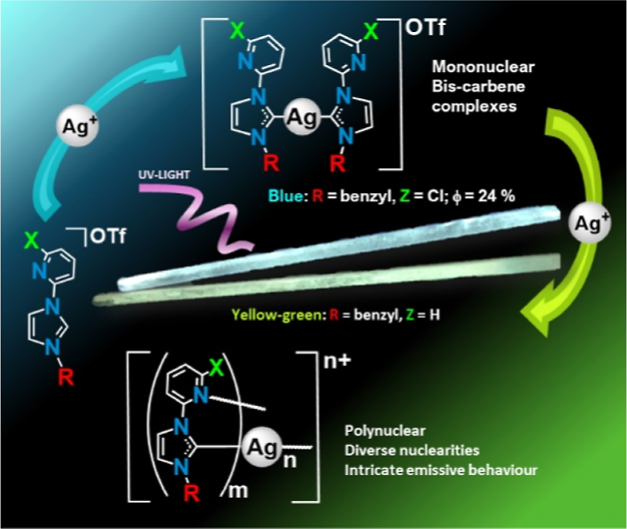

Silver bis(carbene) complexes featuring ditopic N-heterocyclic carbene (NHC) ligands have been synthesized which enable the assembly of supramolecular architectures via reaction with silver triflate. Through a systematic exploration of crystal structures and emissive properties, this study investigates the impact of the substituents on the NHC ditopic ligands [R-Im-2-Z-py], where R = Me, benzyl (Bz), or 2-naphthylmethyl (NaphCH_2_), and Z = H or Cl in the structural framework and emissive properties observed in the silver bis(carbene) or polynuclear species. Remarkably diverse structural motifs emerge in both of them, predominantly influenced by the choice of R wingtip and second by the Z substituent. Also the emissive quantum yield of the complexes is mostly governed by the selection of the R wingtip and further modulated by the Z substituent. Several of the mono and polynuclear complexes exhibit complex emissive profiles, including the observation of dual emission phenomena. Particularly noteworthy is the complex [Ag(NHC)]_2_]OTf (R = Bz, Z = Cl), which demonstrates an exceptionally intense single blue emission with a remarkable solid-state quantum yield (Φ) of 24%.

## Abstract 2

Silver mononuclear homoleptic bis(carbene) complexes [Ag(NHC)_2_]^+^, featuring ditopic carbene ligands [NHC = **R**-(Im)-2-ZPy (**R** = Me, benzyl (Bz) or 2-naphthylmethyl (NaphCH_2_); **Z** = H or Cl)], react with silver triflate to afford polynuclear complexes, displaying a vast array of structures. The emissive behavior and crystal arrangements of the mono and polynuclear compounds are analyzed with respect to the influence of the **R** and **Z** substituents. Crystal structure diversity appears to be mostly modulated by changing the **R** substituent. Many of the complexes exhibit complex emissive behavior, including dual emission, being noticeable the intense blue emission (Φ = 24%) shown by compound [Ag(NHC)]_2_]OTf (**R** = Bz, **Z** = Cl) in the solid state.

## Introduction

Silver N-heterocyclic carbene (NHC) complexes serve as pivotal precursors in the synthesis of diverse transition metal NHC species and exhibit intriguing structural diversity. The absence of ancillary ligands completing the silver coordination sphere leads to modifications in structural patterns, reactivity, and optical or biological properties, while also aiding in understanding the role of the carbene ligands in these aspects. Therefore, in this Introduction section, the selected references focus on homoleptic [Ag_*n*_(NHC)_*m*_]^*n*+^ NHC complexes.

The significance of monodentate silver NHC derivatives as transmetalating agents^1−4^ as well as their structural wealth^5^ extends to silver complexes featuring polytopic NHC ligands. Beyond their role as synthetic precursors, these silver NHC compounds hold promise for a diversity of applications due to their catalytic,^6^ biological^7−9^ or emissive^10,11^ properties.

**Figure 1:**
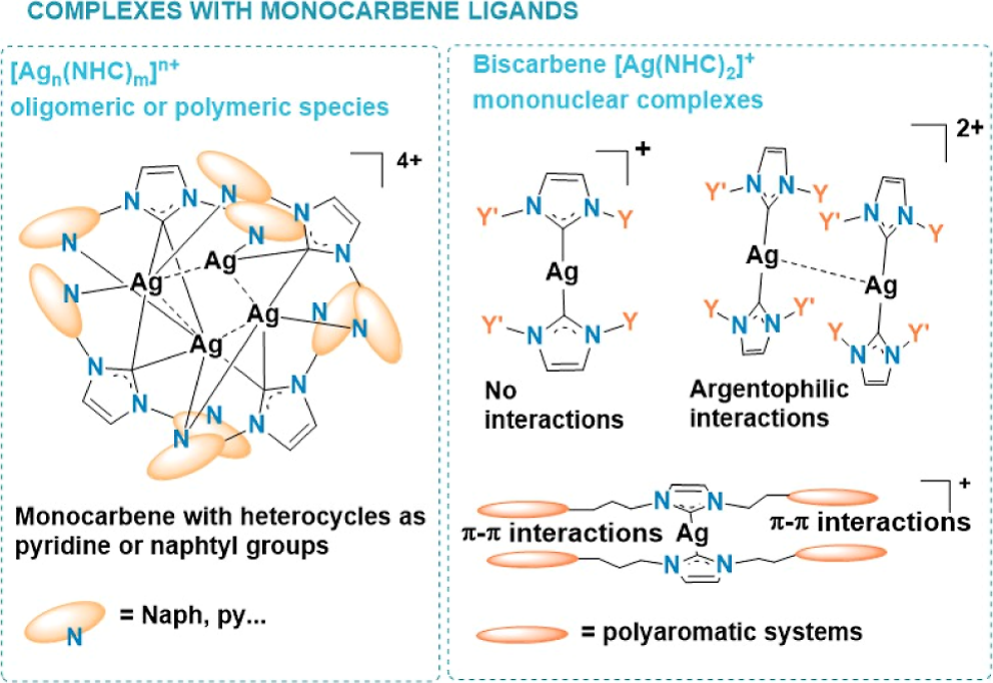
Some structural reported motifs of silver homoleptic complexes with monocarbene NHC ligands.

**Figure 2:**
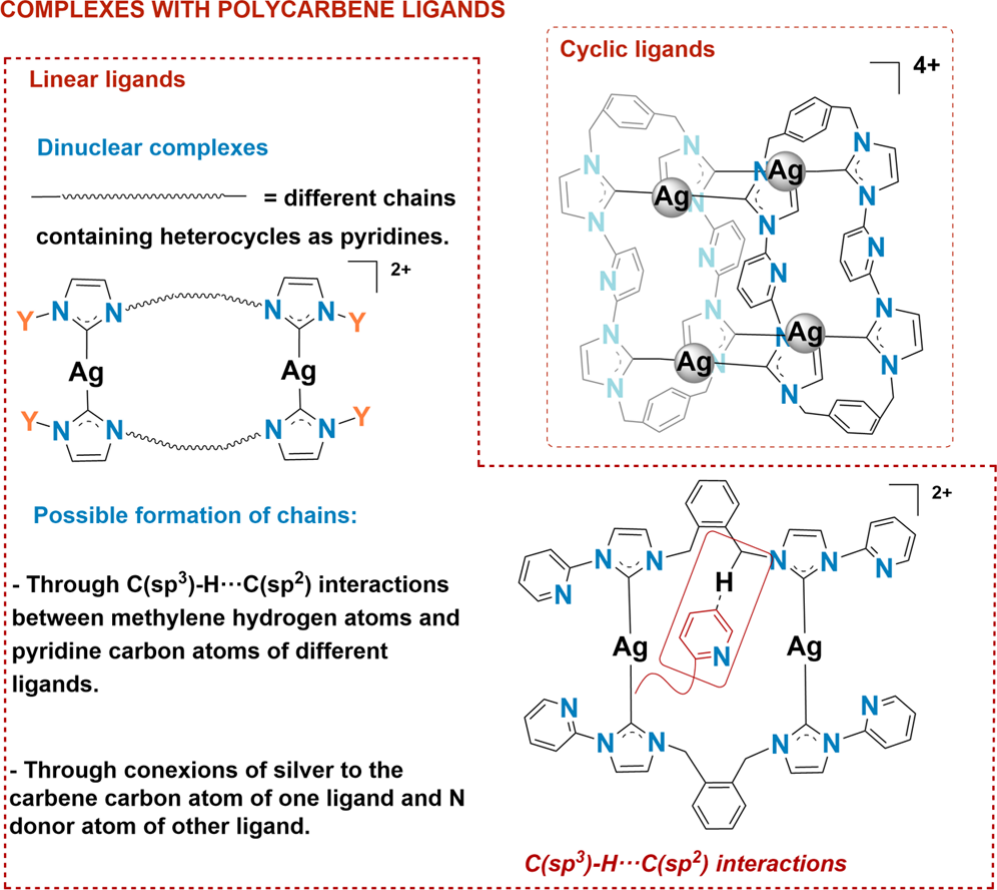
Some structural reported motifs of silver homoleptic complexes with polycarbene NHC ligands.

The analysis of several reviews^12−17^ regarding the synthesis, structural arrangements, and properties of silver NHC complexes reveals a diverse depiction of the different ligand types. While tetra N^C^C^N, tridentate (N^C^N; C^N^C), polycarbene, and various pincer or macrocyclic ligands are often represented, examples featuring N^C ditopic carbenes are relatively scarce. Figures 1 and 2 illustrate part of the wide variety of structural motifs reported with mono or poly carbene ligands. Intricate discrete polynuclear units or chains are built through covalent Ag–N(heterocycle) and Ag–C(carbene) bonds assisted in many cases by weak interactions of different nature.

Some examples of silver complexes incorporating these N^C ditopic ligands serve as intermediates in the synthesis of heteroleptic metal complexes with high-demand applications, via reactions with other ancillary ligands.^18,19^

These homoleptic [Ag_*n*_(NHC)_*m*_]^*n*+^ complexes exhibit a general structural trend concerning the anion. Despite its coordination capability, the anion is seldom bonded to silver, playing a lesser role in the resulting final silver environment, as illustrated in nitrate^20−23^ perchlorate^24,25^ or trifluoromethanesulfonate (OTf)^26−29^ complexes.

The number of studies aimed at elucidating the extensive structural diversity^5^ of these compounds and understanding its influence on their emissive behavior^30^ remains limited.

**Scheme 1:**
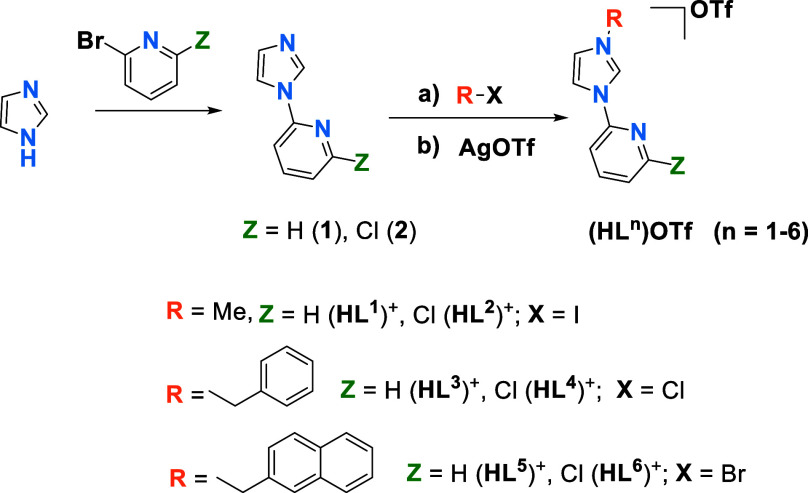
General Procedure for the Synthesis of the Imidazolium **(HL**^**n**^**)OTf** Salts

Recently several reviews have dealt with the emissive properties of coinage metal carbene complexes.^31−33^ However, luminescent reports for these homoleptic ditopic silver NHC complexes rarely provide quantum yields or lifetime data. The absence of this critical information hampers the ability to predict their suitability for various applications, such as OLEDs or sensors. Given the aforementioned gaps, our aim is to investigate the molecular diversity resulting from the use of homoleptic silver complexes [Ag(NHC)_2_]^+^ as building blocks for polynuclear arrangements and the consequences of the different structures in the emissive properties of these silver complexes. Herein, we report on the synthesis of mononuclear bis(carbene) species and their subsequent reaction with silver triflate to produce polynuclear silver complexes with a diverse array of structural frameworks which affect to the emissive properties. Scheme 1 illustrates the selected NHC ligands, showcasing six different OTf^–^ salts of ditopic carbenes employed in this study. Two structural modifications resulting from the variation of the substituent at the pyridine ring (Z = H or Cl) and the wingtip of the carbene ligand [R = Me, benzyl (Bz), or 2-naphthylmethyl (NaphCH_2_)] have been introduced.

Our purpose is to analyze the impact of these carbene skeleton modifications on the crystal structures and emissive behavior of the silver complexes. To achieve this, steady-state, time-resolved fluorescence, and quantum yield measurements of their respective emissions were conducted.

## Discussion

### Synthesis and Characterization of Mononuclear Silver Complexes

The imidazolium salts (HL^1^)OTf-(HL^6^)OTf have been synthesized through literature procedures or modification of reported methods according to Scheme 1 (some of them had been previously reported as PF_6_^–^ salts, see Experimental Section and Supporting Information).

The general procedure consists of the reaction of imidazole with 2-bromopyridine or 2-chloro-6-bromopyridine to afford the intermediate species HIm-Py (**1**) or HIm-2-ClPy (**2**). Reaction of these intermediates with iodomethane or the corresponding halo-methylaryl compound leads to the [R-HIm-2-ZPy]X salt (X = Cl, Br, I). Anion exchange to afford **(HL**^**n**^**)OTf** is accomplished through treatment with AgOTf.

**Scheme 2:**
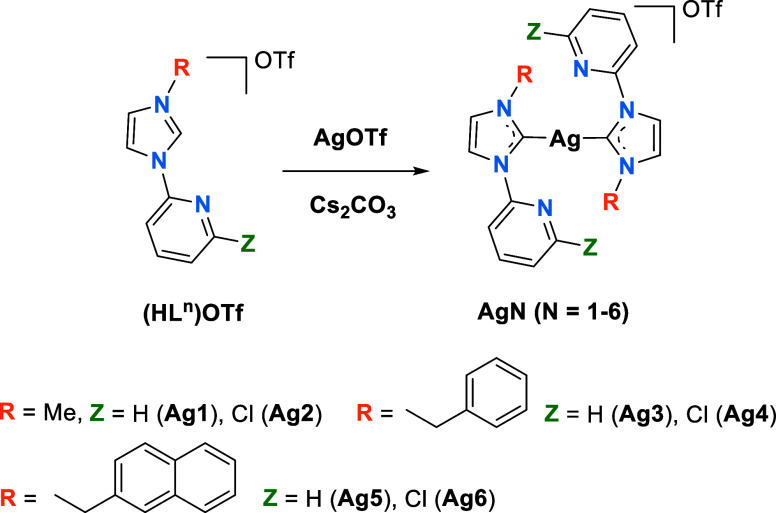
Synthesis of the Bis(carbene) Complexes **Ag1–Ag6**

The bis(carbene) complexes **Ag1–Ag6** are obtained as a result of the reaction of the imidazolium salts **(HL**^**n**^**)OTf** with AgOTf and Cs_2_CO_3_ in dichloromethane (Scheme 2). For synthesis and characterization details (see Experimental Section and Supporting Information).

The deprotonation of the imidazolium fragment is confirmed by the absence of the acidic NHC proton, typically observed at approximately 10 ppm in **(HL**^**n**^**)OTf** salts. The two hydrogen atoms of the carbene skeleton appear around 8 ppm, the signal corresponding to the hydrogen atoms of the methyl substituent in **Ag1** and **Ag2** is observed near 4 ppm and that corresponding to the methylene fragment of the benzyl or 2-naphthylmethyl substituent in **Ag3**–**Ag6** is found close to 5 ppm.

The signal at approximately 57 ppm in the ^13^C{^1^H} NMR spectra of **Ag3–Ag6** is attributed to the CH_2_ fragment of the benzyl or 2-naphthylmethyl **R** group, whereas at 40 ppm appear those corresponding to the methyl **R** substituent in the spectra of **Ag1** and **Ag2**. The signal related to the carbene carbon atom is only observed for **Ag5**, at 180 ppm.

Crystal structures of the mononuclear homoleptic species **Ag3** and **Ag4** have been elucidated by X-ray crystallography. Different arrangements have been found, depending on the presence or not of the chloride substituent in the pyridine ring while maintaining the wingtip ligand (Bz) on the carbene. A remarkable difference is the formation of dimers through argentophilic interactions in **Ag3**, not observed in **Ag4**. More in-depth information regarding to these structures can be found in the sketch below.

For compound **Ag3** (Figure 4, left) the silver center is coordinated to the carbene carbon atoms of two ligands and displays an almost linear geometry with Ag···N distances of 3.027(7) and 3.32950(11) Å to the nitrogen atoms of the pyridine rings.

**Figure 3:**
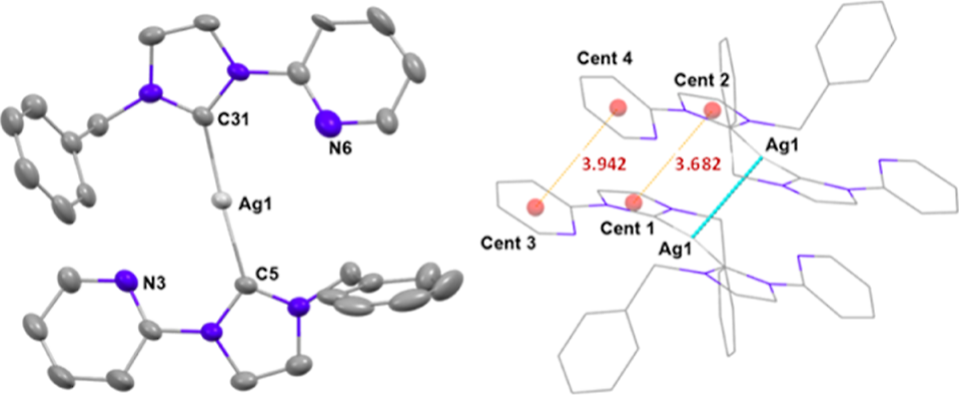
Diagrams of the cation of **Ag3**. Hydrogen atoms have been omitted for clarity. Left: Ortep diagram of the molecule. Ellipsoids represent 50% probability level. Right: Dimer formation showing distances between centroids (Cent) of different rings.

Dimers are formed through Ag···Ag intermolecular interactions^34^ of 3.2950(11) Å (Figure 3, right) in which the distance between centroids of pyridine rings belonging to different molecules is 3.942 Å and that between centroids (Cent) of the carbene rings of different molecules is 3.682 Å, within in the range of those reported for π–π interactions.^5^ These pairs of molecules are arranged in the lattice at distances longer than 11 Å.

**Figure 4:**
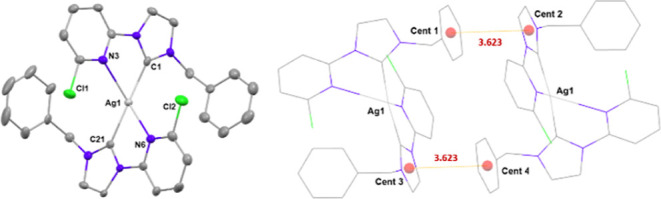
Diagrams of the cation of **Ag4**. Hydrogen atoms have been omitted for clarity. Left: Ortep diagram. Ellipsoids represent 50% probability level. Right: Detail of distances between centroids of carbene and phenyl rings of different ligands.

In complex **Ag4**, the silver center is coordinated to the carbene carbon atoms of two **L**^**2**^ ligands and the Ag–N distances of 2.691 and 2.716 Å are shorter to those found in **Ag3**, leading to a greater distortion from the ideal lineal geometry (Figure 4). Chloride atoms of both ligands point to the silver center at distances of 3.995(2) and 4.001(3) Å.

No intermolecular Ag···Ag interactions are present, but distances of 3.623 Å between centroids of carbene and phenyl rings of different ligands aggregate molecules in pairs through π···π interactions, in which the distance between silver atoms is 7.885 Å (Figure 4, right). The shortest distance between silver atoms in different pairs of molecules is 8.680 Å.

### Synthesis and Characterization of Polynuclear Silver Complexes

**Scheme 3:**
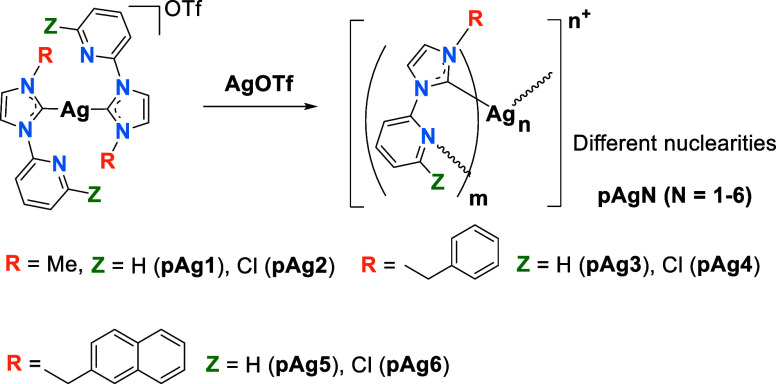
Synthesis of Complexes **pAg1**–**pAg6**

Polynuclear complexes **pAg1–pAg6** have been synthesized by reaction of **Ag1–Ag6** with AgOTf in molar ratio 1:1 (Scheme 3). The ^1^H NMR spectra pattern of each polynuclear complex resembles that of the corresponding silver bis(carbene) precursor compound, but with an increase in signal complexity (see Experimental Section and Supporting Information for characterization details).

The aggregations uncovered from X-ray crystallographic studies can be classified as polymeric chains (**pAg1–pAg3**), dinuclear (**pAg4**, **pAg6**) or trinuclear (**pAg5**), depending on the **R** and **Z** fragments of the **L**^**n**^ ligand. The depictions below provide specific details about these structures. The possible presence of π–π interactions has received careful consideration as they may be associated with excimer formation (see Photophysical Studies section).

#### Polymeric Chains

A remarkable difference between the crystal structure of complexes **pAg1–pAg3** is that, although bridging NHC ligands support the structural chain arrangement, argentophilic interactions connect the silver atoms throughout the chains only in **pAg1** and **pAg2**.

**Figure 5:**
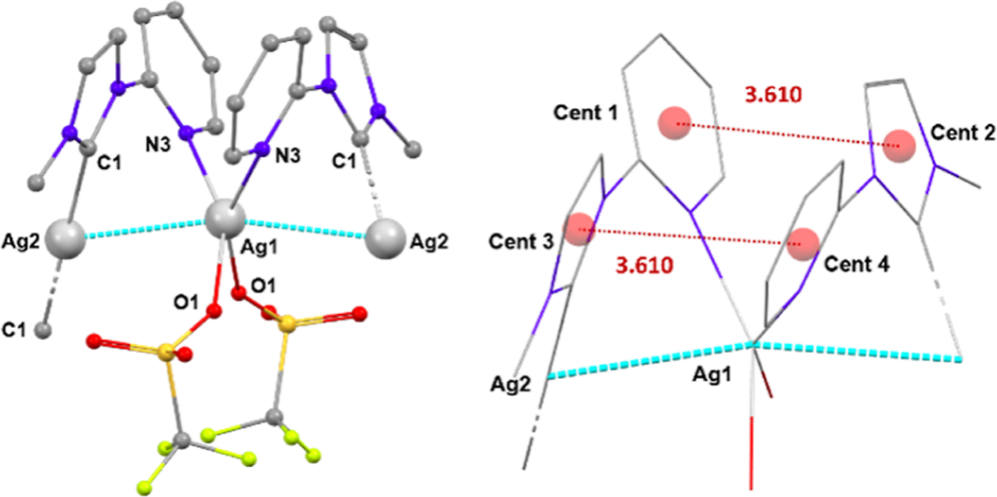
Diagrams corresponding to **pAg1**. Hydrogen atoms have been omitted for clarity. Left: Organization of chains in **pAg1**. Right: Distances between centroids of pyridine and imidazole rings. To ensure clarity, the triflate anions are not included, except for the oxygen atoms that coordinate to silver.

Complex **pAg1** crystallizes as a linear chain of silver atoms bridged by **L**^**1**^ ligands. The asymmetric unit consists of an aggregate of two silver atoms [Ag(1)···Ag(2) 3.1229(4) Å]. Extension of this asymmetric unit leads to the formation of chains in which Ag(1) (Figure 5), is coordinated to two nitrogen atoms of pyridine fragments of different **L**^**1**^ ligands and two oxygen atoms of different triflate anions in a very distorted tetrahedral geometry. The silver atom Ag(2) is bonded to two carbene carbon atoms of different ligands in a linear environment. Distance between centroids of the pyridine and imidazole rings of different ligands is 3.610 Å.

**Figure 6:**
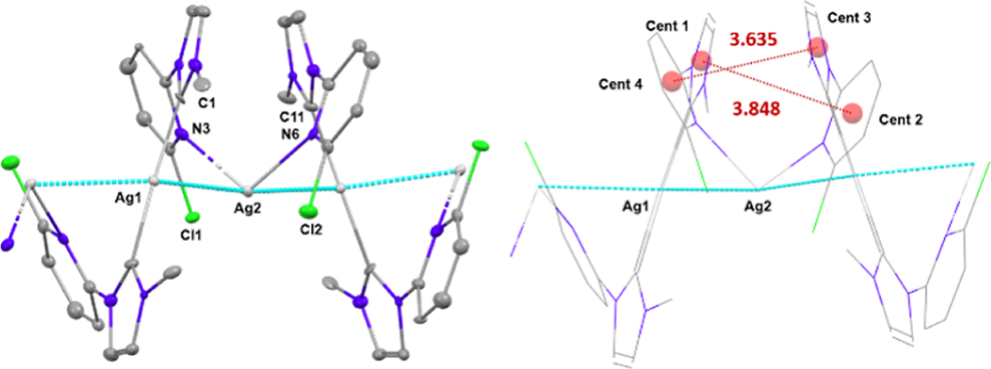
Diagrams corresponding to the cation of **pAg2**. Hydrogen atoms have been omitted for clarity. Left: ORTEP diagram of the chain arrangement in the cation of **pAg2**. Ellipsoids represent 50% probability. Right: Detail of distances between centroids of carbene and pyridine rings.

In the crystal structure of **pAg2** the silver atoms show a zigzag shaped chain pattern with alternated Ag···Ag distances of 2.9166(17) and 2.9800(17) Å (Figure 6). The silver atom Ag(1) is bonded to two carbene carbon atoms of different **L**^**2**^ ligands in a distorted linear environment and Ag(2) is coordinated to two pyridine fragments of different **L**^**2**^ ligands [N6–Ag2–N3 is 107.5(5)°]. The chloride atoms, Cl(1) and Cl(2), point to Ag(2) at distances of 3.078(4) and 3.187(4) Å, respectively, which could indicate a weak bonding interaction. Distances between carbene rings and pyridine rings of different ligands are 3.635 and 3.848 Å.

**Figure 7:**
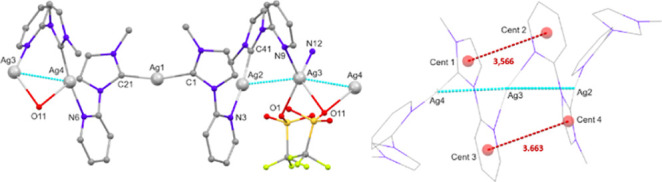
Simplified diagrams depicting chain organization in **pAg3**. The phenyl rings of the benzyl groups, as well as the triflate anions non bonded to the silver atoms and the hydrogen atoms have been omitted for clarity. Left: Molecular diagram showing the atom labeling scheme. Right: Detail of the interactions between centroids of different rings. The triflate anions bonded to **Ag3** have been omitted for clarity.

As explained above, an important difference between the chain structure arrangement in **pAg3**, consisting of silver atoms bridged by **L**^**3**^ ligands, and chain aggregation in **pAg1** or **pAg2**, is that Ag···Ag contacts do not extend all over the chain in **pAg3** (Figure 7).

The asymmetric unit contains four silver atoms. The silver atoms Ag(1), Ag(2) and Ag(4) display distorted linear geometries. Whereas Ag(1) coordinates to two carbene carbon atoms of different ligands, Ag(2) and Ag(4) are bonded to the carbene carbon atom on one ligand and the nitrogen atom of the pyridine unit of different ligands. Also, one oxygen atom of a triflate anion [O(11)] is directed to Ag(4), this oxygen atom is also directed to Ag(3) with Ag(4)–O(11) and Ag(3)–O(11) distances longer than 2.6 Å. In addition to this long interaction, the silver atom Ag(3) is bonded to two nitrogen atoms of pyridine rings of different ligands and the distance to one oxygen atom of another triflate anion [Ag(3)–O(1)] is 2.395(5) Å, shorter than that to O(11), but longer than those found for Ag–O(triflate) in other carbene–silver complexes (2.137 Å).^28^ The silver atom Ag(3) displays short contacts with Ag(4) and Ag(2) of 2.9465(9) and 2.9879(8) Å, respectively, whereas Ag(1) distances to Ag(4) and Ag(2) are longer than 4 Å.

Distances between centroids of the imidazole and pyridine rings of the **L**^**3**^ ligands bonded to Ag(4) and Ag(3) (Figure 7) are 3.566 and 3.683 Å, respectively.

#### Trinuclear Arrangement

**Figure 8:**
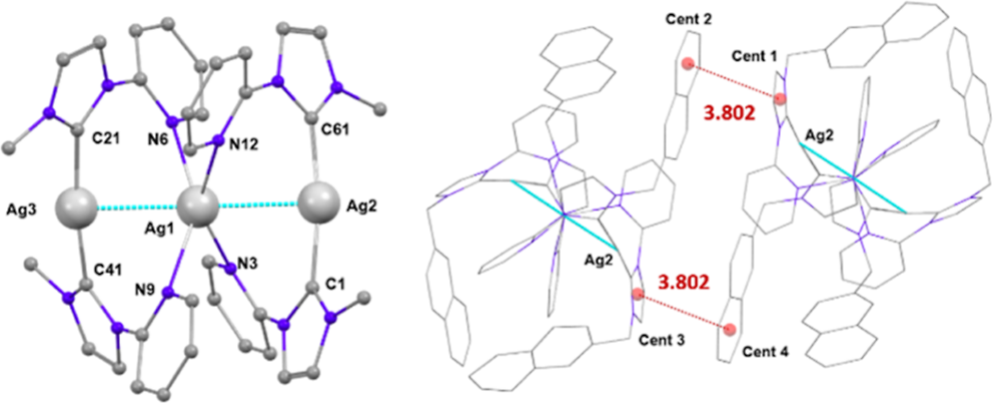
Diagrams of the cation of complex **pAg5**. Hydrogen atoms have been omitted for clarity. Left: Molecular diagram showing the labeling scheme. The naphthyl groups have been omitted for clarity. Right: Interactions between aromatic rings of different molecules.

The crystal structure of **pAg5** comprises three nuclear units. It can be understood as two bis carbene [Ag(**L**^**5**^)_2_]^+^ units bonded through a silver atom. The central silver atom displays tetrahedral distorted geometry, bonded to four nitrogen atoms of four different **L**^**5**^ ligands (Figure 8). The other two silver atoms are bonded to two carbene carbon atoms and exhibit linear distorted environment. The silver atoms are connected through argentophilic interactions of 2.9294(7) and 2.9370(7) Å. The naphthyl groups of the **L**^**5**^ ligands wrap the structure.

Despite the long distances (longer than 8 Å) between silver atoms of different molecules in the lattice, pairs of molecules are connected through interactions between one of the naphthyl rings and the carbene ring of different molecules with distances between centroids of 3.802 Å, as shown in Figure 8.

#### Dinuclear Structures

**Figure 9:**
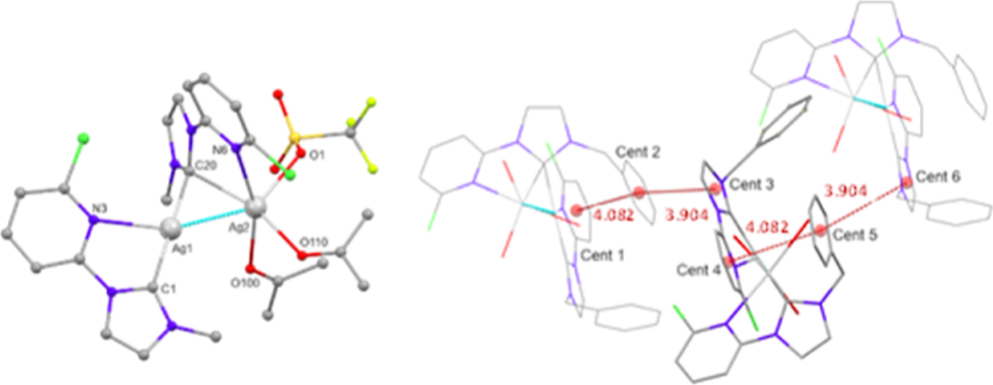
Molecular diagrams of the cation **pAg4-OTf**. Hydrogen atoms have been omitted for clarity. Left: Simplified molecular diagram of the cation of **pAg4-OTf** showing the labeling scheme in which the phenyl substituents of the benzyl groups have been omitted for clarity. Right: Detail showing distances between centroids of different rings in which only the coordinated oxygen atoms of the acetone molecules and triflate anion are shown for clarity.

**Figure 10:**
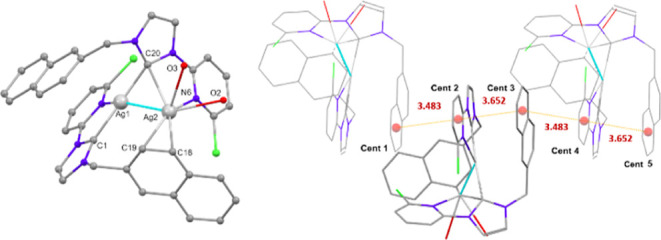
Molecular diagrams of the cation **pAg6-OTf**. Only the oxygen atoms of the triflate anion bonded to silver are shown and the hydrogen atoms have been omitted for clarity. Left: Molecular diagram of **pAg6-OTf** showing the labeling scheme. Right: Detail showing distances between centroids of different rings.

Dinuclear analogous structures are found for **pAg4** and **pAg6** (Figures 9 and 10). Both complexes crystallize as [Ag(**L**^**n**^)]_2_^2+^ dimers, in which one of the silver atoms displays distorted T-shaped geometry and the other silver atom is penta coordinated, with intramolecular Ag···Ag interactions of 2.7353(3) (**pAg4**) or 2.7232(4) Å (**pAg6**) and no intermolecular argentophilic contacts.

In both complexes, the Ag(1) atom is bonded to a nitrogen atom of a pyridine fragment and the carbene carbon atom of the same **L**^**n**^ ligand (*n* = 4, 6). It completes its T-shaped geometry by bonding to the carbene carbon atom of the other **L**^**n**^ ligand, which bridges Ag(1) and Ag(2) in a three center-two electron bond.

In **pAg4** the coordination geometry of Ag(2) is distorted between a square pyramidal and trigonal bipyramidal bonded to the carbene carbon atom and pyridine nitrogen atom of one ligand and to the oxygen atoms of two acetone molecules and a triflate anion.

In **pAg6** the base of the square planar pyramid environment of Ag(2) is completed by the nitrogen atom of the ligand bridging the two silver atoms and two oxygen atoms of the same triflate anion. The apical position is occupied by the 2-naphthylmethyl group of the chelated ligand to Ag1, to which Ag(2) coordinates in a η^2^ mode [C18–Ag2 2.444(4), C19–Ag2 2.504(4) Å].^35^

In complex **pAg4** distances between centroids of the phenyl group of the ligand chelated to Ag2 and the carbene ring of another molecule is 3.904 Å and that between the same phenyl ring and the pyridine ring of the other ligand in the same molecule 4.082 Å (Figure 9).

In **pAg6** intramolecular interactions between one of the naphthyl rings and the pyridine ring of different ligands are observed. These pyridine rings also display intermolecular contacts with naphthyl rings of different molecules (Figure 10).

**Figure 11:**
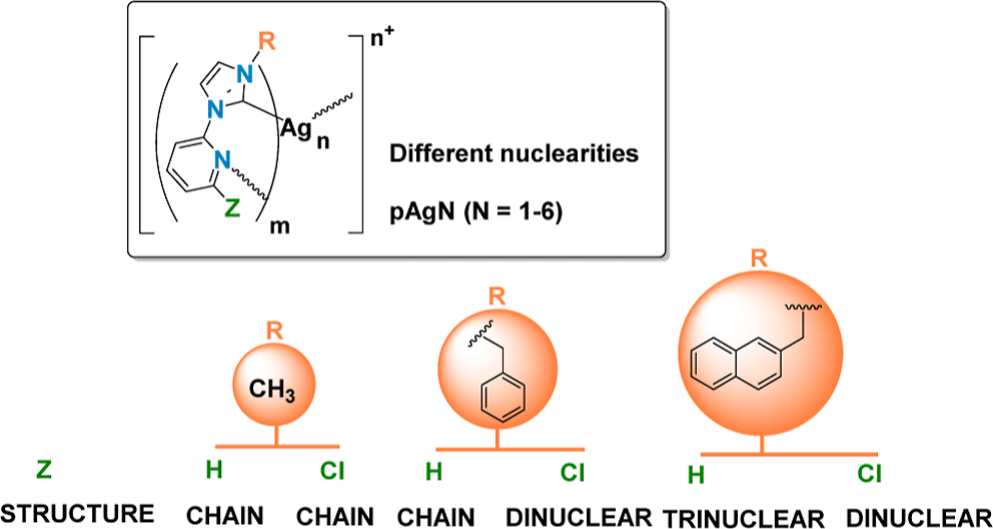
Structural patterns found in the polynuclear complexes **pAgN** (**N** = 1–6).

A global view of these structural data (see Figure 11) may help to rationalize the information and spot trends. It appears evident that modifying the wingtip **R** has a greater impact on the structural organization than changing substituent **Z**. For polynuclear complexes **pAgN**, the larger **R** = 2-naphthylmethyl substituent results in the formation of tri and dinuclear species instead of chains.

As the size of **R** decreases, the influence of the **Z** substituent may modulate the final structure. Thus, for **R** = benzyl the bigger chloride **Z** substituent leads to a dinuclear species and if **Z** = H a chain is obtained. The smallest substituent (**R** = methyl) leads to the formation of chains supported by Ag···Ag interactions, regardless of whether the **Z** substituent is chloride or hydrogen, but such interactions do not spread throughout the entire chain when **R** = benzyl and **Z** = H.

To confirm whether the atomic arrangement of the solid powder, resulting from the chemical reaction, is the same as of the X-ray crystal analysis of **pAg1–pAg6** described above, we performed X-ray powder diffraction studies. The experimental and calculated spectra fit for **pAg1** and **pAg2** (see Supporting Information), proving that atomic distribution in the solid powder is the same as in the crystal. Calculated and experimental spectra for complex **pAg4** do not match which may be explained as the two acetone crystallization molecules are not present in the solid powder. The lack of crystallinity of the samples prevents data discussion for **pAg3** and **pAg5**.

In order to analyze if the polynuclear structures remain in solution DOSY experiments were performed for the selected complexes **Ag5**, **Ag6**, **pAg5** and **pAg6** (see Supporting Information). The calculated diffusion coefficients for the mononuclear compounds **Ag5** and **Ag6** are nearly the same (1.31 × 10^–9^ m^2^/s). The expected decrease is observed for the corresponding polynuclear species **pAg5** and **pAg6** (ca. 1.06 × 10^–9^ m^2^/s). These data are consistent with the persistence of the polynuclear units in solution.

### Photophysical Studies

The emissive properties of both **AgN** and **pAgN** (*N* = 1–6) have been studied in the solid state at room temperature and at 77 K.

As mentioned in the Introduction section, some recent reviews have explored the emissive behavior of group 11 metal carbene complexes.^36−41^ These studies have attributed the emissions to IL transitions (modified or not by the presence of the metal), to the presence of Ag···Ag interactions or to meta-to-ligand charge transfer transitions (MLCT). Most of these studies are limited to the analysis of the complexes in solution and the formation of excimers has been also claimed as the origin of some of the emissions observed.

With this scenario in mind, before proposing the origin of the observed emissions, a detailed description of the photophysical data is given below.

#### Data Description

**Figure 12:**
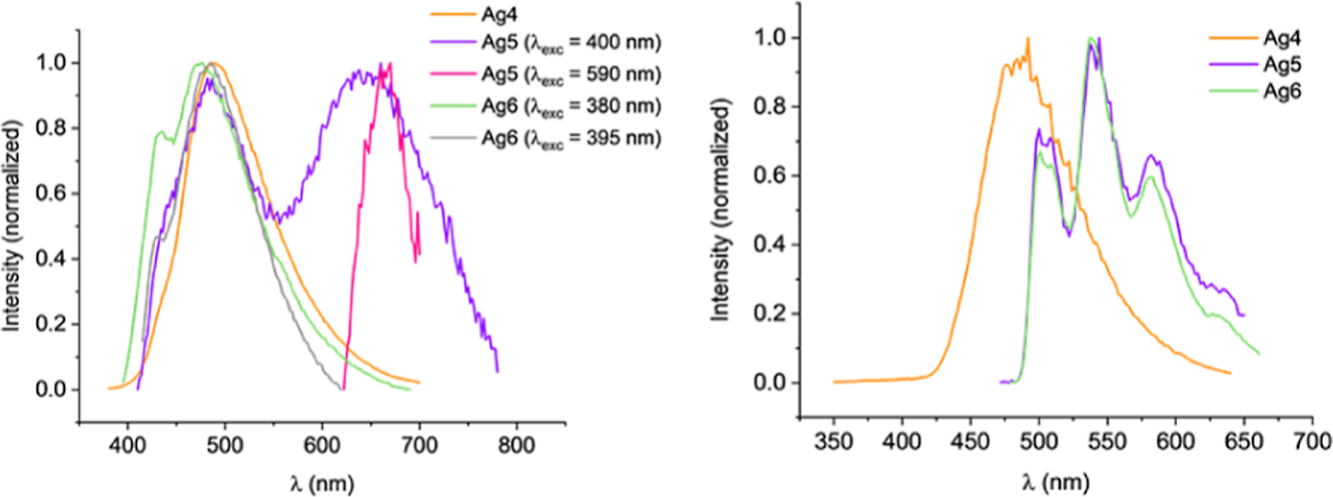
Emission spectra of the mononuclear complexes **Ag4–Ag6** in the solid state at room temperature (left) and 77 K (right) (see Supporting Information for individual spectra and more details).

**Figure 13:**
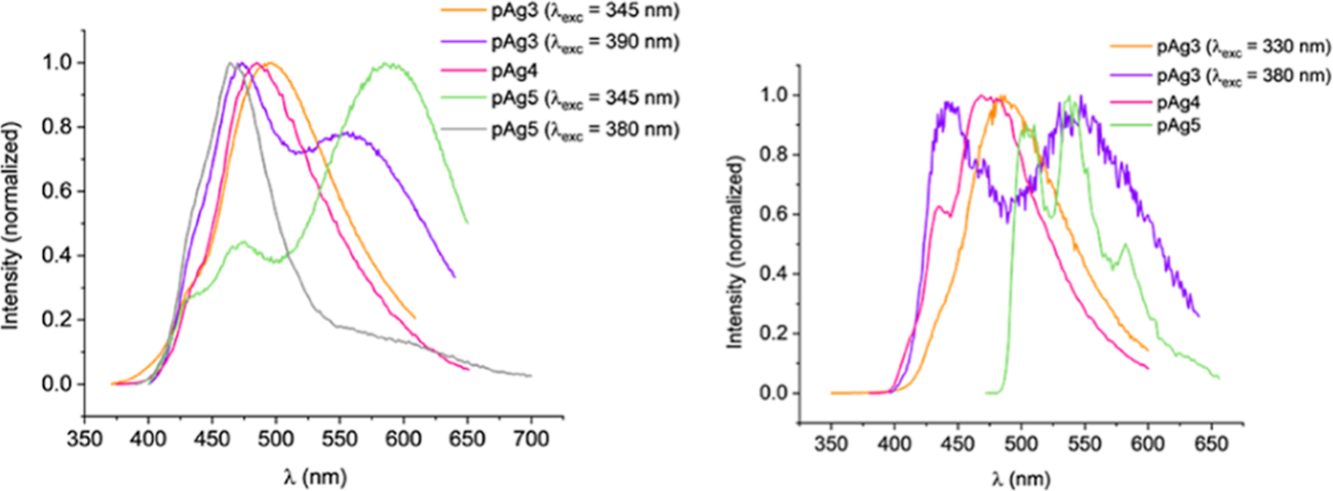
Emission spectra of the polynuclear complexes **pAg3–pAg5** in the solid state at room temperature (left) and 77 K (right) (see Supporting Information for individual spectra and more details).

**Figure 14:**
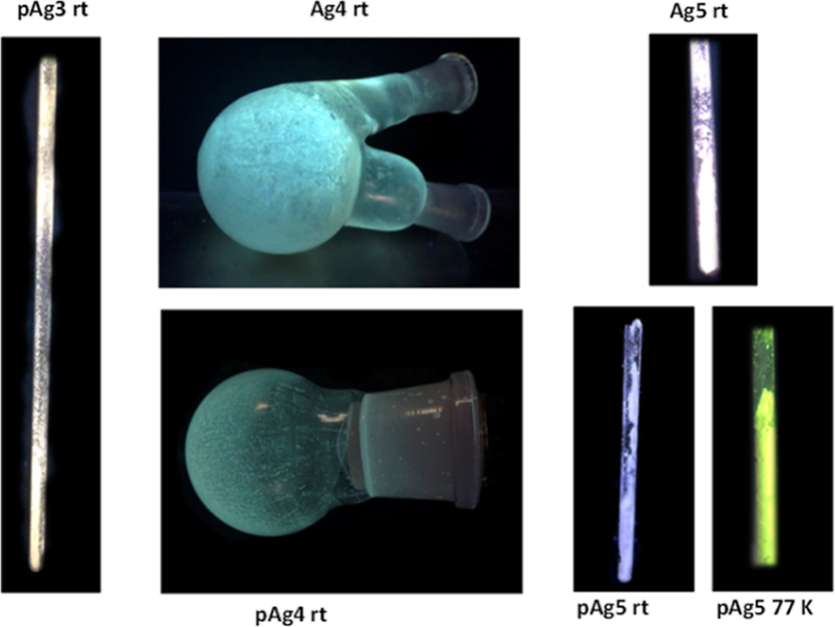
Images of a selection of the emissive complexes in the solid state, under UV light (350 nm).

The presence of the smaller and electron-donor **R** methyl substituent as carbene wingtip, leads to not emissive mononuclear **Ag1**, **Ag2** and polynuclear **pAg1**, **pAg2** species. In contrast, complexes with electron-withdrawing and more sterically demanding wingtips (**R** = Bz and NaphCH_2_) lead to emissive complexes, except for **Ag3** (see Tables 1 and 2; Figures 12–14).

**Table 1:** Photoluminescence Data for Complexes **Ag4–Ag6** as Solid Powder^f^

**AgN**	*T*	λ_ex_^a^	λ_em_^a^	τ (ms)	Φ (%)
**Ag4**	rt	350	490	0.018^c^	24
	77 K	330	492	0.061	
**Ag5**	rt^b^	400/510	485, 660/660	0.126/0.144	3^e^
	77 K	340	542	47.00	
**Ag6**	rt^b^	380/395	435/477	0.220^d^	<1^e^
	77 K	340	540	33.55	

a λ_ex_ = excitation maximum, λ_em_ = emission maximum.

b Dual emission separated by forward slash. Emission at the right part of the forward slash corresponds with data at the right part in the excitation and lifetime columns.

c Data were fitted to a monoexponential equation. The rest were fitted to a double exponential (see Supporting Information).

d Lifetime observed is the same as an independent analysis of the two emissions could not be achieved due to the proximity of the maxima (see Supporting Information).

e A broad band is observed and it is not possible to calculate the quantum yield only for one component, value correspond both to 380 and 400 excitation wavelength.

f rt = room temperature.

**Table 2:** Photoluminescence Data for Complexes **pAg3–pAg6** as Solid Powder^g^

**pAgN**	*T*	λ_ex_^a^	λ_em_^a^	τ (ms)	Φ (%)
**pAg3**	rt^b^	345/390	495/560	0.097/0.076^e^	6^e^
	77 K^b^	380/330	443, 486/486	–/23.94	
**pAg4**	rt	345	490	0.185	7^f^
	77 K	320	480	26.90^c^	
**pAg5**	rt^b^	345/400	586/468	–/0.105*	6^f^
	77 K^b^	335/375	555/445	32.62/–	
**pAg6**	77 K^d^	340	540	10.33	

a λ_ex_ = excitation maximum, λ_em_ = emission maximum.

b Dual emission separated by forward slash. Emission at the right part of the forward slash corresponds with data at the right part in the excitation and lifetime columns.

c Data were fitted to a monoexponential equation. The rest were fitted to a double exponential (see Supporting Information).

d Very week emission.

e A broad band is observed in which the second component grows upon exciting from 320 to 400 nm which complicates lifetime fitting and it is not possible to calculate the quantum yield only for one component, values range from 3 to 6%.

f Both bands appear at both excitation wavelengths which do not allow independent treatment and values of quantum yields range from 3 to 6% when moving from 340 to 400 nm.

g rt = room temperature.

##### Bis(carbene)silver Complexes [**AgN**] (*N* = 4–6)

Complex **[Ag(Bz-Im-Py)**_**2**_**]OTf** (**Ag3**) is not emissive, but **[Ag(Bz-Im-2-ClPy)**_**2**_**]OTf** (**Ag4**) displays blue emission at ca. 490 nm at room temperature and 77 K with lifetimes in the microsecond range and quantum yield of 24% at room temperature.

Compounds **[Ag(NaphCH**_**2**_**-Im-2-ZPy)**_**2**_**]OTf** (Z = H, **Ag5**; Z = Cl, **Ag6**) are emissive at room temperature and 77 K. Dual emission at room temperature is observed for both complexes. The two components almost overlap in the blue region for **Ag5**, but appear at very different energies for **Ag6**, one at 485 nm (cyan) and the other at 660 nm (red). At 77 K these compounds with the 2-naphthylmethyl carbene wingtip only show one structured band in the green region, in which the local maxima of the vibronic structure are separated about 1300 cm^–1^.^42,43^

Cooling causes an important increase in the lifetime of **Ag5** and **Ag6**, from the microsecond to millisecond range, not observed for **Ag4**. Quantum yields ≤3% have been measured at room temperature for **Ag5** and **Ag6**.

##### Polynuclear Species [**pAgN**] (*N* = 3–6)

Complex **pAg6** is very weakly emissive at 77 K and not emissive at room temperature. Compounds **pAg3–pAg5** are luminescent both at room temperature and 77 K and their quantum yields at room temperature are about 7% (Table 2 and Supporting Information). Emission maxima for these complexes cover the blue-yellow region. A slight blue shift in the emission maxima and an important increase in the lifetimes are observed upon cooling. Compounds **pAg3** and **pAg5** show dual emission both at room temperature and at 77 K, one of the emissions display a vibronic profile with the same spacing between local maxima as that observed for **Ag5** and **Ag6**, approximately 1300 cm^–1^

**Figure 15:**
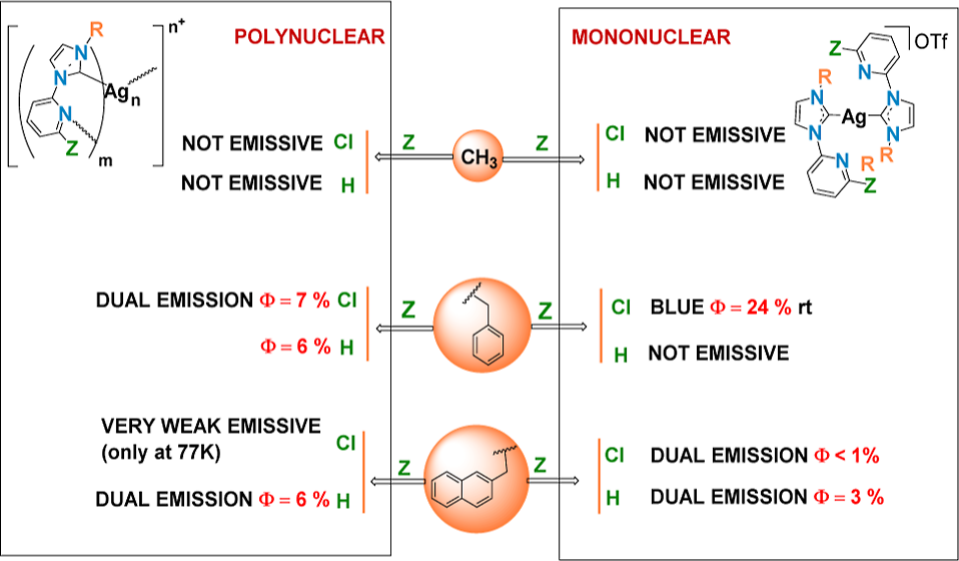
Resume of the emissive behavior of complexes **Ag1–Ag6** and **pAg1–pAg6**. Values in red correspond to Φ (%) in the solid state at room temperature.

Figure 15 presents a global perspective of the photoluminescent behavior of the complexes **AgN** and **pAgN** and state that the carbene wingtip has a significant impact on the emissive behavior of these compounds, as the **R** methyl donor group leads to no emissive mono or polynuclear compounds. Increasing the size of the wingtip leads to emissive complexes, except for **Ag3**.

Changing the **Z** substituent has different effect in quantum yield trends or the complexity of the spectra, depending on the wingtip and nuclearity.

#### Possible Origin of the Emissions

The role of Ag···Ag interactions in the emissive properties of **AgN** and **pAgN** complexes must be considered, as argentophilic interactions has been proposed as the origin of the emission in many silver–carbene complexes.^10^ Aggregation of different mononuclear units through silver···silver contacts or the formation of chains supported by these metallophilic interactions could favor intersystem crossing and enhance phosphorescent emissions but, these interactions do not appear to be responsible for the luminescent behavior of the complexes studied in this work. Several observations support this point. Complex **Ag3** is not emissive and crystallizes as dinuclear aggregates through intermolecular argentophilic contacts, not present in the emissive **Ag4** compound. Additionally, the nonemissive polynuclear compounds **pAg1** and **pAg2** consist of chains supported by metallophilic interactions. In contrast, **pAg3** is luminescent at 77 K, with a crystal structure featuring chains which contain silver···silver interactions, though these interactions do not interconnect all the silver atoms in the chain. Also, despite of their similar nuclearity and presence of Ag···Ag interactions, **pAg6** exhibits negligible luminescence only at 77 K, whereas **pAg4** is emissive both at room temperature and 77 K.

Thus, IL (metal modified), MLCT and, more probably, a mixed (IL/MLCT) character seems to be the most reasonable assignment for the observed emissions. This is consistent with the observation of vibronic structured bands, particularly in complexes with **R** = 2-naphthylmethyl at low temperature, which support the participation of the ligand in the orbitals responsible of the transitions leading to the emissions. The proposed origin also fits with the emission energies observed (blue-yellow region of the visible spectrum).^44^

Among reported examples, more energetic emissions are in many cases related with IL origin, while MLCT tend to lead less energetic emissions.

Dual emission is observed for some complexes, complicating the emission pattern. This fact is consistent with the presence of IL and MLCT excited states at similar energies. Such a dual behavior has been reported for other silver carbene complexes^45,46^ and can be proposed as the explanation for the emissions observed in the trinuclear complex **pAg5** and the polymeric compound **pAg3**. However, this is not the only possible origin for the dual emission. Excimer emissions are characterized by unstructured bands which appear at lower energies than those corresponding to the monomeric species and could also be the origin for the dual emission observed. The presence of π–π interactions in the crystal structure has been carefully considered, particularly given that excimer emissions have been reported for many silver carbene complexes in solution and, much more rarely, in the solid state,^30,47^ and are associated with the presence of π–π interactions.

Among the mononuclear species **AgN** dual emission is only observed for those with the naphthyl carbene wingtip **Ag5** and **Ag6** at room temperature, which would be consistent with excimer formation. Due to the higher size and electronic delocalization in the naphthyl group, compared with the phenyl group, π–π interactions are more likely to occur with the 2-naphthylmethyl substituent, potentially leading to excimer formation.

Excimer formation is discarded for **pAg5** as the data are not consistent with such origin. The not structured band appears at higher energy than that of the structured band and, as explained above the excimer emission (not structured) is expected to appear at lower energies than that of the monomeric species.

Despite their similar chemical structures, complexes **pAg4** and **pAg6** show markedly different luminescent behavior with **pAg6** showing poor luminescence and only at low temperature. A possible explanation for this difference could be the deactivation processes facilitated by a more flexible wingtip in **pAg6**, compared with **pAg4**. This hypothesis aligns with the higher quantum yield observed for the monomeric species **Ag4** (**Z** = Bz), compared to those obtained for **Ag5** and **Ag6** (**Z** = NaphCH_2_).

It is also noticeable that complex **Ag3** (**R** = H, **Z** = Bz) is not luminescent, compared with the emissive **Ag4** (**R** = Cl, **Z** = Bz) which displays the highest quantum yield. A possible explanation could be found in the differences observed when comparing silver environment in their crystal structures. In **Ag3** the silver atom displays linear geometry, and dimers are formed through Ag···Ag intermolecular interactions. In **Ag4** the chloride atoms of both ligands point to the silver center at distances of 3.995(2) and 4.001(3) Å, while the Ag–N distances are shorter than those found in **Ag3**. The greater rigidity around the silver center in **Ag4** could avoid deactivation processes and could lead to this different behavior.

The lifetime of compound **Ag4** is in the microsecond range both at room temperature and 77 K. In contrast, **Ag5** and **Ag6** show a significant increase in lifetime upon cooling, reaching values in the millisecond range, which is consistent with a different multiplicity of the excited state that originates the emission.

The highest quantum yield is observed for **Ag4** with R = benzyl and Z = Cl (24%). Not many photoluminescent studies can be found for silver carbene-NHC complexes in the solid state^45,46,48−52^ to be compared with those resumed in Figure 4. High quantum yields (up to 85%) have been found in the solid state or film for silver complexes with NHC ligands and a different ancillary ligand. As far as we are aware, the value observed for **Ag4** exceeds those reported for silver–carbene complexes without ancillary ligands in the solid state.

## Conclusions

The structural and emissive properties of mono and polynuclear silver complexes, derived from NHC imidazolium salts [R-HIm-2-ZPy]OTf (**R** = Me, Bz, NaphCH_2_; **Z** = H, Cl), are significantly influenced by modifications to the wingtip (**R**) and/or the substituent in the pyridine ring (**Z**).

The choice of **R** greatly impacts the structural arrangement of polymeric complexes, particularly evident in the formation of chains with the less sterically demanding methyl wingtip. The **Z** substituent at the pyridine ring can further modulate this behavior. For instance, with the medium steric demanding benzyl **R** group, a dinuclear species is obtained when **Z** = Cl, contrasting with a linear chain formation when **Z** = H.

Crystal structures of the mononuclear bis(carbene) complexes **Ag3** and **Ag4** ([Ag{L*^n^*}_2_]OTf, *n* = 3, 4) with the benzyl **R** group illustrate these effects, highlighting how altering **Z** from H to Cl eliminates argentophilic intermolecular interactions.

While the smaller and electron-donor methyl wingtip results in nonemissive complexes, the bulkier NaphCH_2_ group yields lower quantum yields compared to the Bz group. Emissive properties seem not to be ruled by the presence or argentophilic interactions being IL/MLCT the most feasible origin for the emissions. Certain complexes show emission spectra characterized by a complex pattern consisting of dual emission, which may be explained by the presence of IL and MLCT excited states at similar energies, but also by excimer formation for **Ag5** and **Ag6**.

Remarkably, compound **Ag4**, featuring the benzyl wingtip and a chloride substituent at the pyridine ring, achieves the highest reported quantum yield (24%) in the solid state for a silver NHC compound without ancillary ligands.

The overall results indicate that the carbene **R** wingtip predominantly determines both the structural nuclearity and the emissive quantum yields, with the potential for further modulation through the choice of the **Z** substituent. These findings may support the synthesis of new silver carbene complexes.

## Experimental Section

### Instrumentation

NMR spectra were carried out in a Bruker AV 400 or 300 in CD_3_CN and chemical shifts (ppm) reported relative to the solvent peaks of the deuterated solvent.^53^ A Bruker MicroToF-Q spectrometer was used for high-resolution mass spectra-ESI (HRMS-ESI) equipped with an API-ESI source and a QTOFmass analyzer, both allowing a maximum error in the measurement of 5 ppm. Steady-state photoluminescence spectra and lifetime measurements were recorded with a FluoTime300 PicoQuant spectrometer as powder samples, placed in a quartz tube. A liquid nitrogen dewar assembly was used for the studies at 77 K. Quantum yields were measured by the absolute method using a Hamamatsu Quantaurus-QY C11347 compact one-box absolute quantum yield measurement system. In order to prove the reproducibility of the measurements, three or more measurements were carried out for each compound with different amount of solid powder sample. Through studies carried out for different substances using both, the absolute method and the comparative one the relative uncertainty for the absolute method has been determined as less than 6%.^54^

### Crystallography

Crystals suitable for X-ray studies were obtained by diffusion of *n*-hexane over a solution of the corresponding compound in acetone, or the diffusion of Et_2_O over a solution the compound in dichloromethane (**pAg3**) or acetone (**pAg1**). Crystals were mounted on a MiTeGen Crystal micromount and transferred to the cold gas stream of a Bruker D8 VENTURE (2) diffractometer. Data were collected using monochromated Mo Κα radiation (λ = 0.71073 Å). Scan type ω. Absorption correction based on multiple scans were applied with the program SADABS.^55^ The structures were refined on F^2^ using the program SHELXL-2018.^56^ CCDC deposition numbers 2365118 (**Ag3**), 2365119 (**Ag4**), 2365120 (**pAg1**), 2365121 (**pAg2**), 2365122 (**pAg3**), 2365123 (**pAg4**), 2365124 (**pAg5**), 2365125 (**pAg6**) contain the corresponding supplementary crystallographic data. These data can be obtained free of charge by The Cambridge Crystallography Data Center.

### Powder X-ray Measurements

Data have been recorded at room temperature using a RIGAKU Ru2500 diffractometer provided with a rotating anode. Measurement conditions: Cu anode with a graphite monocromator, Cu Kα radiation, 40 kV and 80 mA, 2Theta 3° to 50°, step = 0.03°, *t* = 1 s/step.

### General Synthetic Procedures

The synthesis of both ligands and complexes was carried out under Ar atmosphere, using Schlenck techniques. Solvents were used as received without purification or drying, but all of them were degassed before being used in the corresponding chemical procedure. The starting materials, imidazole, 2-bromopyridine or 2-chloro-6-bromopyridine, MeI, benzyl chloride, 2-naphthylbromomethyl and AgOTf are commercially available and were used as received.

The preparation of the imidazolium salts **(HL**^**1**^**)OTf-(HL**^**6**^**)OTf** was carried out from HIm-Py (**1**)^57^ or HIm-2-ClPy (**2**)^58^ by reaction with RX (R = Me, X = I; R = benzyl, X = Cl or 2-naphthylmethyl, X = Br) and AgOTf through adapted methods from those published for **(HL**^**n**^**)PF**_**6**_ salts. NMR data are only given for those not reported, as those of the **(HL**^**n**^)**OTf** salts fit those described for the corresponding reported **(HL**^**n**^**)PF**_**6**_ salts.

### Synthesis of the Imidazolium Salts: **(HL**^**n**^**)OTf**

Synthesis of **(HL**^**1**^**)OTf** and **(HL**^**2**^**)OTf** were prepared following the reported method for **(HL**^**1**^**)PF**_**6**_ by using AgOTf in CH_2_Cl_2_ instead of NBu_4_PF_6_ in water.^59^ NMR ^1^H data for **(HL**^**n**^**)OTf** (*n* = 1, 3 and 4) may be compared with those reported for **(HL**^**n**^**)PF**_**6**_.

Synthesis of [Me-HIm-2-ZPy]OTf [(HL^1^)OTf, (Z = H); (HL^2^)OTf, (Z = Cl)] was achieved from the corresponding iodide imidazolium salts [Me-HIm-2-ZPy]I, prepared through the literature adapted method: a mixture of HIm-2-ZPy [Z = H (**1**) (290.3 mg, 2.0 mmol), Z = Cl (**2**) (180.0 mg, 1.0 mmol)] and iodomethane [Z = H (**1**) (0.5 mL, 8.0 mmol), Z = Cl (**2**) (0.25 mL, 4.0 mmol)] was heated at 68 °C in THF overnight. Filtration of the resulting mixture gave a white powder which was washed with diethyl ether to afford the pure product [Me-HIm-2-HPy]I (448.2 mg, 1.56 mmol, yield: 78%) or [Me-Im-2-ClPy]I (273.6 mg, 0.85 mmol, yield: 85%).

#### Synthesis of [Me-Im-2-ZPy]OTf Salts

To a solution of [Me-HIm-2-ZPy]I [Z = H (322.4 mg, 2 mmol), X = Cl (**2**) (273.6 mg, 0.35 mmol)] in dichloromethane (10 mL) AgOTf [Z = H (565.2 mg, 2.2 mol), X = Cl (116.6 mg, 0.45 mol)] was added. The mixture was stirred for 3 h and filtered through Celite and the clear filtrate was concentrated to ca. 5 mL. Addition of *n*-hexane (ca. 10 mL) led to the precipitation of a white solid corresponding to **(HL**^**1**^**)OTf** (464.0 mg, 1.5 mmol, yield 75%) or **(HL**^**2**^**)OTf** (96.3 mg, 0.28 mmol, yield: 80%).

##### **HL**^**1**^**(OTf)**

^1^H NMR (300.1 MHz, CD_3_CN, rt, ppm): δ 9.32 (s, 1H), 8.58 (ddd, *J* = 4.9, 1.8, 0.9 Hz, 1H), 8.16–8.04 (m, 2H), 7.76 (dt, *J* = 8.2, 0.9 Hz, 1H), 7.62–7.52 (m, 2H), 3.97 (s, 3H).

##### **HL**^**2**^**(OTf)**

^1^H NMR (300.13 MHz, CD_3_CN, rt, ppm): δ 9.37 (s, 1H), 8.15–8.03 (m, 2H), 7.76 (d, *J* = 8.1 Hz, 1H), 7.65–7.55 (m, 2H), 3.97 (s, 3H).

^13^C{^1^H}-APT NMR (74.5 MHz, CD_3_CN, rt, ppm): δ 151.3 (s, CR_4_), 144.3 (s, CH), 136.1 (s, CR_4_), 126.7 (s, CH), 125.9 (s, CH), 120.3 (s, CH), 113.9 (S, CH), 37.6 (s, CH_3_).

^19^F NMR (282.4 MHz, CD_3_CN, rt, ppm): δ −79.3 (s, OTf).

HRMS (ESI-QTOF) *m*/*z*: [M – OTf]^+^ calcd for C_9_H_9_ClN_3_, 190.0480; found, 190.0474.

Synthesis of (HL^3^)OTf and (HL^4^)OTf was achieved through modification of the methods reported for the corresponding **(HL**^**n**^**)PF**_**6**_(18,54) salts, by using AgOTf in dichloromethane instead of KPF_6_ in acetone. Starting from 1 mmol of **(HL**^**n**^**)Cl** [*n* = 3, 272.7 mg; *n* = 4, 307.2 mg] and AgOTf (1.1 mmol, 282.7 mg).

##### **(HL**^**3**^**)OTf**

297.5 mg, 77% yield. ^1^H NMR (300.1 MHz, CD_3_CN, rt, ppm): δ 9.50 (t, *J* = 1.7 Hz, 1H), 8.57 (ddd, *J* = 4.8, 1.8, 0.7 Hz, 1H), 8.17–8.03 (m, 2H), 7.79 (d, *J* = 8.2 Hz, 1H), 7.63–7.37 (m, 7H), 5.48 (s, 2H).

##### **(HL**^**4**^**)OTf**

302.9 mg, 72% yield. ^1^H NMR (300.1 MHz, CD_3_CN, rt, ppm): δ 9.95 (t, *J* = 1.7 Hz, 1H), 8.16–8.04 (m, 2H), 7.89 (dd, *J* = 8.1, 0.6 Hz, 1H), 7.74–7.37 (m, 7H), 5.52 (s, 2H).

Synthesis of (HL^5^)OTf and (HL^6^)OTf was carried out though reaction of the corresponding imidazolium bromide salt [NaphCH_2_-HIm-ZPy]Br (Z = H, Cl), which were prepared by refluxing a mixture of HIm-Py (**1**) (730 mg, 5 mmol) or HIm-2-ClPy (**2**) (903.05 mg, 5 mmol) and 2-naphthylbromomethyl (1.216 g, 5.5 mmol) in acetonitrile overnight. After cooling, the solution was evaporated to a minimum volume. Upon addition of diethyl ether (ca. 10 mL) a precipitate was afforded, corresponding to the pure product [NaphCH_2_-HIm-Py]Br (1616.0 mg, 4.4 mmol, yield 88%) or [NaphCH_2_-HIm-2-ClPy]Br (1530.3 mg, 3.8 mmol, yield 76%).

#### Preparation of the [NaphCH_2_-HIm-2-ZPy]OTf Salts

To a solution of [NaphCH_2_-HIm-Py]Br (734.0 mg, 2 mmol) or [NaphCH_2_-HIm-2-ClPy]Br (805.4 mg, 2 mmol) in dichloromethane AgOTf (643 mg, 2.5 mmol) was added. The mixture was stirred for 3 h and filtered through Celite. The clear filtrate was concentrated to ca. 5 mL. Addition of *n*-hexane (ca. 10 mL) led to the precipitation of **(HL**^**5**^**)OTf** (742.1 mg, 1.7 mmol, yield 85%) or **(HL**^**6**^**)OTf** (660.7 mg, 1.4 mmol, yield 70%) as pale yellow solids.

##### **(HL**^**5**^**)OTf**

^1^H NMR (300.13 MHz, CD_3_CN, rt, ppm): δ 9.53 (t, *J* = 1.8 Hz, 1H), 8.56 (ddd, *J* = 4.9, 1.8, 0.9 Hz, 1H), 8.18–8.11 (m, 1H), 8.07 (ddd, *J* = 8.2, 7.5, 1.8 Hz, 1H), 8.01 (s, 1H), 7.99–7.85 (m, 3H), 7.77 (dt, *J* = 8.2, 0.9 Hz, 1H), 7.64 (t, *J* = 1.8 Hz, 1H), 7.60–7.47 (m, 4H), 5.63 (s, 2H).

^13^C{^1^H}-APT NMR (74.5 MHz, CD_3_CN, rt, ppm): δ 150.3 (s, CH), 147.3 (s, CR_4_), 141.4 (s, CH), 135.2 (s, CR_4_), 134.3 (s, CR_4_), 134.2 (s, CR_4_), 131.8 (s, CR_4_), 130.1 (s, CH), 129.5 (s, CH), 129.0 (s, CH), 128.7 (s, CH), 128.1 (s, CH), 127.9 (s, CH), 126.7 (s, CH), 126.3 (s, CH), 124.4 (s, CH), 120.8 (s, CH), 115.1 (s, CH), 54.6 (s, CH_2_).

^19^F NMR (282.4 MHz, CD_3_CN, rt, ppm): δ −79.2 (s, OTf).

HRMS (ESI-QTOF) *m*/*z*: [M – OTf]^+^ calcd for C_19_H_16_N_3_, 286.1339; found, 286.1332.

##### **(HL**^**6**^**)OTf**

^1^H NMR (300.1 MHz, CD_3_CN, rt, ppm): δ 9.49 (t, *J* = 1.7 Hz, 1H), 8.15–7.89 (m, 6H), 7.74 (dd, *J* = 8.0, 0.6 Hz, 1H), 7.67–7.51 (m, 5H), 5.62 (s, 2H).

^13^C{^1^H}-APT NMR (74.5 MHz, CD_3_CN, rt, ppm): δ 151.2 (s, CH), 144.3 (s, CR_4_), 135.6 (s, CH), 134.4 (s, CR_4_), 134.2 (s, CR_4_), 131.6 (s, CR_4_), 130.1 (s, CH), 129.6 (s, CH), 129.0 (s, CH), 128.8 (s, CH), 128.1 (s, CH), 127.9 (s, CH), 126.8 (s, CH), 126.7 (s, CH), 124.7 (s, CH), 120.9 (s, CH), 114.0 (s, CH), 54.7 (s, CH_2_).

^19^F NMR (282.4 MHz, CD_3_CN, rt, ppm): δ −79.3 (s, OTf).

HRMS (ESI-QTOF) *m*/*z*: [M – OTf]^+^ calcd for C_19_H_15_Cl_3_N_3_, 320.0949; found, 320.0947.

### Synthesis of [Ag(NHC)_2_]OTf Complexes **Ag1–Ag6**

All reactions were protected from light.

The synthesis of [Ag(Me-Im-Py)_2_]PF_6_, analogous to the salt **Ag1**, but with PF_6_^–^ anion, instead of OTf^–^ was reported by reaction of the imidazolium PF_6_^–^ salt and Ag_2_O^20^ and NMR data fit with those obtained for **Ag1**.

**AgN** (*N* = 1–6) complexes have been synthesized from the **(HL**^**n**^**)OTf** salt and AgOTf: to a solution of the corresponding salt (0.5 mmol: **(HL**^**1**^**)OTf**, 154.7 mg; **(HL**^**2**^**)OTf**, 171 mg; **(HL**^**3**^**)OTf**, 193.2 mg; **(HL**^**4**^**)OTf**, 210.5 mg; **(HL**^**5**^**)OTf**, 218.2 mg; **(HL**^**6**^**)OTf**, 235.5 mg) in dichloromethane, AgOTf (77.0 mg, 0.3 mmol) and Cs_2_CO_3_ (228.0 mg, 0.7 mmol) were added. The resulting suspension was stirred overnight at room temperature and filtered through bulk Celite. The filtrate was reduced to a minimum volume under vacuum. Addition of diethyl ether led to the precipitation of the corresponding complex as a white powder.

#### [Ag(Me-Im-Py)_2_]OTf, **Ag1**

208.0 mg. Yield: 72%. ^1^H NMR (300.1 MHz, CD_3_CN, rt, ppm): δ 8.4 (ddd, *J* = 4.9, 1.8, 0.9 Hz, 2H), 7.92 (ddd, *J* = 8.2, 7.3, 1.8 Hz, 2H), 7.87–7.78 (m, 4H), 7.46–7.34 (m, 4H), 3.95 (s, 6H).

#### [Ag(Me-Im-2-ClPy)_2_]OTf, **Ag2**

215.8 mg. Yield: 67%. HRMS (ESI-QTOF) *m/z*: [M – OTf]^+^ calcd for C_18_H_16_AgCl_2_N_6_, 492.9859; found, 492.9875.

^1^H NMR (300.1 MHz, CD_3_CN, rt, ppm): δ 7.91 (t, *J* = 8.0 Hz, 2H, H3), 7.81 (d, *J* = 2.0 Hz, 2H, H6), 7.76 (dd, *J* = 8.0, 0.7 Hz, 2H, H2), 7.50–7.36 (m, 4H, H7, H4), 3.97 (s, 6H, H9).

^13^C{^1^H}-APT NMR (74.5 MHz, CD_3_CN, rt, ppm): δ 151.8 (s, C5), 150.7 (s, C1), 143.8 (s, C3), 125.4 (s, C7), 125.2 (s, C4), 120.9 (s, C6), 114.9 (s, C2), 40.7 (s, C9).

^19^F NMR (282.4 MHz, CD_3_CN, rt, ppm): δ −79.3 (s, OTf).

#### [Ag(Bz-Im-Py)_2_]OTf, **Ag3**

257.9 mg. Yield: 76%. HRMS (ESI-QTOF) *m*/*z:* [M – OTf]^+^ calcd for C_30_H_26_AgN_6_, 577.1264; found, 577.1263.

^1^H NMR (300.1 MHz, CD_3_CN, rt, ppm): δ 8.25 (ddd, *J* = 4.9, 1.8, 1.0 Hz, 2H, H1), 7.90–7.76 (m, 6H, H3, H4, H6), 7.42 (d, *J* = 2.0 Hz, 2H, H7), 7.35 (ddd, *J* = 6.6, 4.9, 1.8 Hz, 2H, H2), 7.32–7.22 (m, 10H, H-ph), 5.38 (s, 4H, H9).

^13^C{^1^H}-APT NMR (74.5 MHz, CD_3_CN, rt, ppm): δ 182.7 (s, C8), 151.8 (s, C5), 149.8 (s, C1), 140.6 (s, C3), 137.5 (s, C-ph), 129.9 (s, C-ph), 129.3 (s, C-ph), 128.6 (s, C-ph), 124.8 (s, C2), 123.9 (s, C7), 121.4 (s, C6), 116.3 (s, C4), 56.8 (s, C9).

^19^F NMR (282.4 MHz, CD_3_CN, rt, ppm): δ −79.3 (s, OTf).

#### [Ag(Bz-Im-2-ClPy)_2_]OTf, **Ag4**

207.1 mg. Yield: 64%. HRMS (ESI-QTOF) *m*/*z:* [M – OTf]^+^ calcd for C_30_H_24_AgCl_2_N_6_, 645.0485; found, 645.0484.

^1^H NMR (300.1 MHz, CD_3_CN, rt, ppm): δ 7.97–7.80 (m, 4H, H3, H6), 7.73 (d, *J* = 8.0 Hz, 2H, H2), 7.44 (d, *J* = 2.0 Hz, 2H, H7), 7.38 (d, *J* = 8.0 Hz, 2H, H4), 7.27 (s, 10H, H-ph), 5.38 (s, 4H, H9).

^13^C{^1^H}-APT NMR (74.5 MHz, CD_3_CN, rt, ppm): δ 151.4 (s, C1/C5), 150.4 (s, C1/C5), 143.5 (s, C3), 137.4 (s, C-ph), 129.9 (s, C-ph), 129.3 (s, C-ph), 128.7 (s, C-ph), 124.9 (s, C4), 124.4 (s, C7), 121.0 (s, C6), 114.5 (s, C2), 56.9 (s, C9).

^19^F NMR (282.4 MHz, CD_3_CN, rt, ppm): δ −79.3 (s, OTf).

#### [Ag(NaphCH_2_-Im-Py)_2_]OTf, **Ag5**

297.9 mg. Yield: 72%. HRMS (ESI-QTOF) *m*/*z:* [M – OTf]^+^ calcd for C_38_H_30_AgN_6_, 677.1577; found, 677.1557.

^1^H NMR (300.1 MHz, CD_3_CN, rt, ppm): δ 8.20 (ddd, *J* = 4.9, 1.8, 1.0 Hz, 2H, H1), 7.81 (d, *J* = 2.0 Hz, 2H), 7.76–7.66 (m, 8H), 7.65–7.59 (m, 4H), 7.49–7.36 (m, 6H), 7.25 (m, 4H), 5.37 (s, 4H, H9).

^13^C{^1^H}-APT NMR (74.5 MHz, CD_3_CN, rt, ppm): δ 182.8 (s, C8), 151.7 (s, C5), 149.6 (s, C1), 140.5 (s, CH), 134.9 (s, C-NaphCH_2_), 134.0 (s, C-NaphCH_2_), 133.8 (s, C-NaphCH_2_), 129.7 (s, CH), 128.62 (s, CH), 128.60 (s, CH), 127.8 (s, CH), 127.6 (s, CH), 127.5 (s, CH), 126.1 (s, CH), 124.7 (s, CH), 124.4 (s, CH), 121.0 (s, CH), 116.0 (s, CH), 56.7 (s, C9).

^19^F NMR (282.4 MHz, CD_3_CN, rt, ppm): δ −79.1 (s, OTf).

#### [Ag(NaphCH_2_-Im-2-ClPy)_2_]OTf, **Ag6**

340.6 mg. Yield: 76%. HRMS (ESI-QTOF) *m*/*z:* [M – OTf]^+^ calcd for C_38_H_28_AgCl_2_N_6_, 745.0798; found, 745.0786.

^1^H NMR (300.13 MHz, CD_3_CN, rt, ppm): δ 7.827.52 (m 14H), 7.52–7.35 (m, 6H), 7.35–7.22 (m, 4H), 5.34 (s, 2H, H9).

^13^C{^1^H}-APT NMR (74.48 MHz, CD_3_CN, rt, ppm): δ 151.2 (s, C5/C1), 150.2 (s, C5/C1), 142.3 (s, CH), 139.9 (C-NaphCH_2_), 139.9 (C-NaphCH_2_), 134.0 (C-NaphCH_2_), 133.9 (C-NaphCH_2_), 129.7 (s, CH), 128.6 (s, CH), 128.5 (s, CH), 128.4 (s, CH), 127.7 (s, CH), 127.5 (s, CH), 126.5 (s, CH), 124.8 (s, CH), 120.4 (s, CH), 114.2 (s, CH), 56.8 (s, C9).

^19^F NMR (282.4 MHz, CD_3_CN, rt, ppm): δ −79.3 (s, OTf).

### Synthesis of Polynuclear Complexes **pAg1–pAg6**

All reactions were protected from light.

To a solution of the corresponding **AgN** complex (0.2 mmol; **Ag1**, 115.1 mg; **Ag2**, 128.8 mg; **Ag3**, 143.3 mg; **Ag4**, 159.3 mg; **Ag5**, 165.5 mg; **Ag6**, 179.3 mg) in dichloromethane, AgOTf (77.0 mg, 0.3 mmol) was added. The resulting suspension was stirred for 1 h at room temperature. The mixture was filtered through bulk Celite and reduced to a minimum volume under vacuum. Addition of hexane led to the precipitation of the corresponding **pAgN** compound as a white solid. Yields have been calculated for the formation of the unit [Ag(NHC)]_2_OTf_2_.

#### **pAg1**

118.0 mg. Yield: 71%. ^1^H NMR (300.1 MHz, CD_3_CN, rt, ppm): δ 8.37 (ddd, *J* = 5.7, 1.8, 0.8 Hz, 1H), 7.97 (tdd, *J* = 7.4, 1.9, 0.7 Hz, 1H), 7.91–7.78 (m, 2H), 7.50–7.37 (m, 2H), 3.93 (s, 3H).

#### **pAg2**

115.9 mg. Yield: 64%. ^1^H NMR (300.1 MHz, CD_3_CN, rt, ppm): δ 7.92 (t, *J* = 7.9 Hz, 1H), 7.81 (d, *J* = 2.0 Hz, 1H), 7.75 (dd, *J* = 8.1, 0.6 Hz, 1H), 7.48–7.37 (m, 2H), 3.95 (s, 3H).

#### **pAg3**

116.2 mg. Yield: 60%. ^1^H NMR (300.1 MHz, CD_3_CN, rt, ppm): δ 8.26 (ddd, *J* = 4.9, 1.8, 0.9 Hz, 1H), 7.97–7.74 (m, 3H), 7.42 (d, *J* = 2.0 Hz, 1H), 7.38 (ddd, *J* = 7.4, 4.9, 1.1 Hz, 1H), 7.34–7.20 (m, 5H), 5.36 (s, 2H).

#### **pAg4**

120.1 mg. Yield: 57%. ^1^H NMR (300.1 MHz, CD_3_CN, rt, ppm): δ 7.89 (t, *J* = 7.9 Hz, 1H), 7.84 (d, *J* = 2.0 Hz, 1H), 7.73 (dd, *J* = 8.1, 0.7 Hz, 1H), 7.50–7.36 (m, 2H), 7.28 (s, 5H), 5.37 (s, 2H).

#### **pAg5**

143.3 mg. Yield: 65%. ^1^H NMR (300.1 MHz, CD_3_CN, rt, ppm): δ 8.25 (d, *J* = 4.9 Hz, 1H), 7.87–7.62 (m, 7H), 7.53–7.38 (m, 3H), 7.35–7.25 (m, 2H), 5.41 (s, 2H).

#### **pAg6**

173.5 mg. Yield: 75%. ^1^H NMR (300.1 MHz, CD_3_CN, rt, ppm): δ 7.83–7.57 (m, 7H), 7.49–7.39 (m, 3H), 7.36–7.25 (m, 2H), 5.36 (s, 2H).

## Supporting Information Available

NMR spectra, emission and excitation spectra, lifetime fitting curves, XRD spectra, crystal X-ray diagrams, bond distances and angles (PDF)
