# Supplementary material for: Understanding the Role of NHC Ditopic Ligand Substituents in the Molecular Diversity and Emissive Properties of Silver Complexes
Source: Inorg Chem. 2024 Oct 30;63(45):21699–710. doi: 10.1021/acs.inorgchem.4c02940 (PMC11558669; doi:10.1021/acs.inorgchem.4c02940)
Supplement: Supplementary file 1 — ic4c02940_si_001.pdf [file ic4c02940_si_001.pdf]

Supporting information

Understanding the role of NHC ditopic ligand substituents in the molecular diversity and emissive properties of silver complexes.

Irati Barriendos[a], Olga Crespo*[a] and M. Concepción Gimeno*[a]

[a] Departamento de Química Inorgánica, Instituto de Síntesis Química y Catálisis Homogénea (ISQCH). Universidad de Zaragoza-CSIC. E-50009 Zaragoza, Spain.

*Corresponding Author E-mail: ocrespo@unizar.es (O. C.), gimeno@unizar.es (M. C. G.)

NMR spectra.. S2

Emission and excitation spectra.. S38

Lifetime fitting curves.. S42

X-ray powder diffraction (XRD) spectra.. S74

Crystal X-ray data: Diagrams, bond distances (Å) and angles (°)...................... S76

NMR spectra

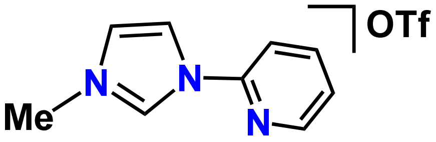

[Me-HIm-Py]OTf, (HL[1])OTf

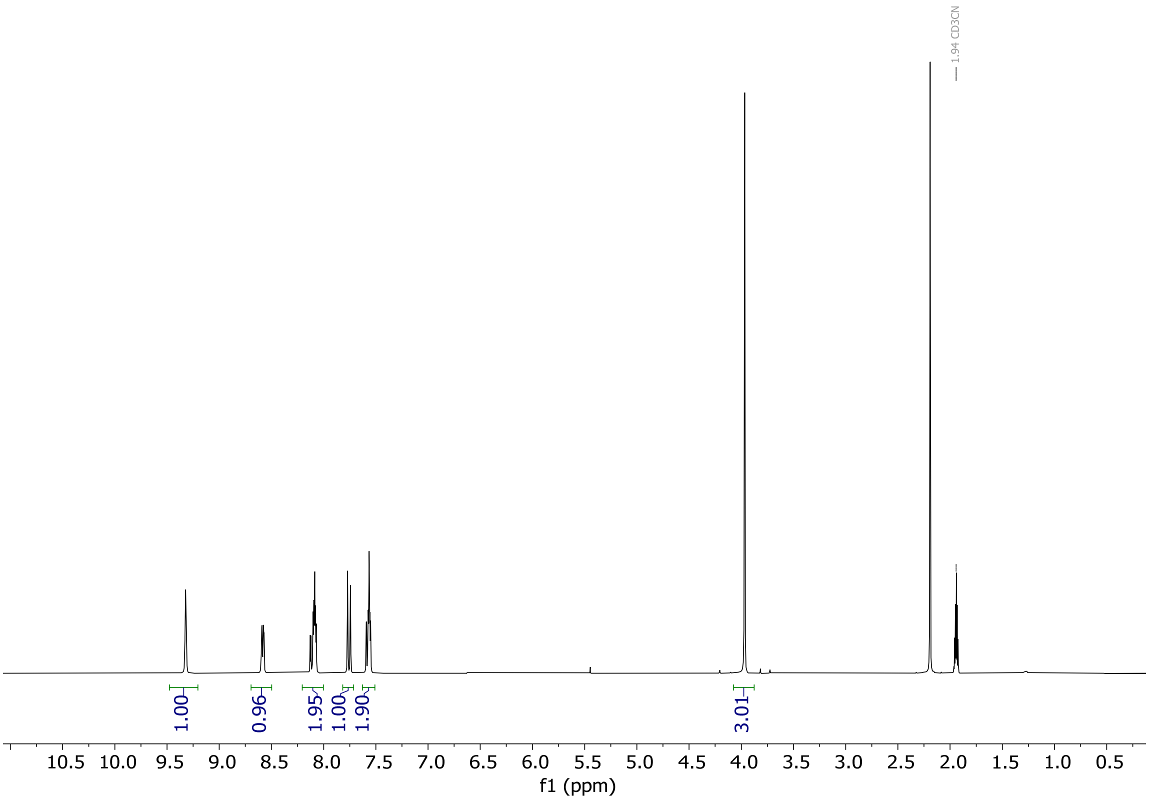

Figure S1. ^{1}H NMR spectrum of **(HL^{1})OTf**.

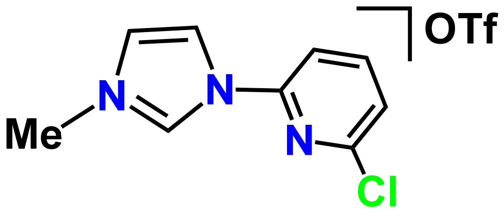

[Me-HIm-2-ClPy]OTf, *(HL²)OTf*

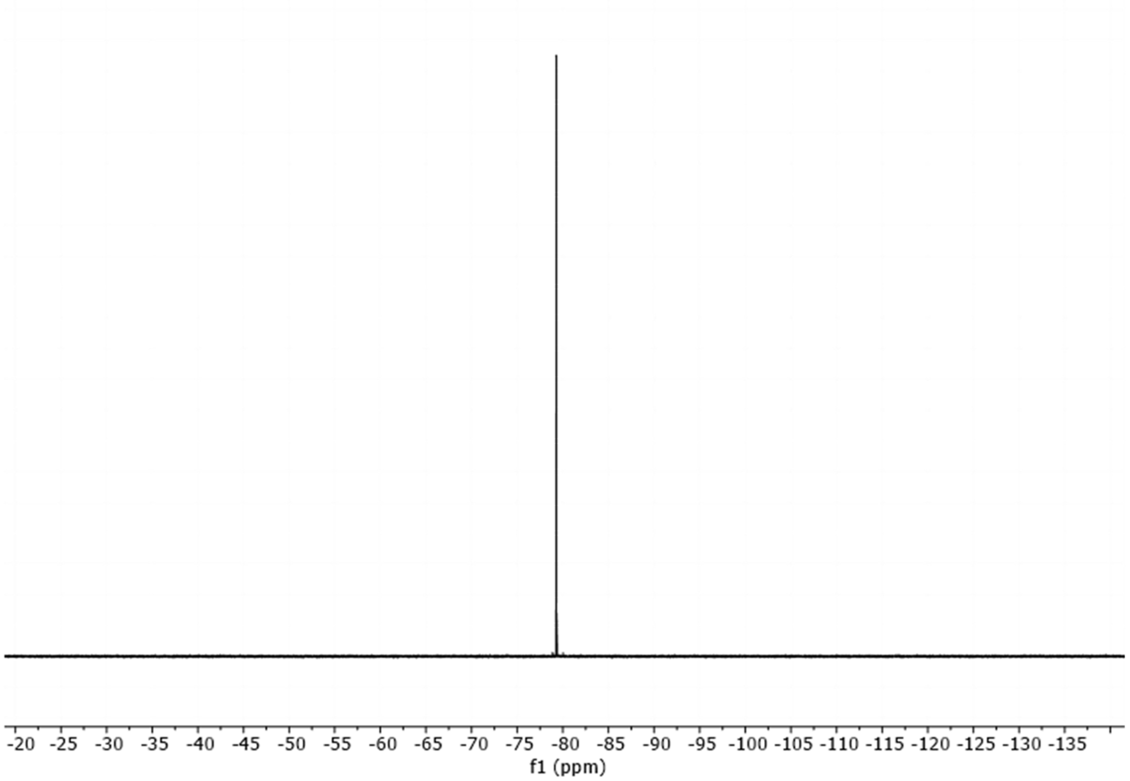

Figure S2. ^{19}F NMR spectrum of **(HL^2)OTf**.

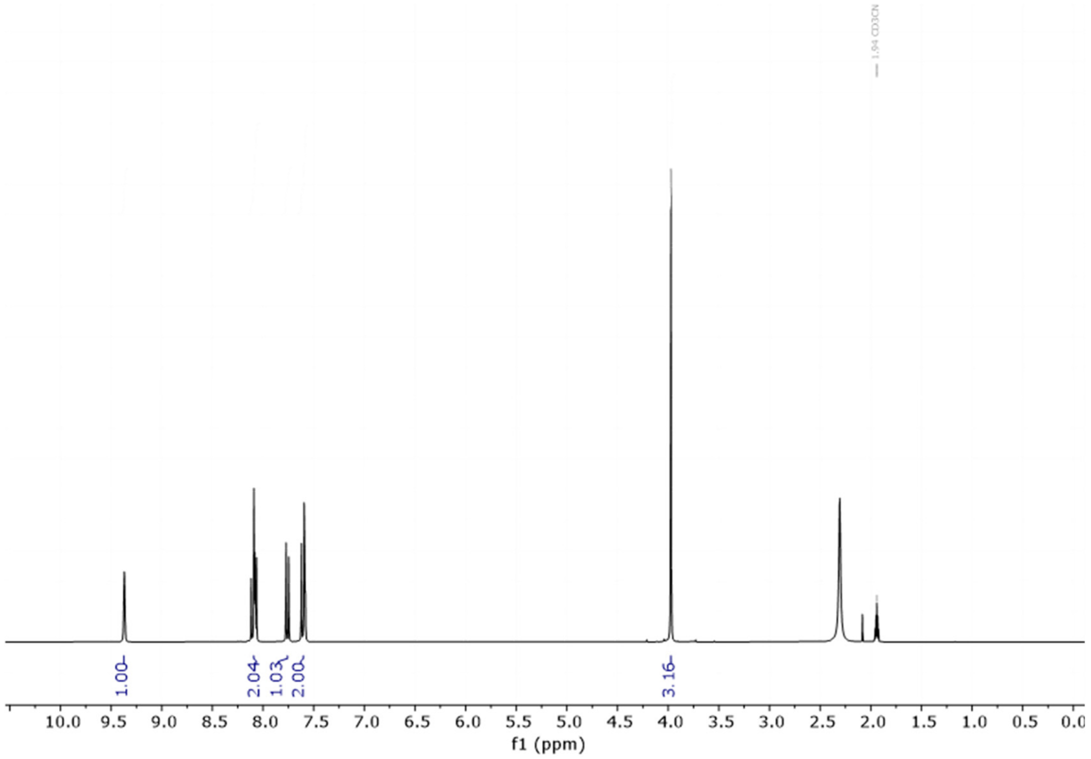

Figure S3. ^{1}H NMR spectrum of **(HL²)OTf**.

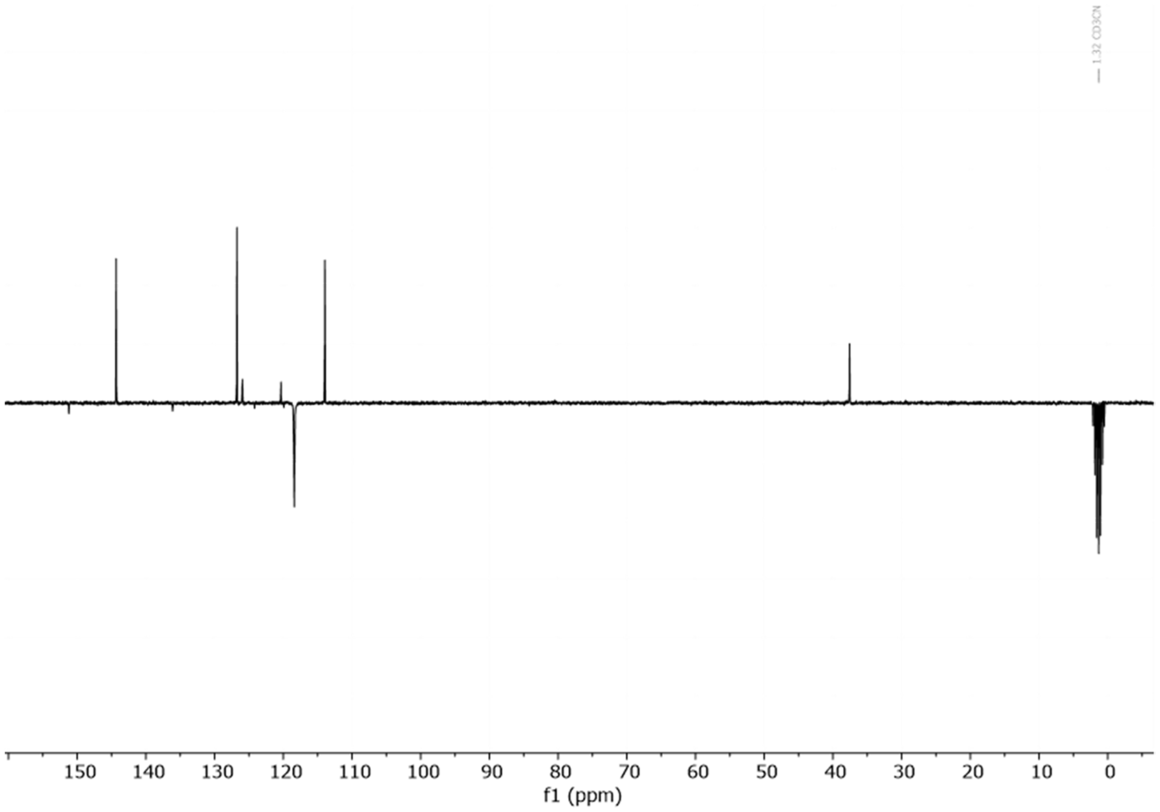

Figure S4. $^{13}C\{^{1}H\}$-APT NMR spectrum of **(HL²)OTf**.

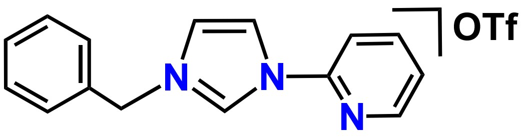

[Bz-HIm-Py]OTf, (HL[3])OTf

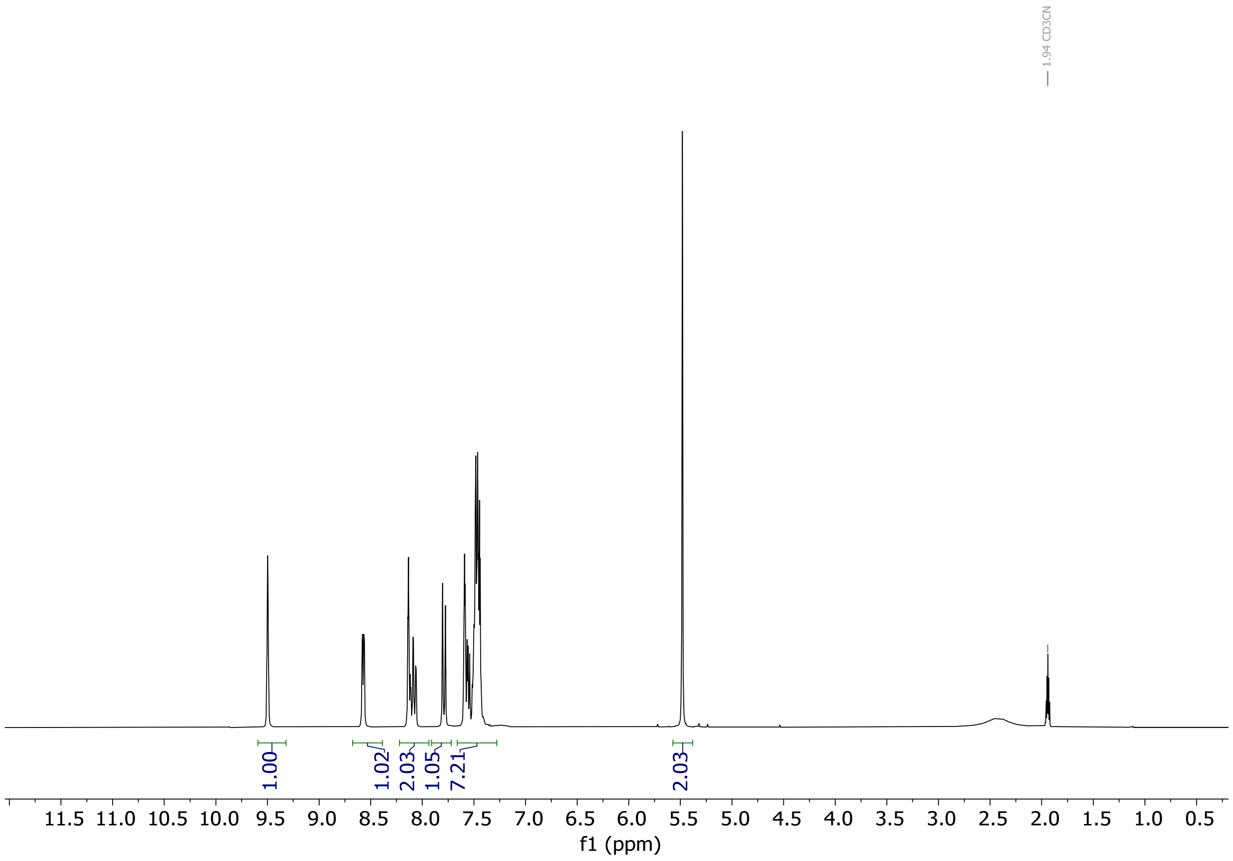

Figure S5. ^{1}H NMR spectrum of **(HL^{3})OTf**.

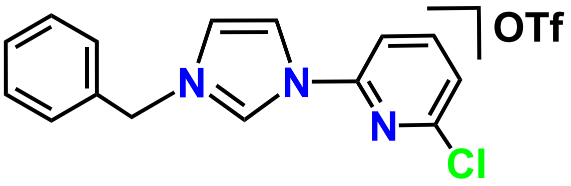

[Bz-HIm-2-ClPy]OTf, (HL⁴)OTf

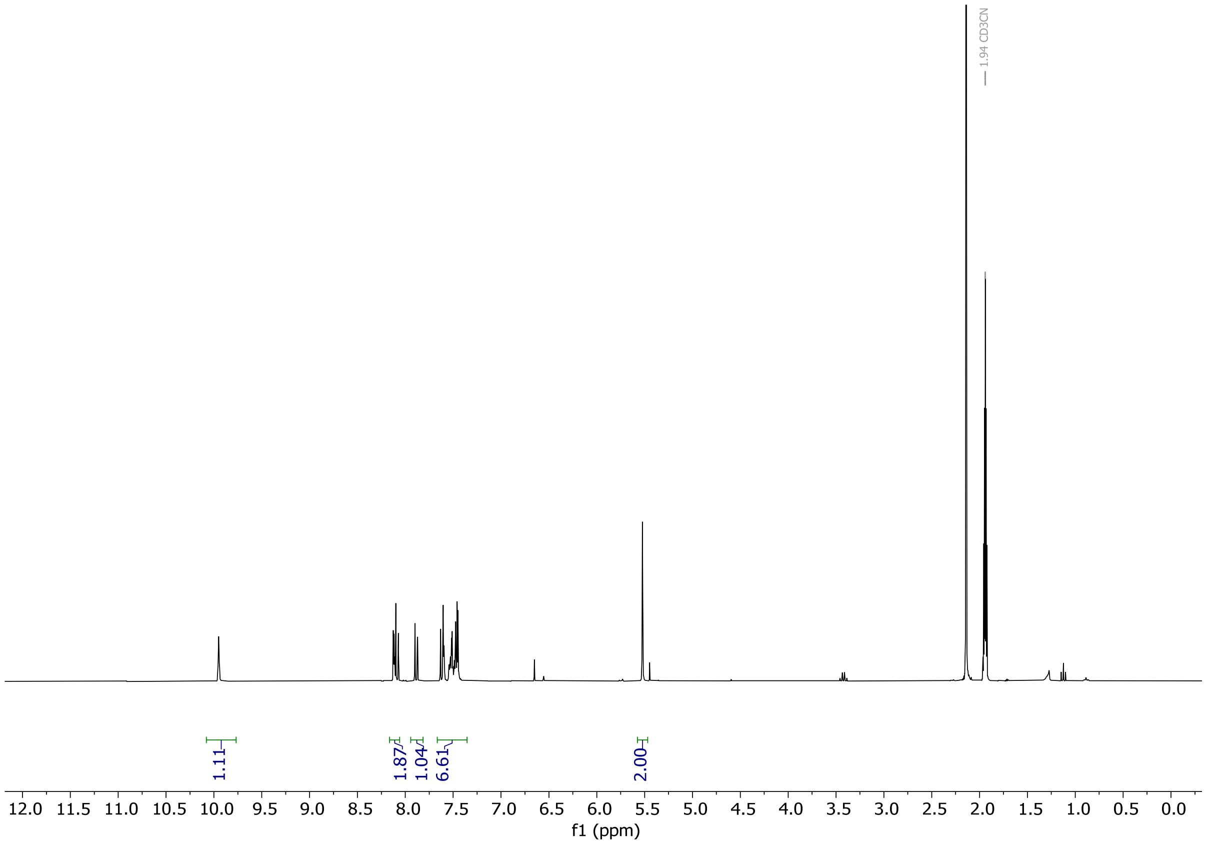

Figure S6. ^{1}H NMR spectrum of **(HL⁴)OTf**.

[NaphCH$_2$-HIm-Py]OTf, (HL^5)OTf

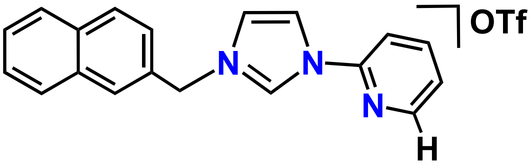

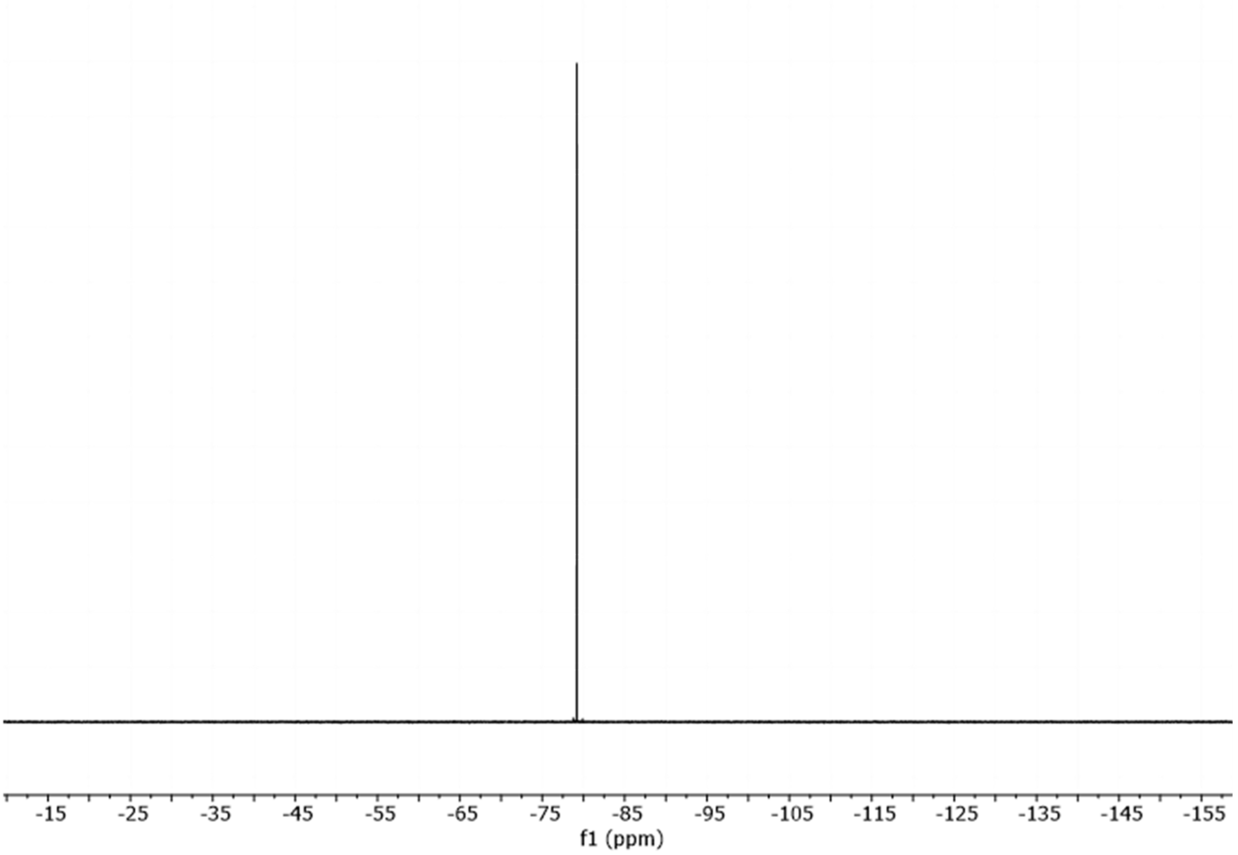

Figure S7. ^{19}F NMR spectrum of **(HL^5)OTf**.

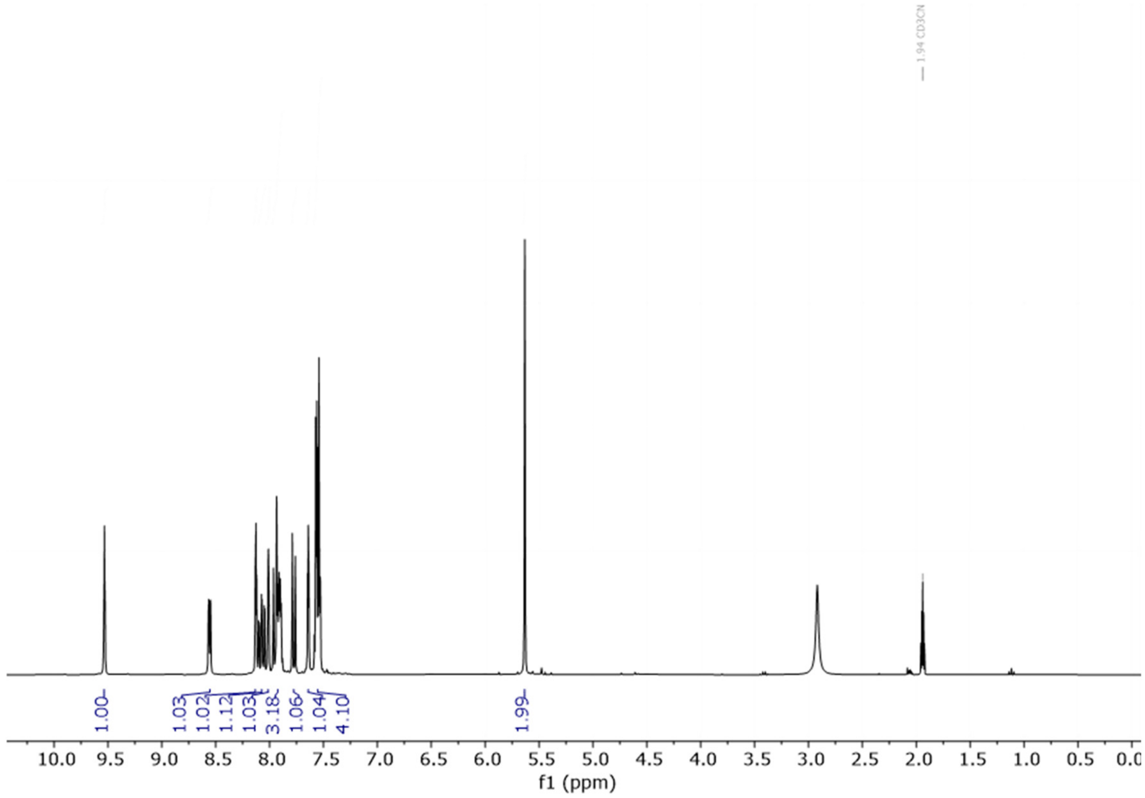

Figure S8. ^{1}H NMR spectrum of **(HL^5)OTf**.

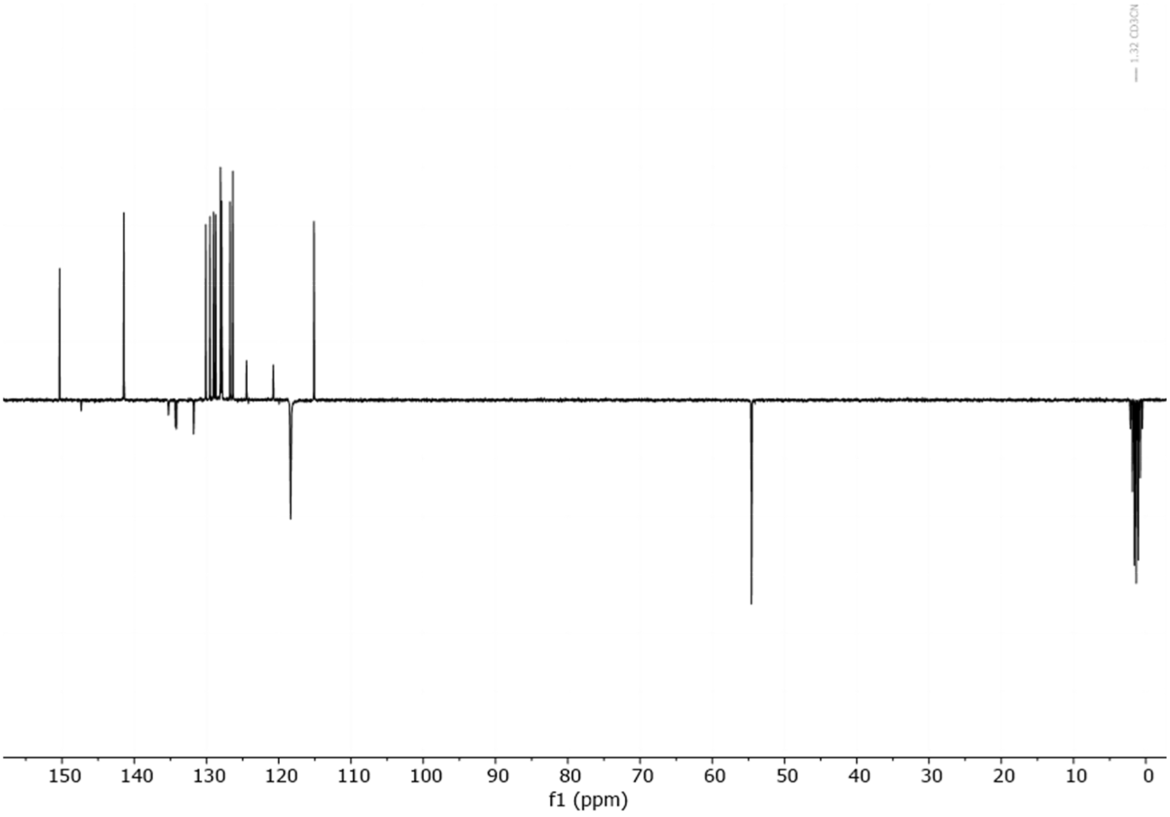

Figure S9. $^{13}C\{^{1}H\}$-APT NMR spectrum of **(HL^5)OTf.**

[NaphCH$_2$-HIm-2ClPy]OTf, (HL^6)OTf

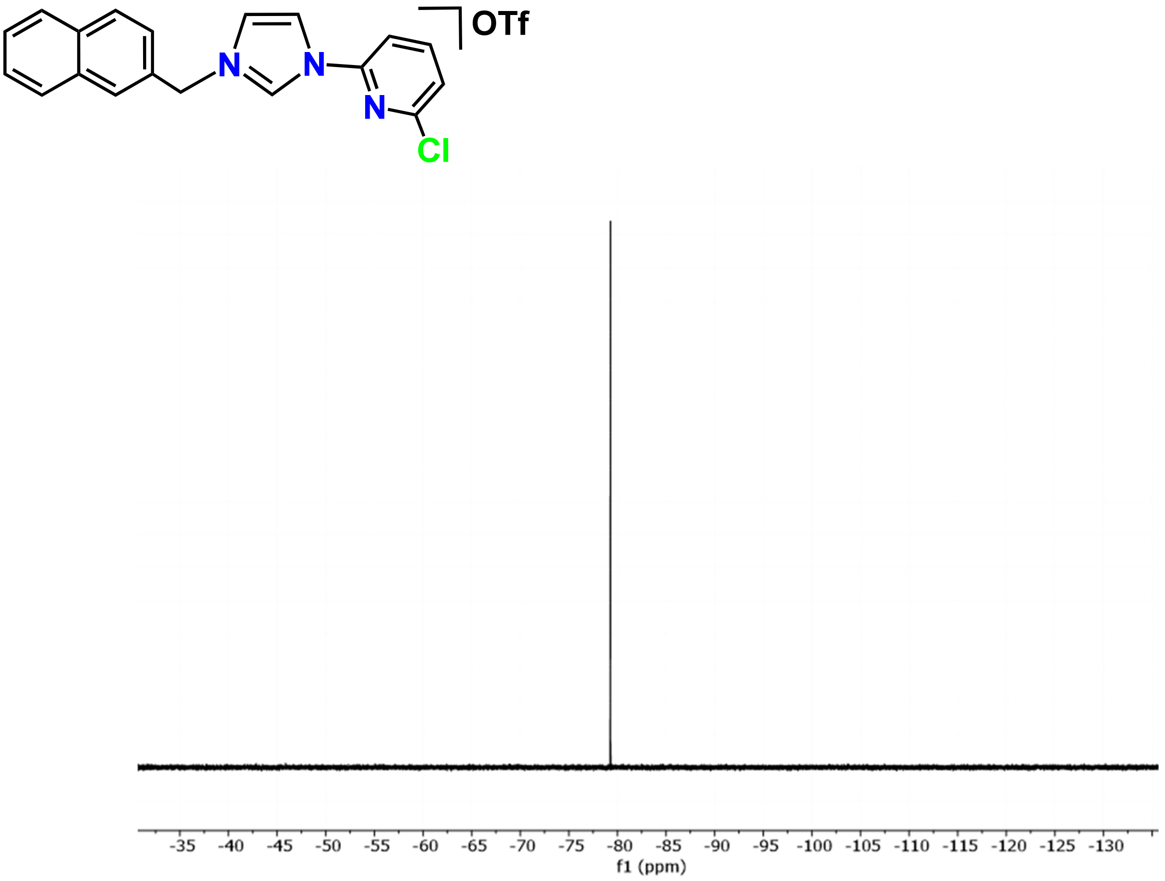

Figure S10. ^{19}F NMR spectrum of **(HL^6)OTf**.

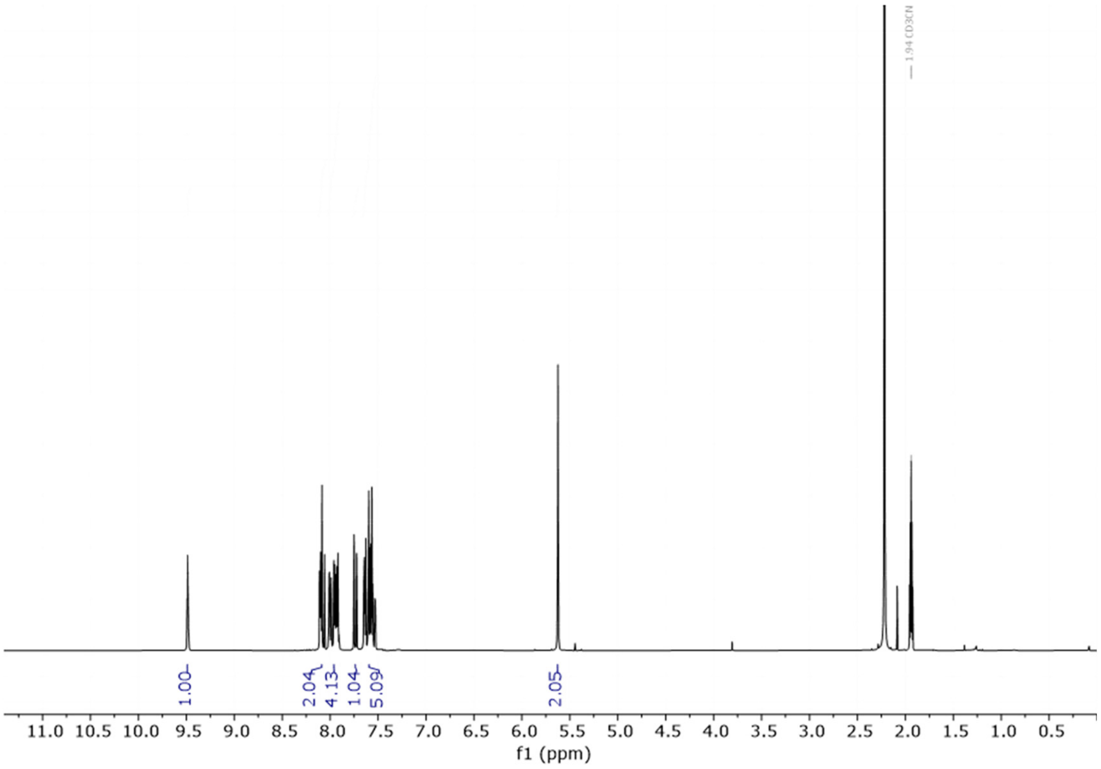

Figure S11. ^{1}H NMR spectrum of **(HL^{6})OTf**.

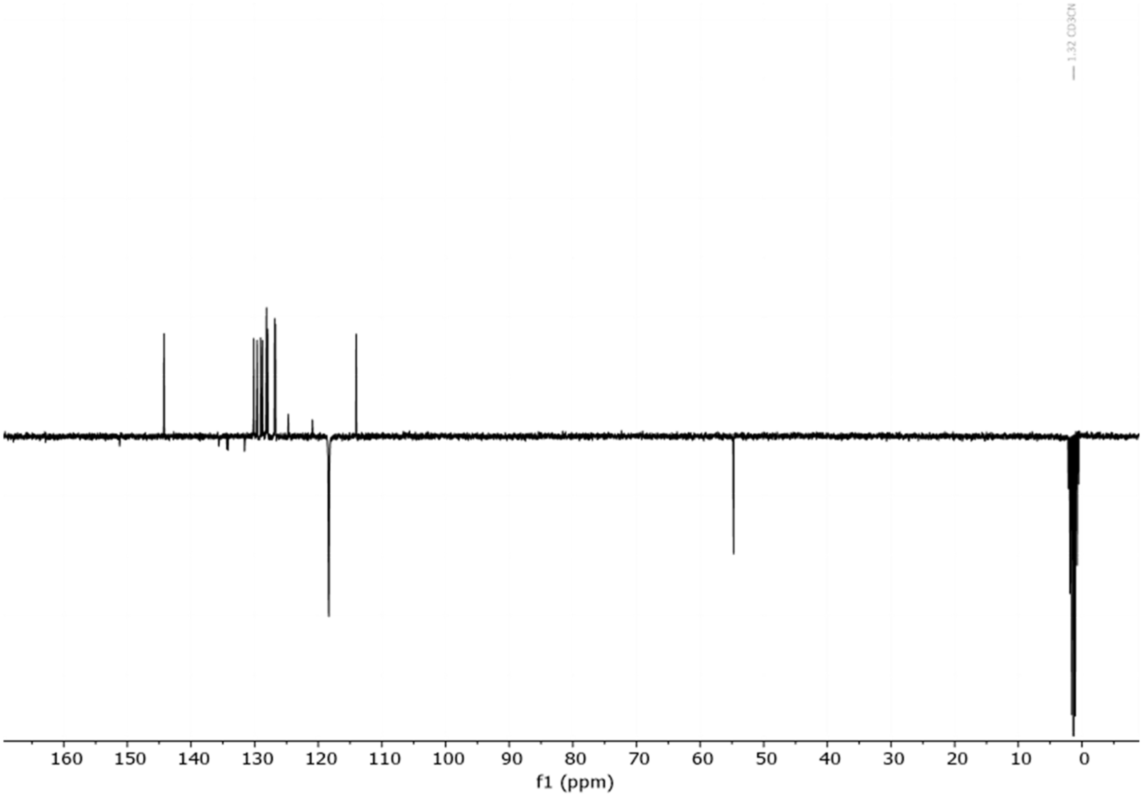

Figure S12. $^{13}C\{^{1}H\}$-APT NMR spectrum of **(HL^{6})OTf**.

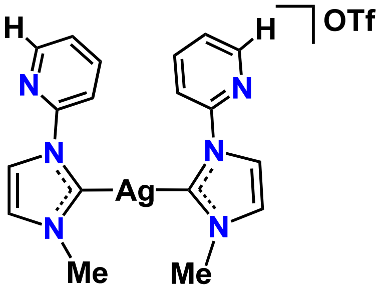

[Ag(Me-Im-Py)$_2$]OTf, Ag1

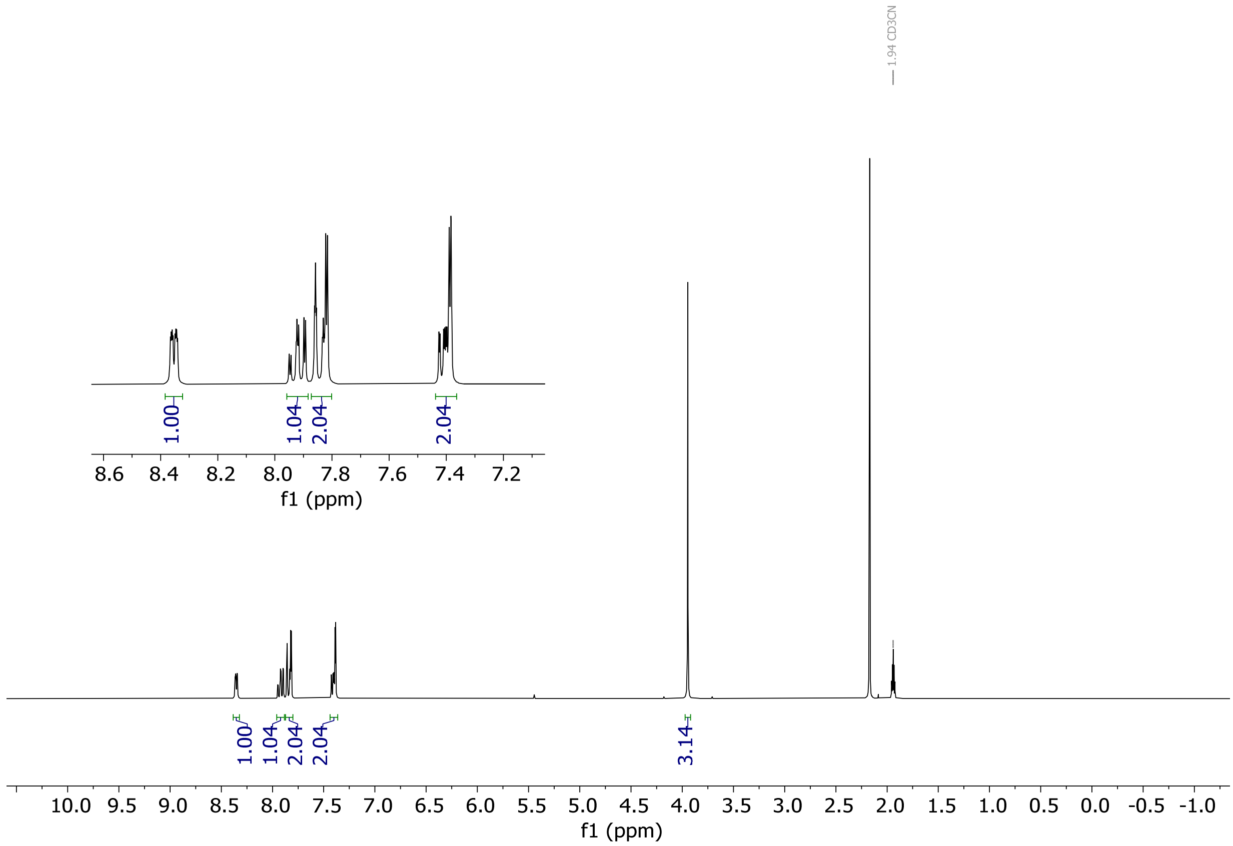

Figure S13. ^{1}H NMR spectrum of **Ag1**.

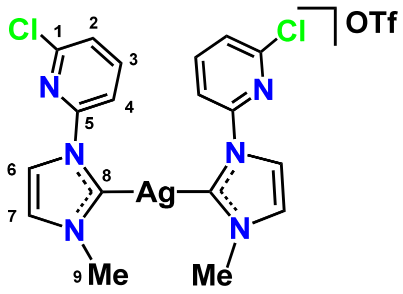

[Ag(Me-Im-2ClPy)₂]OTf, Ag2

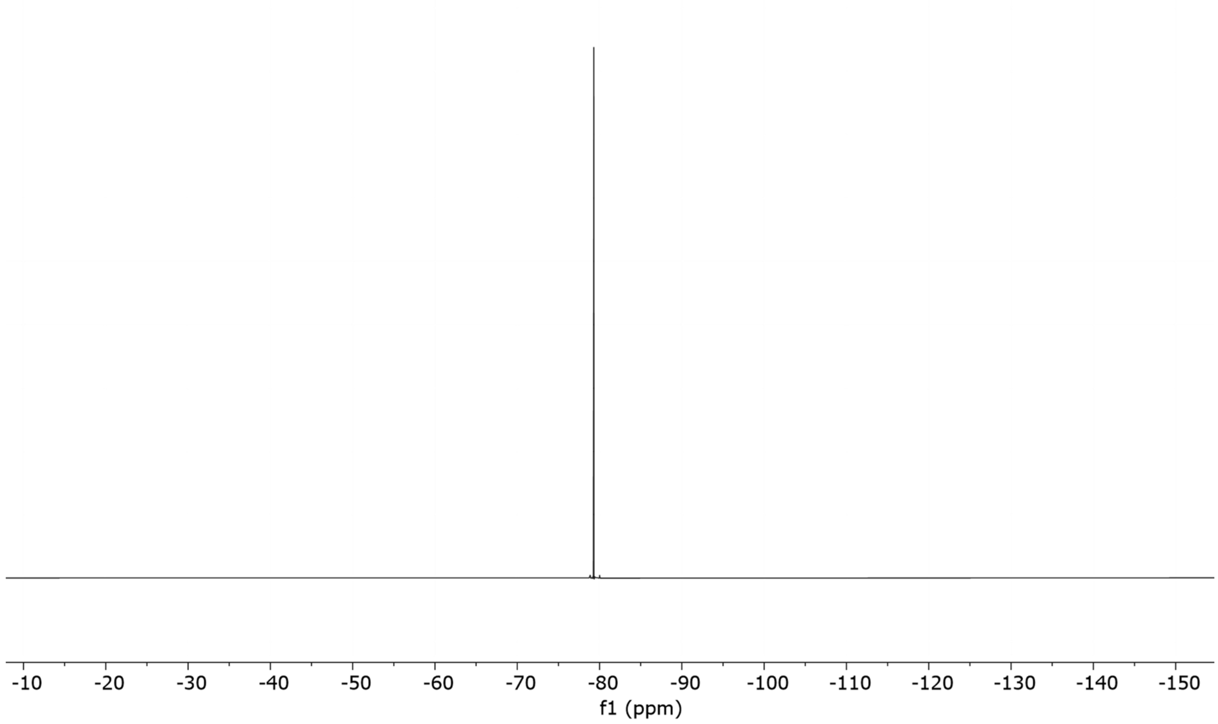

Figure S14. ^{19}F NMR spectrum of **Ag2**.

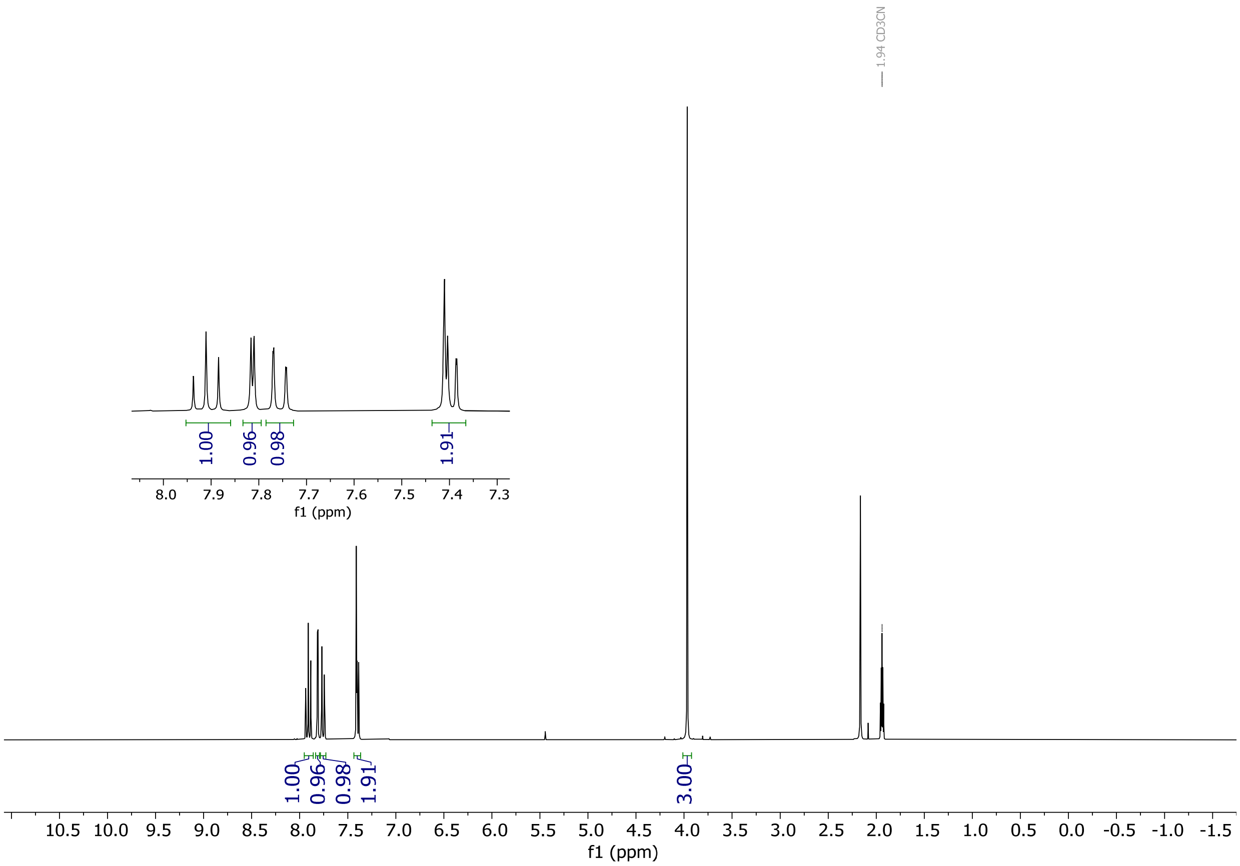

Figure S15. ^{1}H NMR spectrum of **Ag2**.

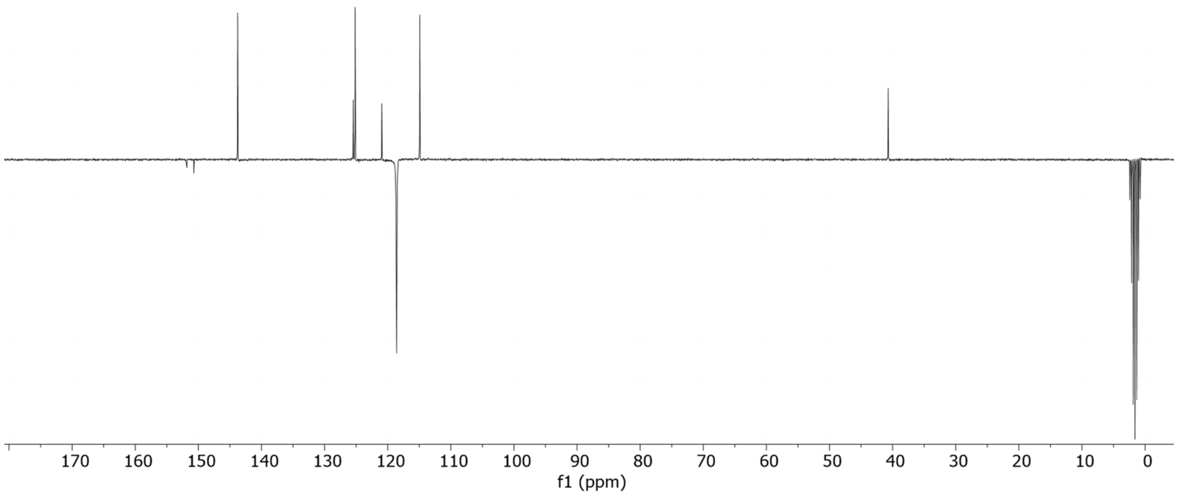

Figure S16. $^{13}C\{^1H\}$-APT NMR spectrum of **Ag2**.

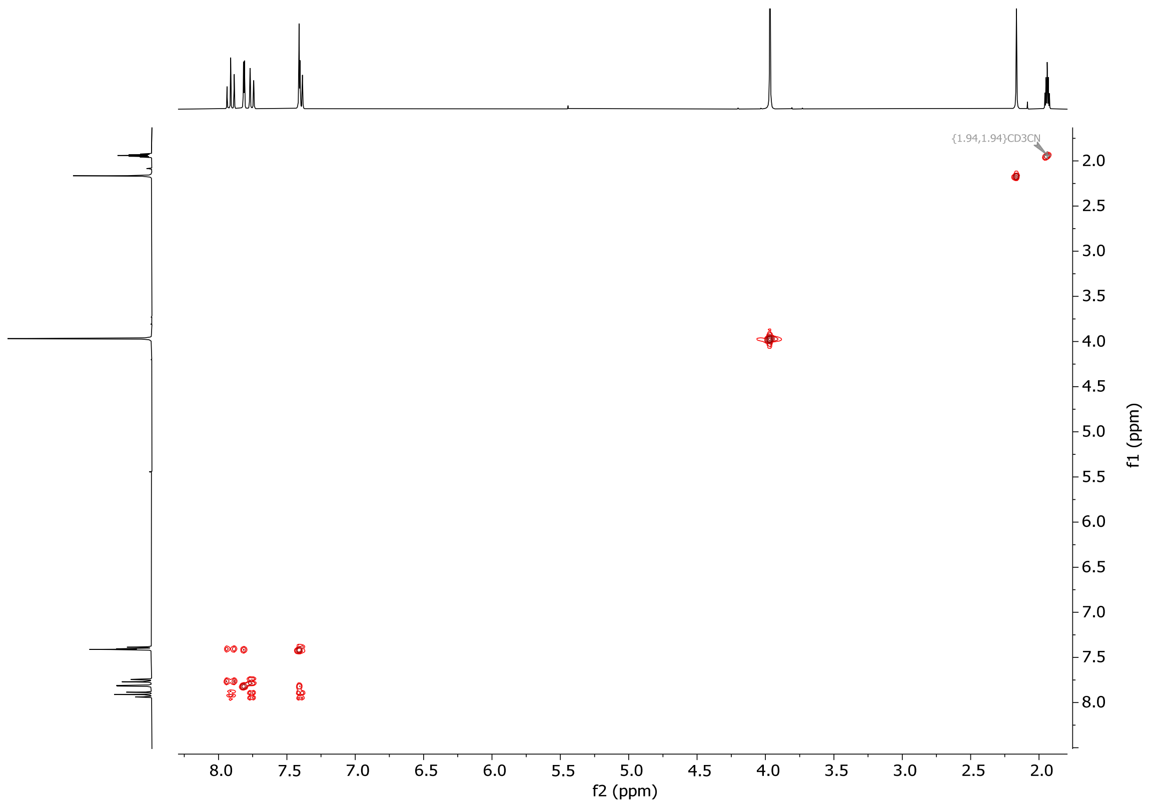

Figure S17. COSY ^{1}H-^{1}H NMR spectrum of **Ag2**.

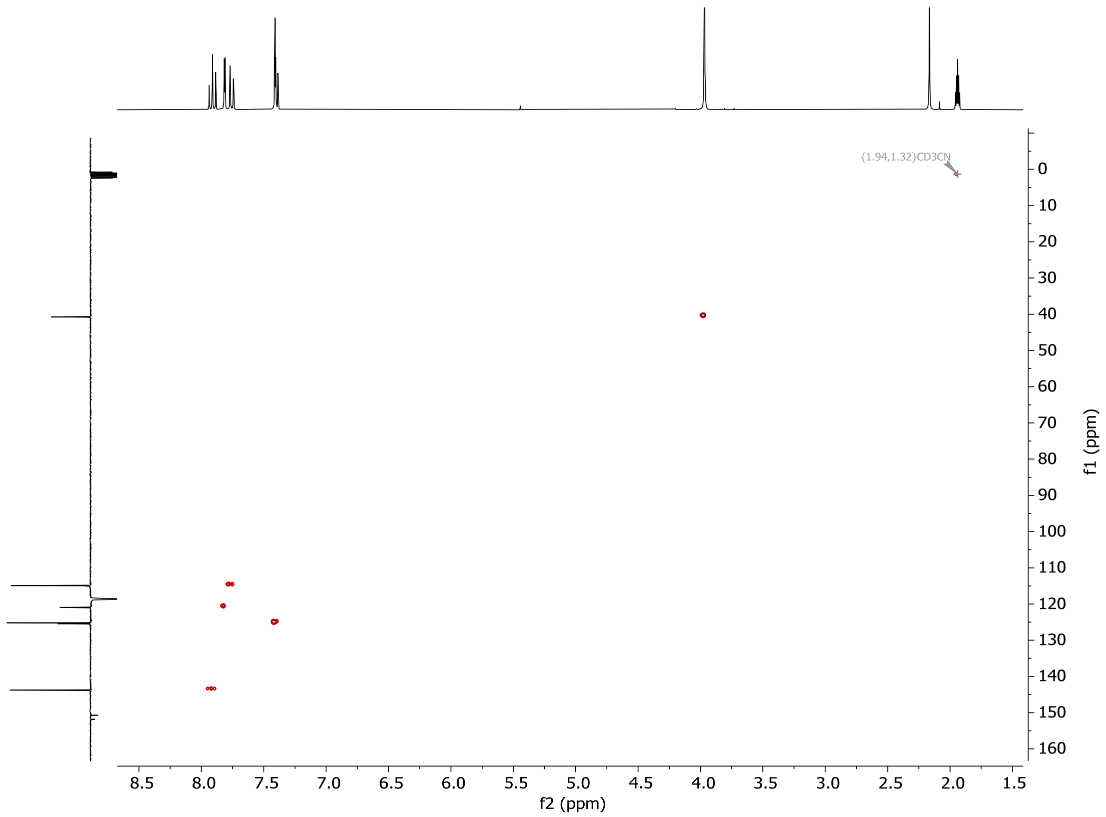

Figure S18. HSQC ^{1}H-^{13}C NMR spectrum of **Ag2**.

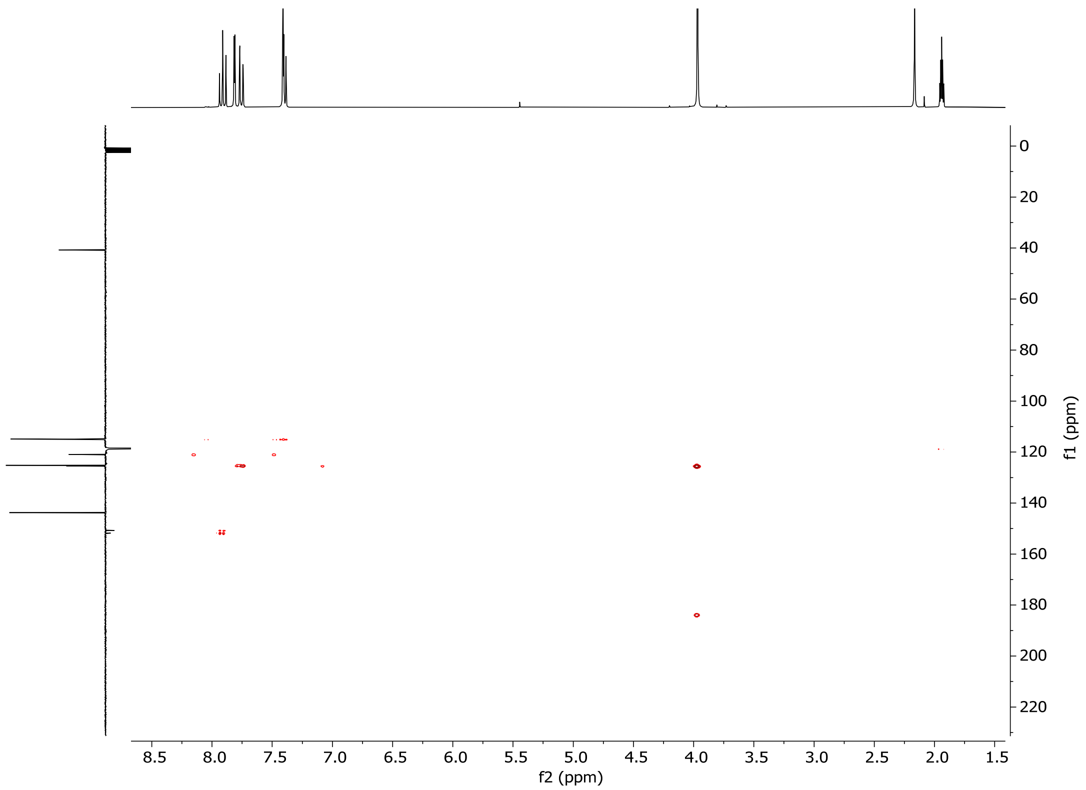

Figure S19. HMBC ^{1}H-^{13}C NMR spectrum of **Ag2**.

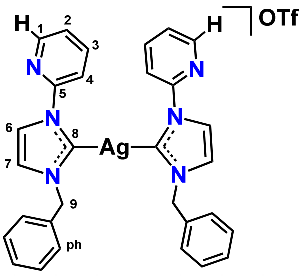

[Ag(Bz-Im-Py)₂]OTf, Ag3

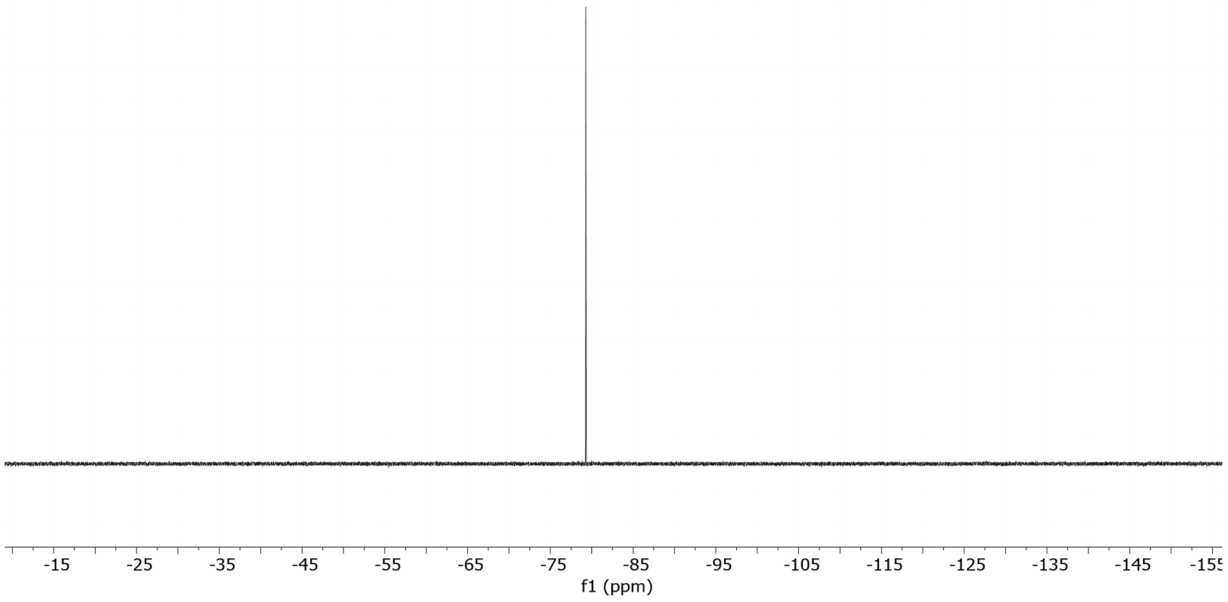

Figure S20. ^{19}F NMR spectrum of **Ag3.**

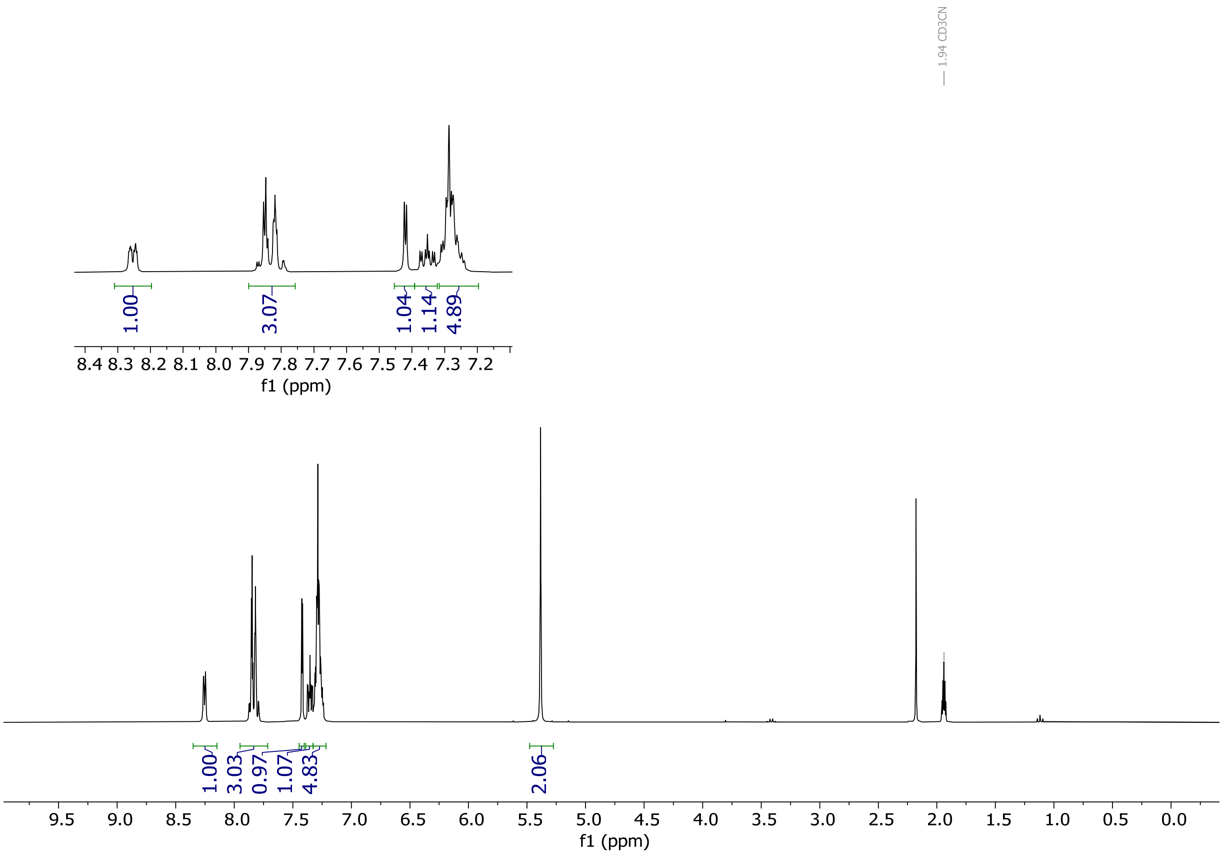

Figure S21. ^{1}H NMR spectrum of **Ag3**.

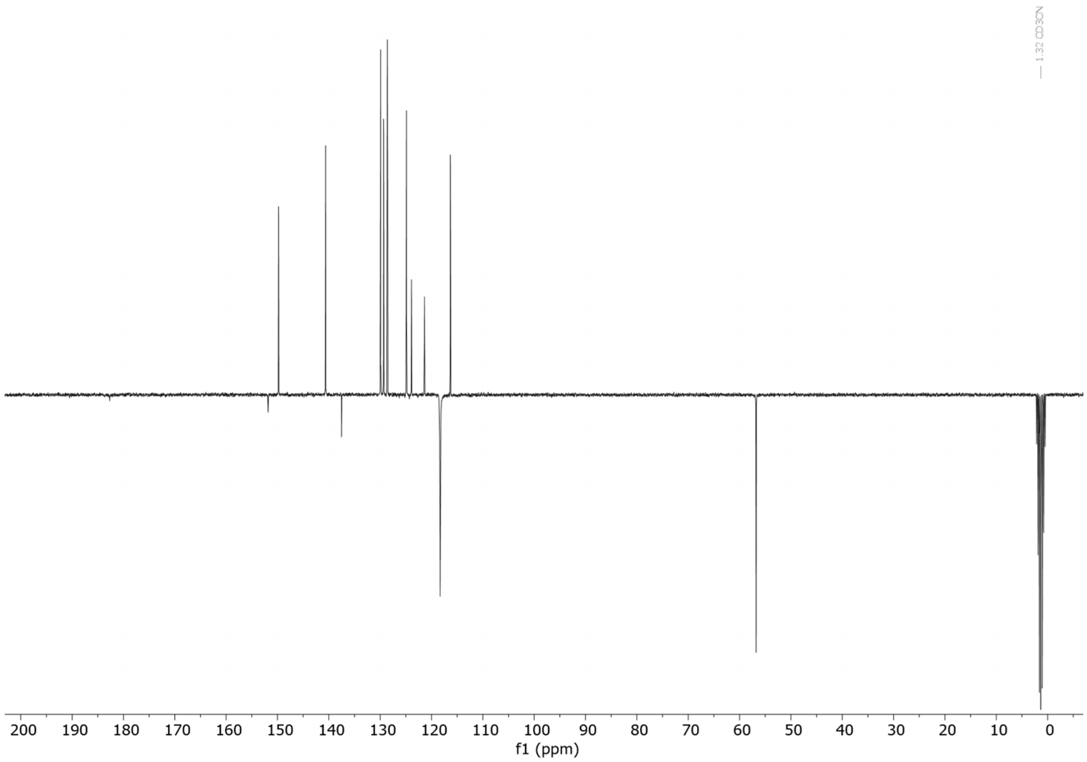

Figure S22. $^{13}C\{^{1}H\}$-APT NMR spectrum of **Ag3**.

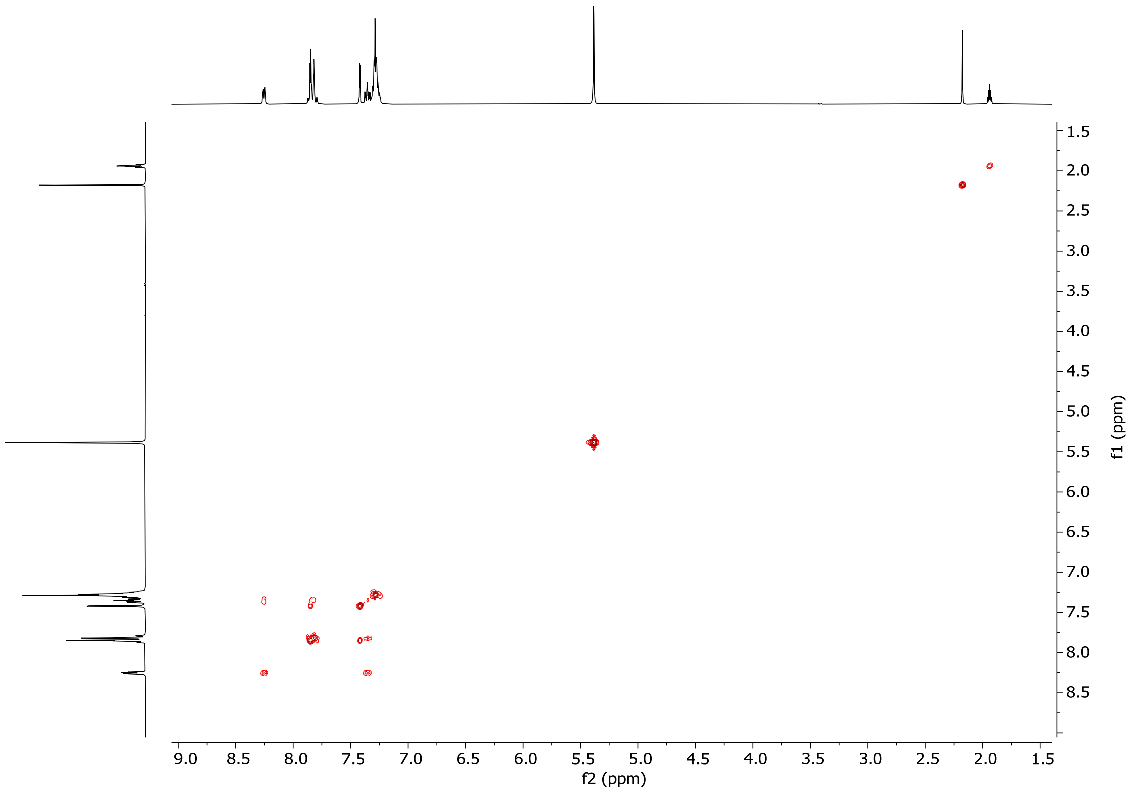

Figure S23. COSY ^{1}H-^{1}H NMR spectrum of **Ag3**.

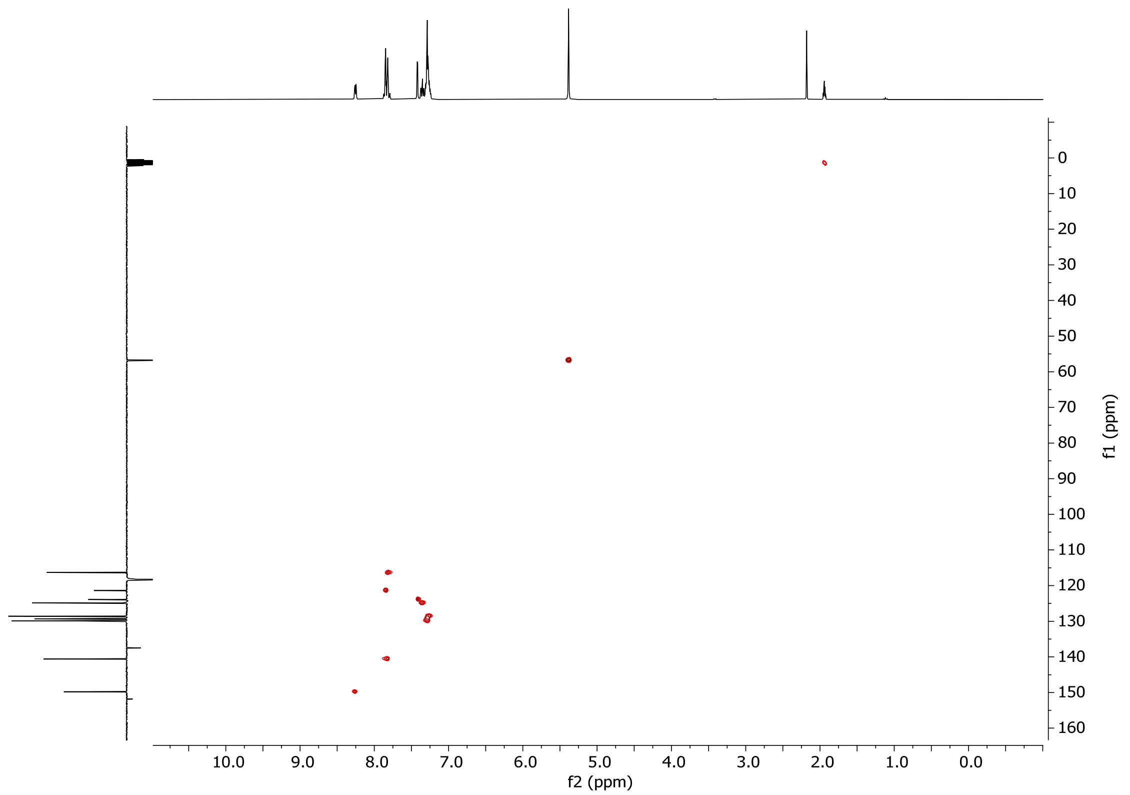

Figure S24. HSQC ^{1}H-^{13}C NMR spectrum of **Ag3**.

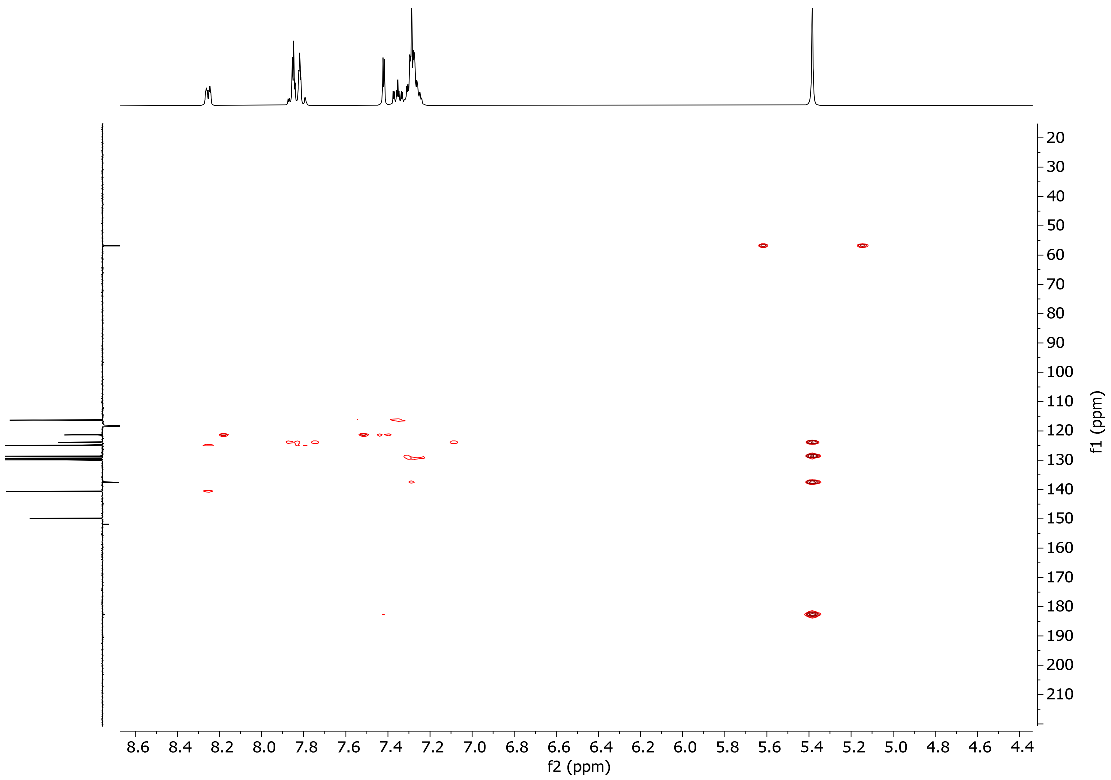

Figure S25. HMBC ^{1}H-^{13}C NMR spectrum of **Ag3**.

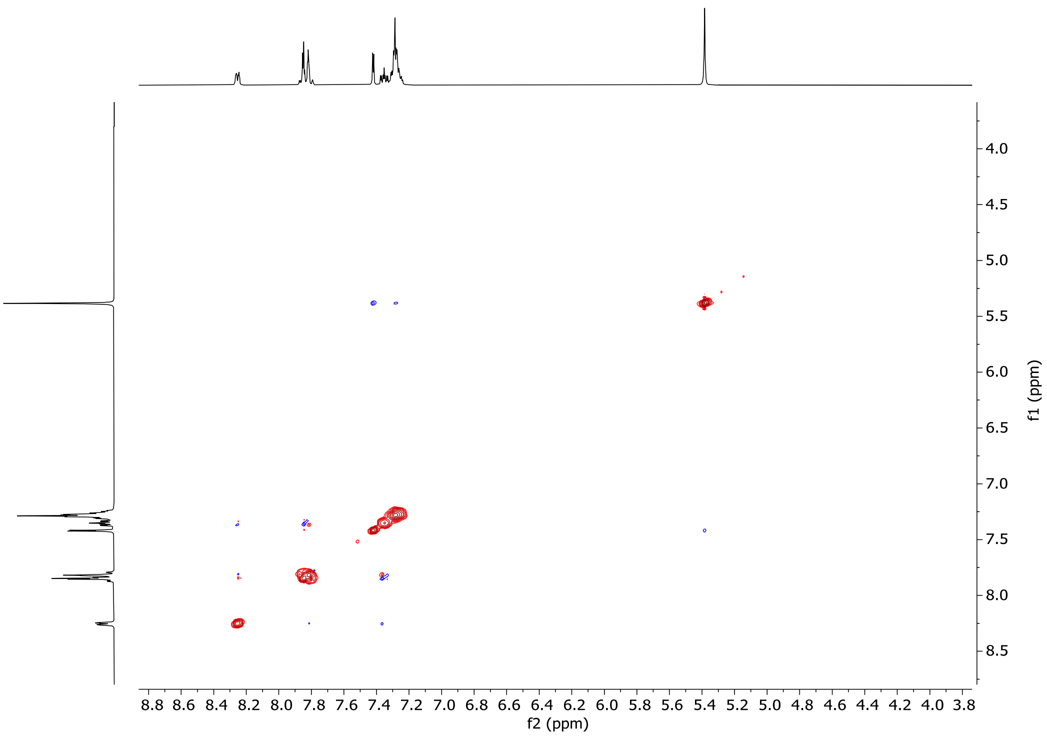

Figure S26. NOESY ^{1}H-^{1}H NMR spectrum of **Ag3**.

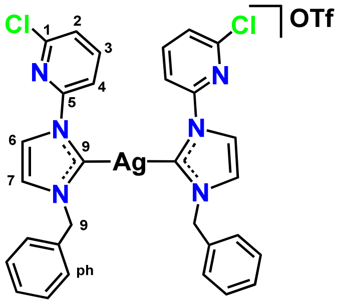

[Ag(Bz-Im-2ClPy)$_2$]OTf, Ag4

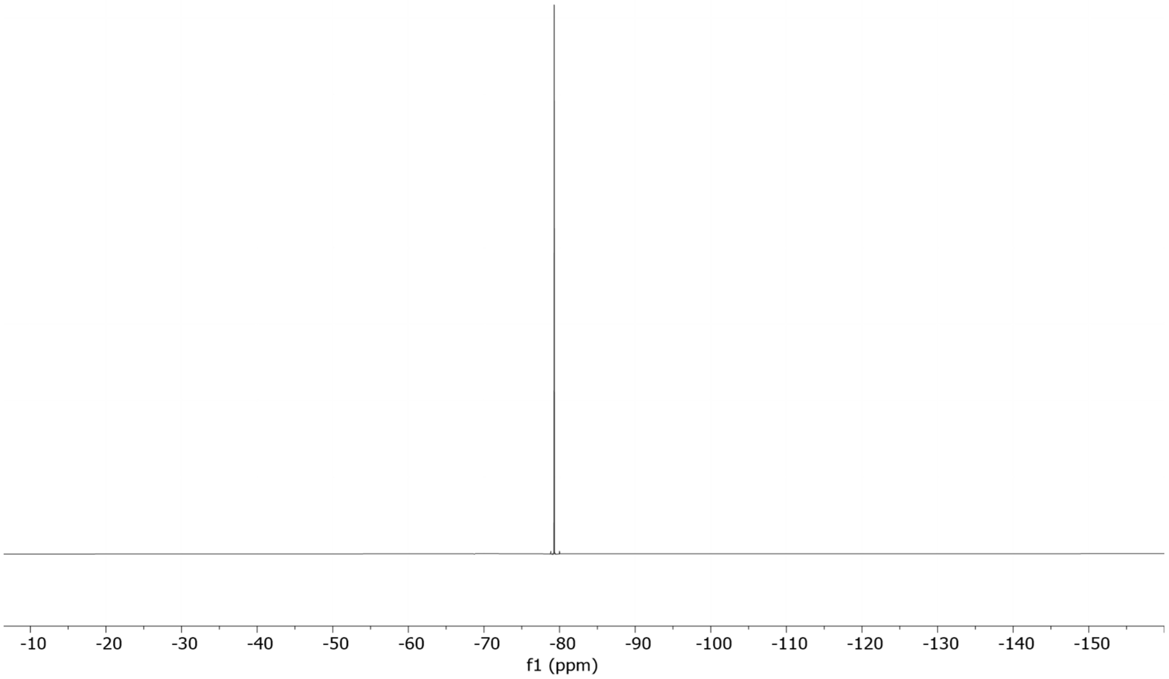

Figure S27. ^{19}F NMR spectrum of **Ag4**.

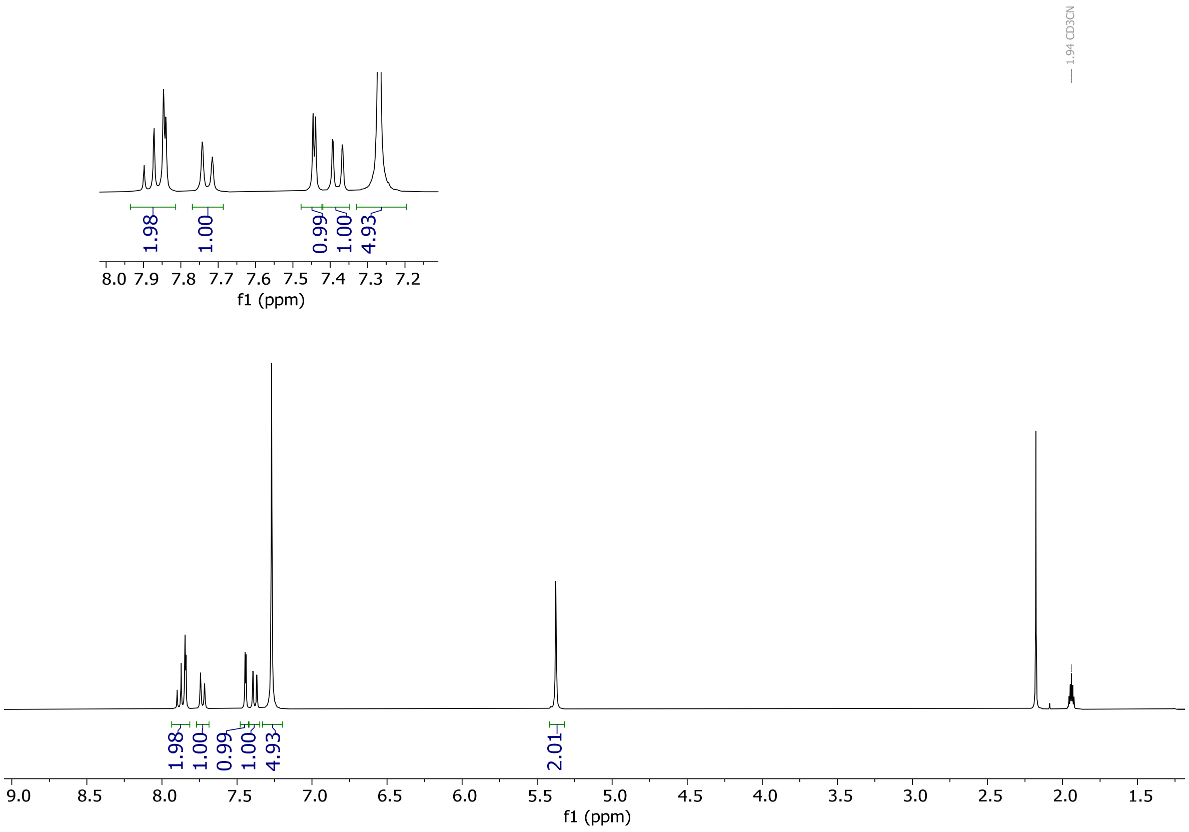

Figure S28. ^{1}H NMR spectrum of **Ag4**.

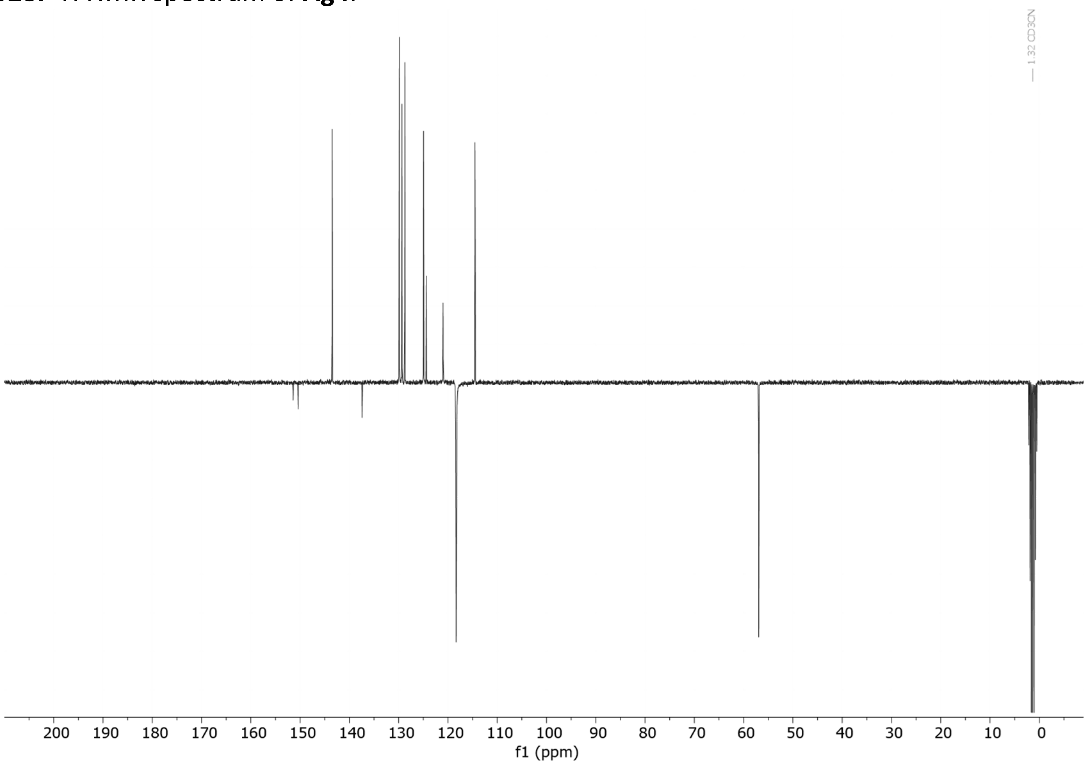

Figure S29. ^{13}C{^{1}H}-APT NMR spectrum of **Ag4**.

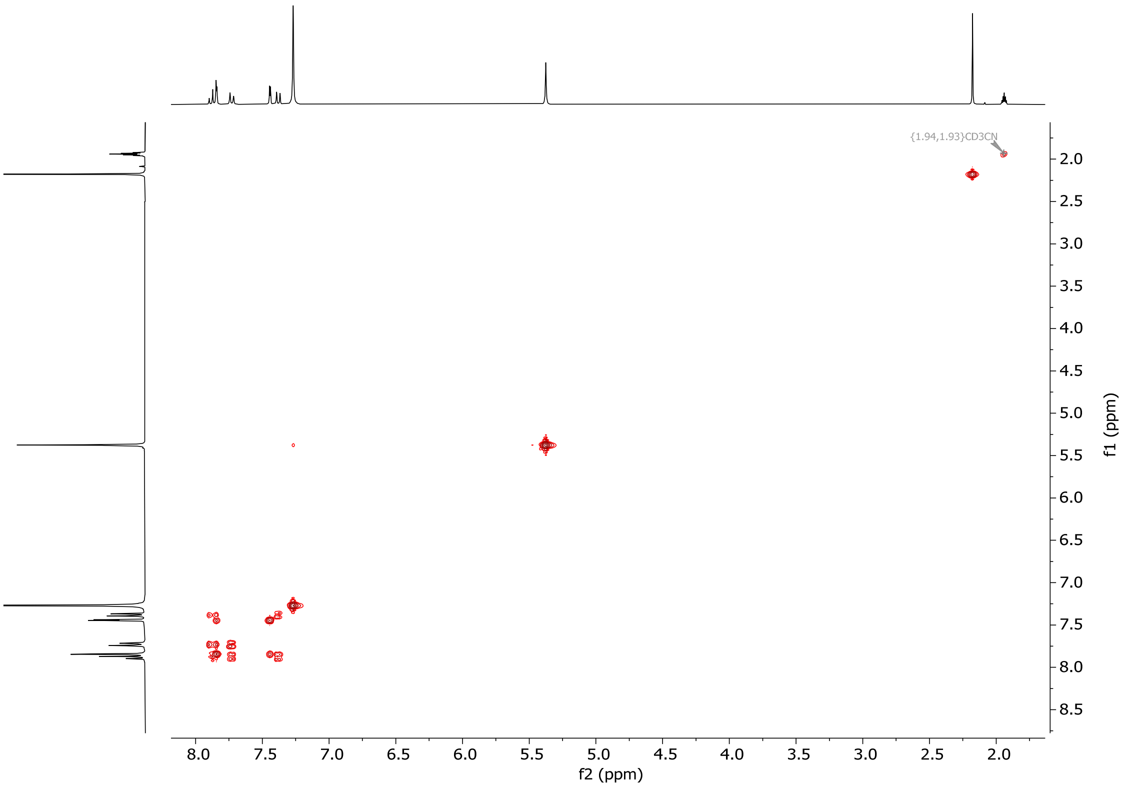

Figure S30. COSY ^{1}H-^{1}H NMR spectrum of **Ag4**.

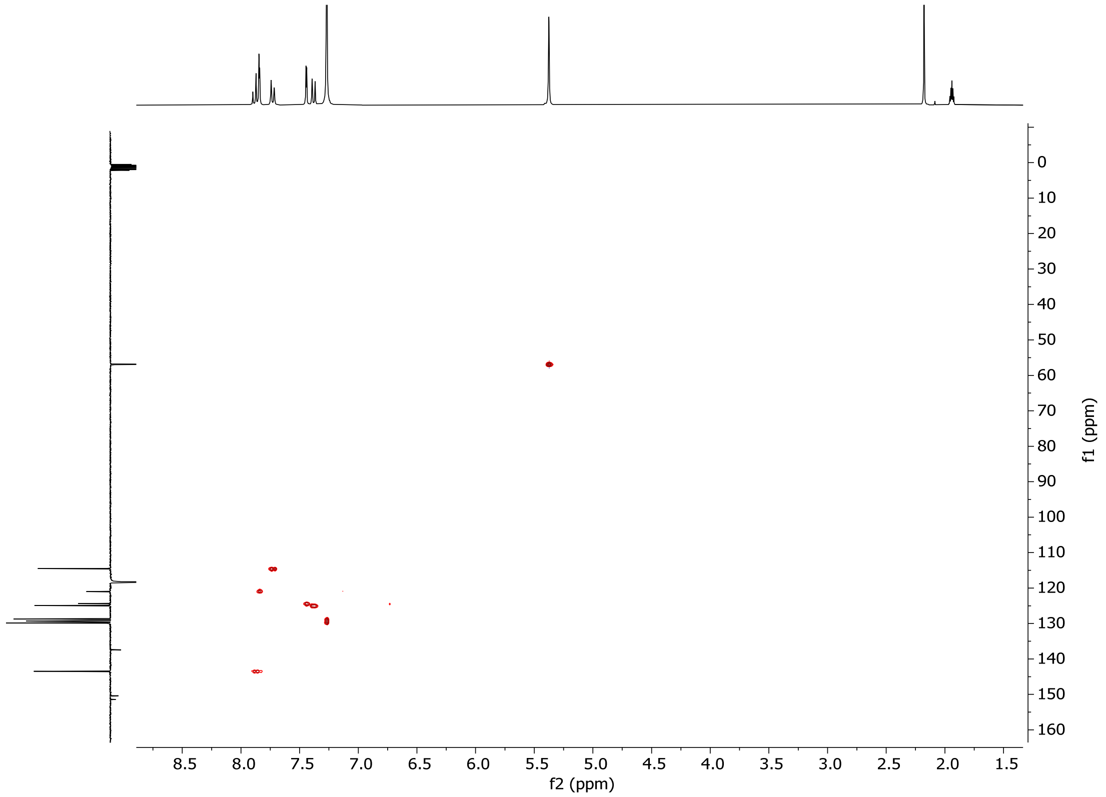

Figure S31. HSQC ^{1}H-^{13}C NMR spectrum of **Ag4**.

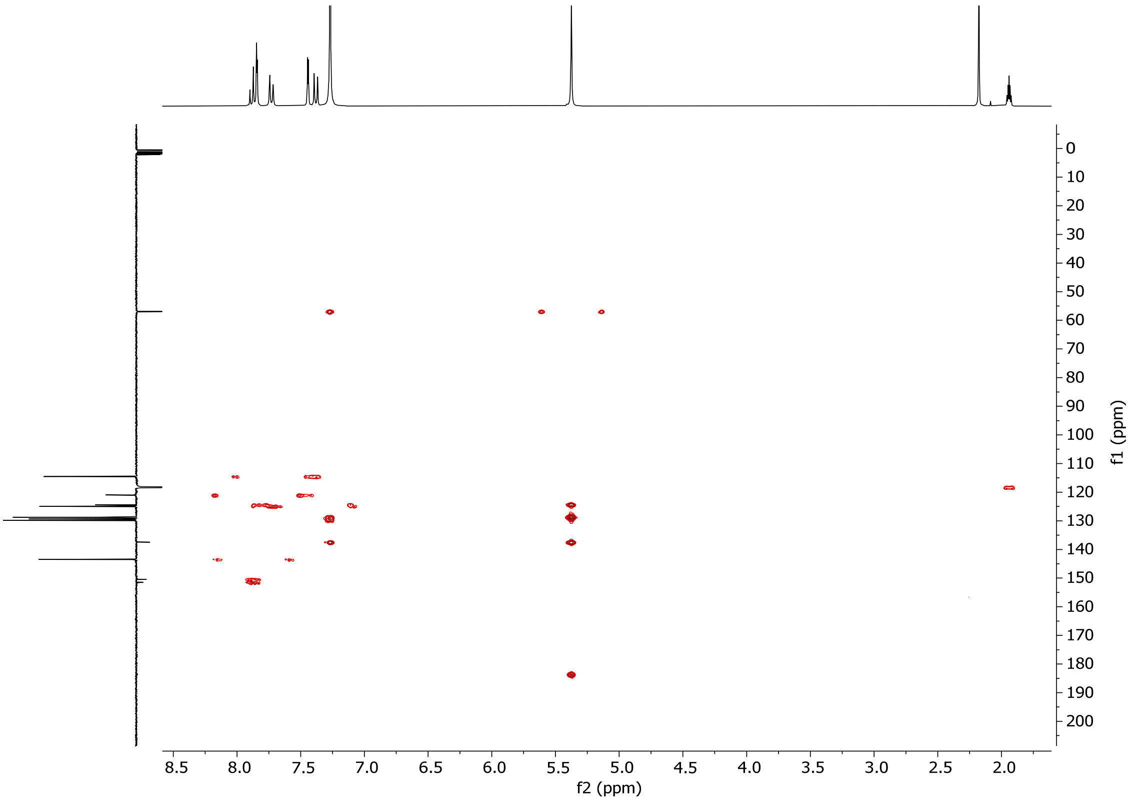

Figure S32. HMBC ^{1}H-^{13}C NMR spectrum of **Ag4**.

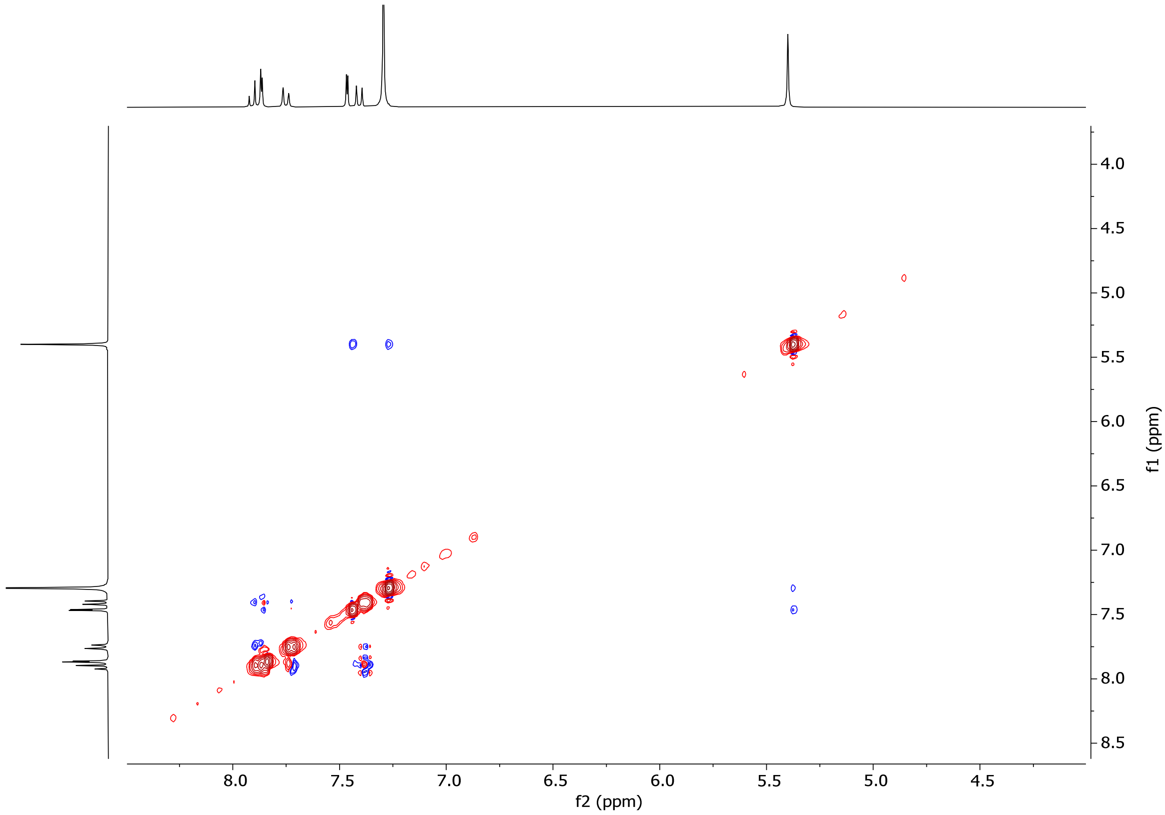

Figure S33. NOESY ^{1}H-^{1}H NMR spectrum of **Ag4**.

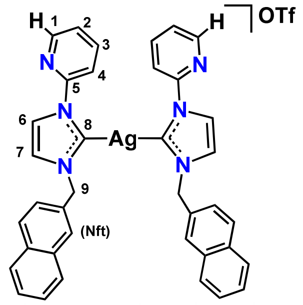

[Ag(NaphCH$_2$-Im-Py)$_2$]OTf, Ag5

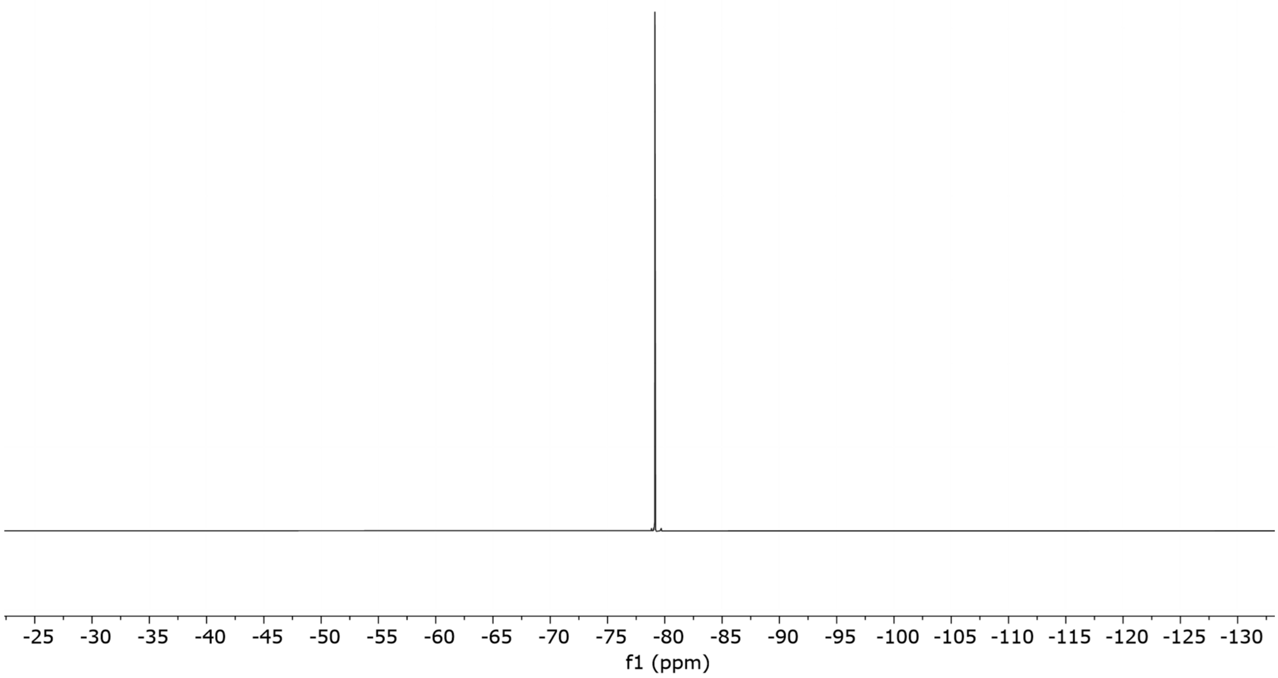

Figure S34. ^{19}F NMR spectrum of **Ag5**.

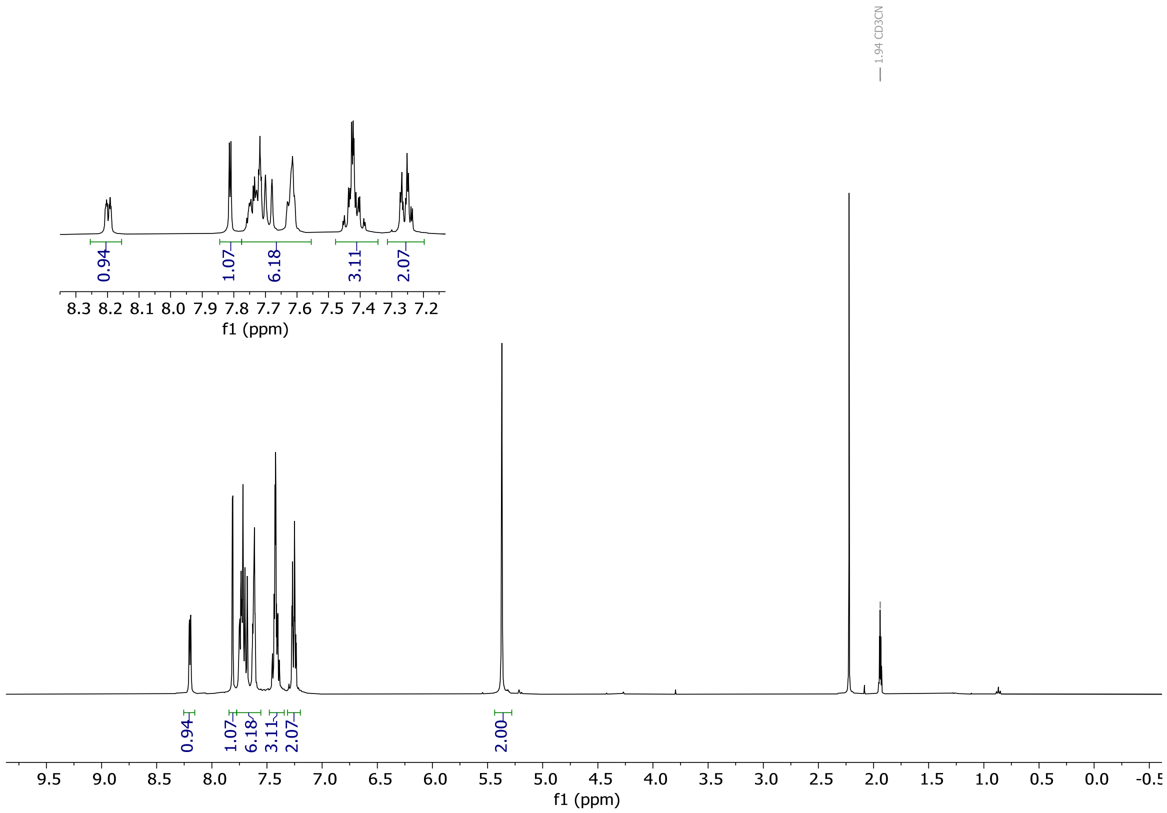

Figure S35. ^{1}H NMR spectrum of **Ag5**.

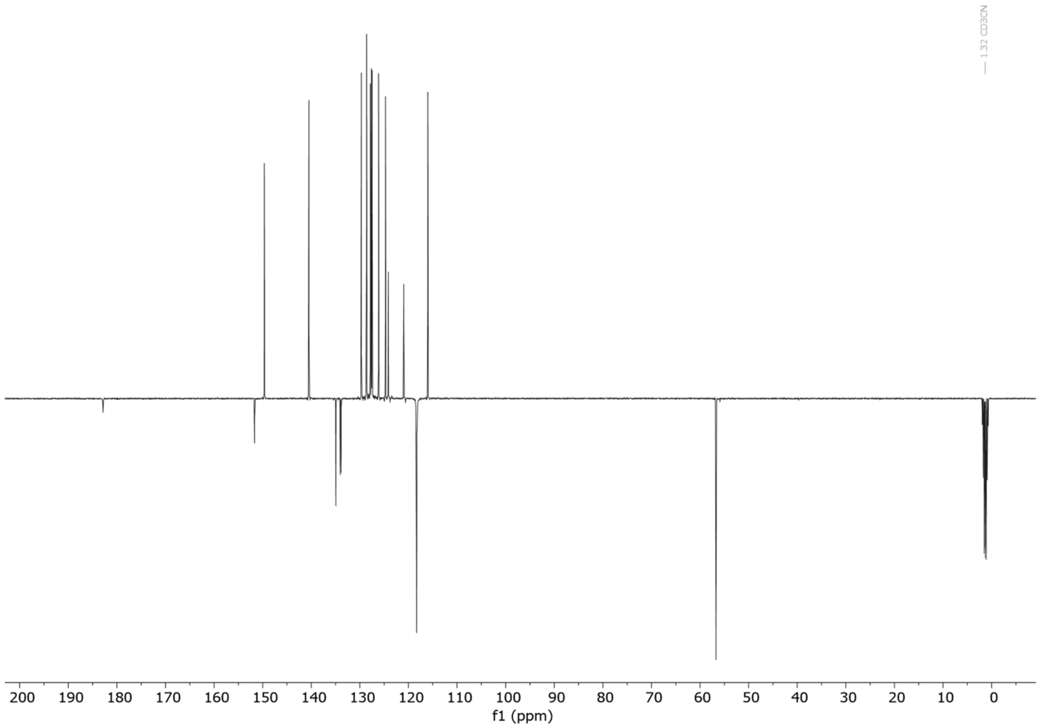

Figure S36. $^{13}C\{^{1}H\}$-APT NMR spectrum of **Ag5**.

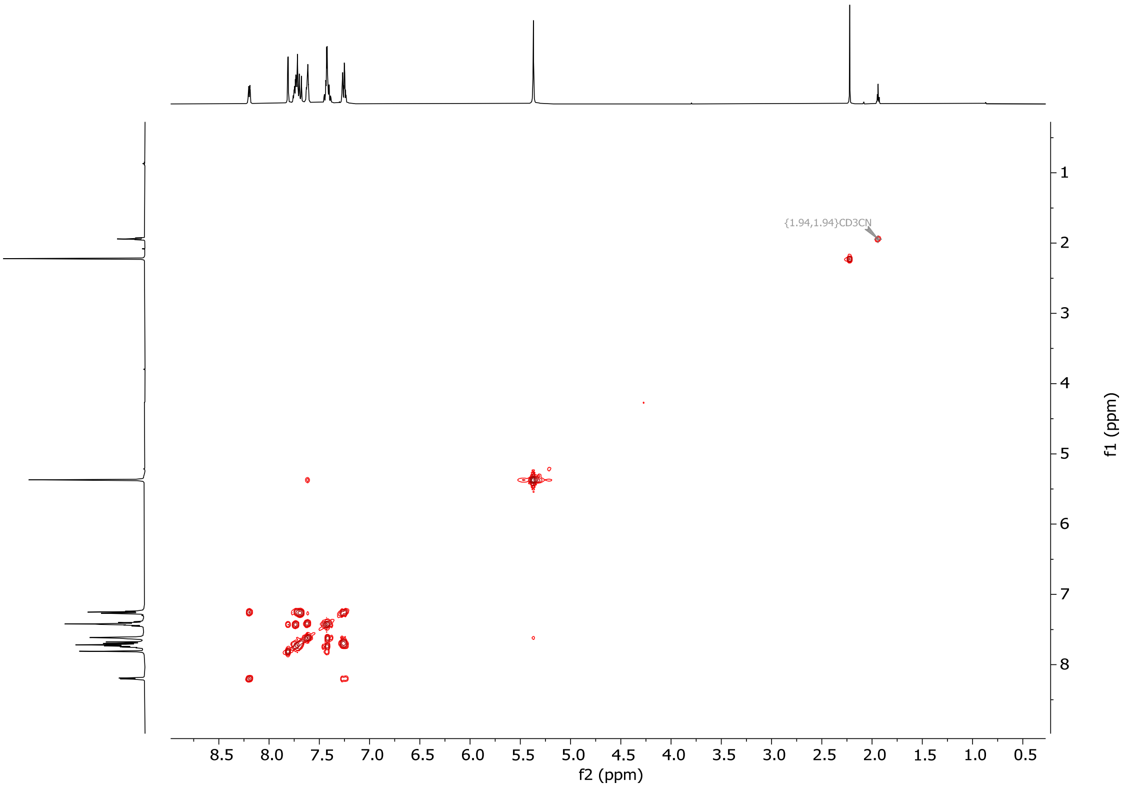

Figure S37. COSY ^1H-^1H NMR spectrum of **Ag5**.

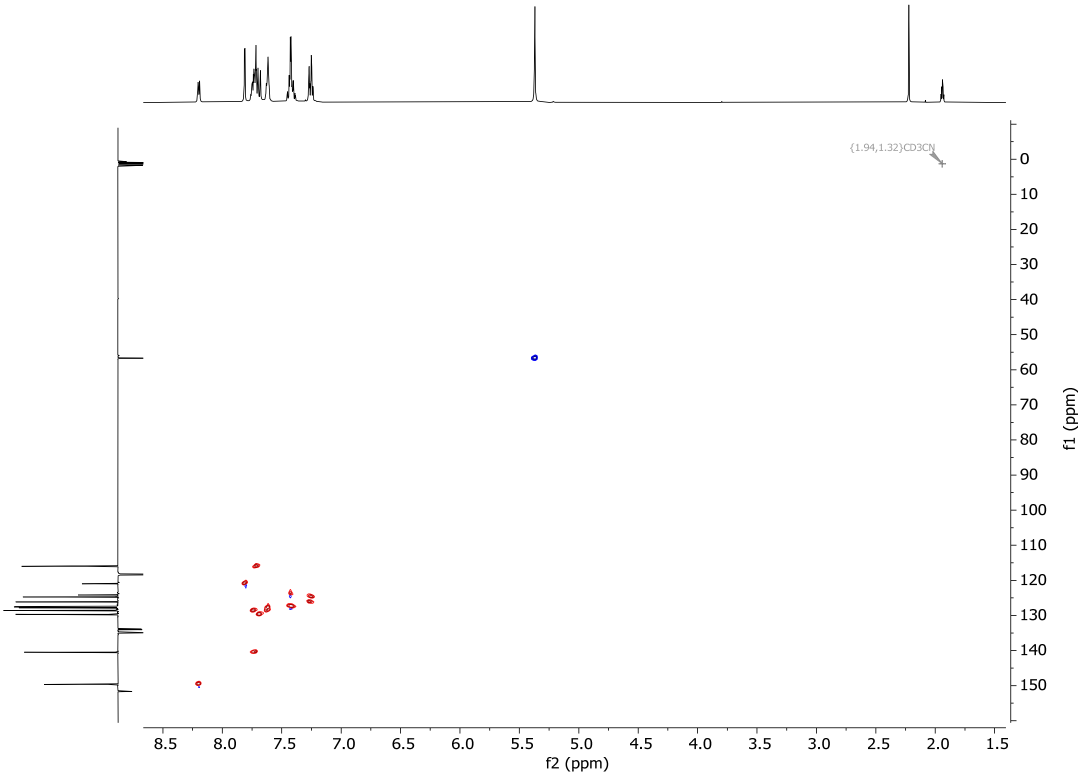

Figure S38. HSQC ^1H-^{13}C NMR spectrum of **Ag5**.

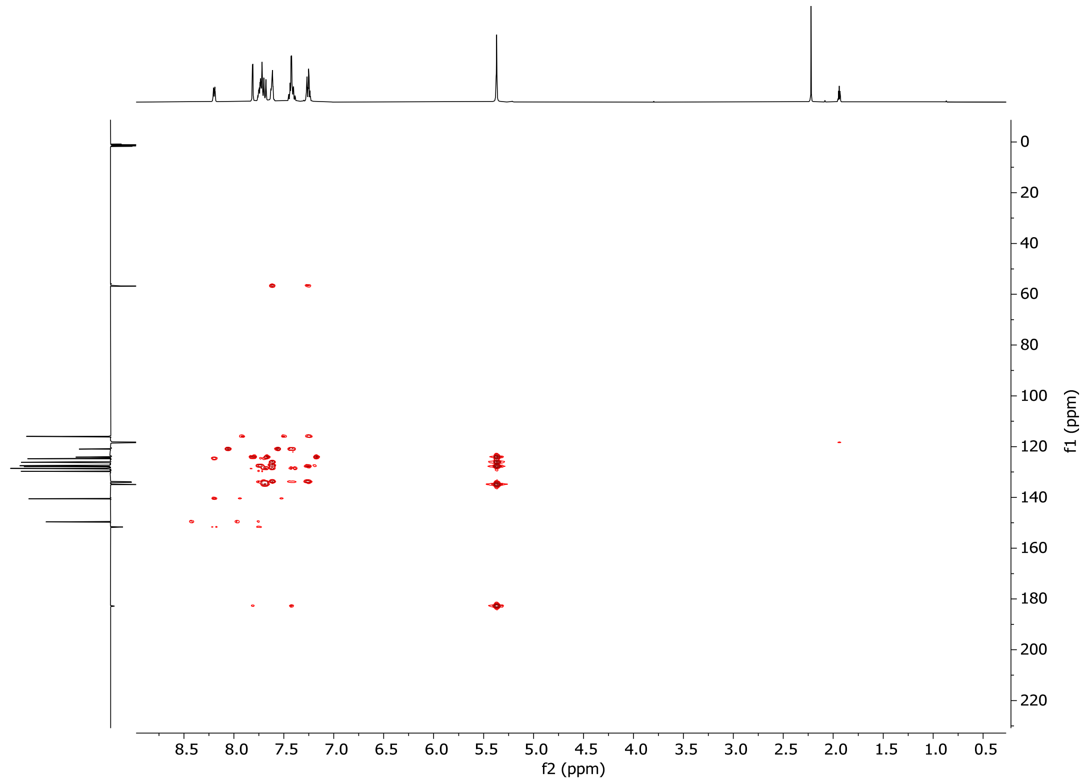

Figure S39. HMBC ^{1}H-^{13}C NMR spectrum of **Ag5**.

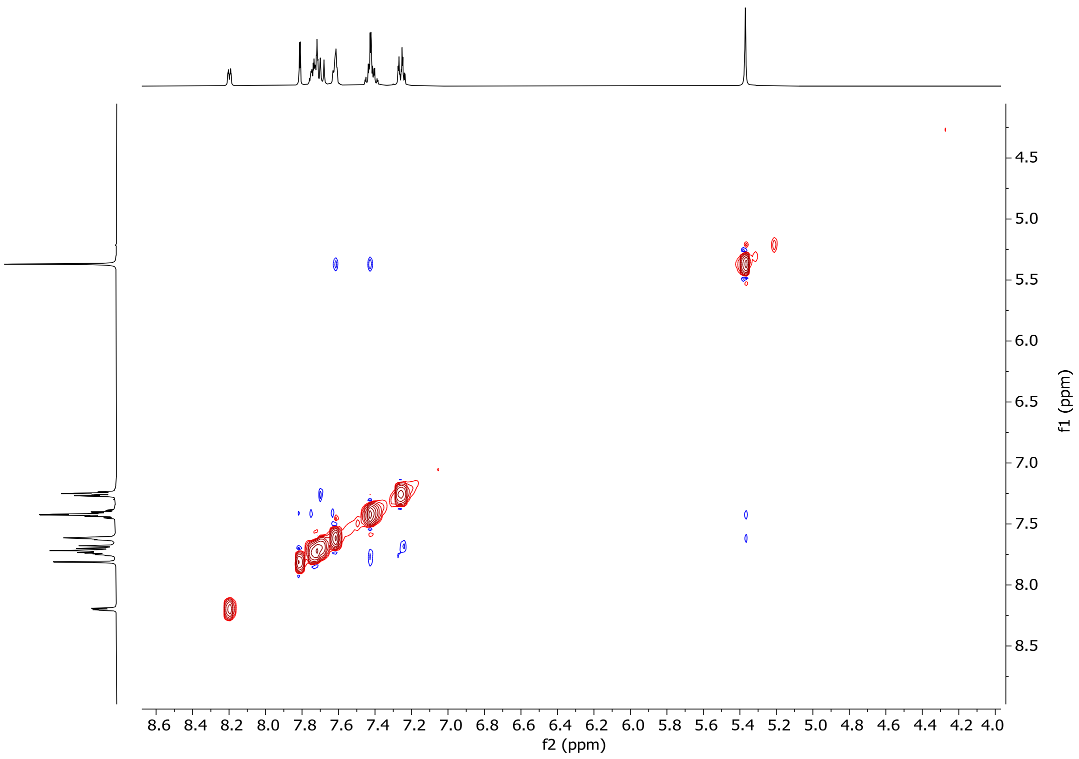

Figure S40. NOESY ^{1}H-^{1}H NMR spectrum of **Ag5**.

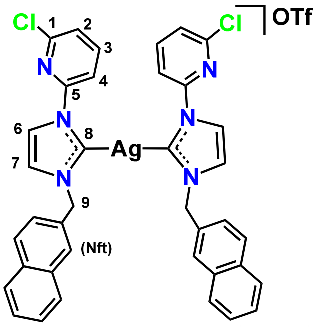

[Ag(NaphCH$_2$-Im-2ClPy)$_2$]OTf, Ag6

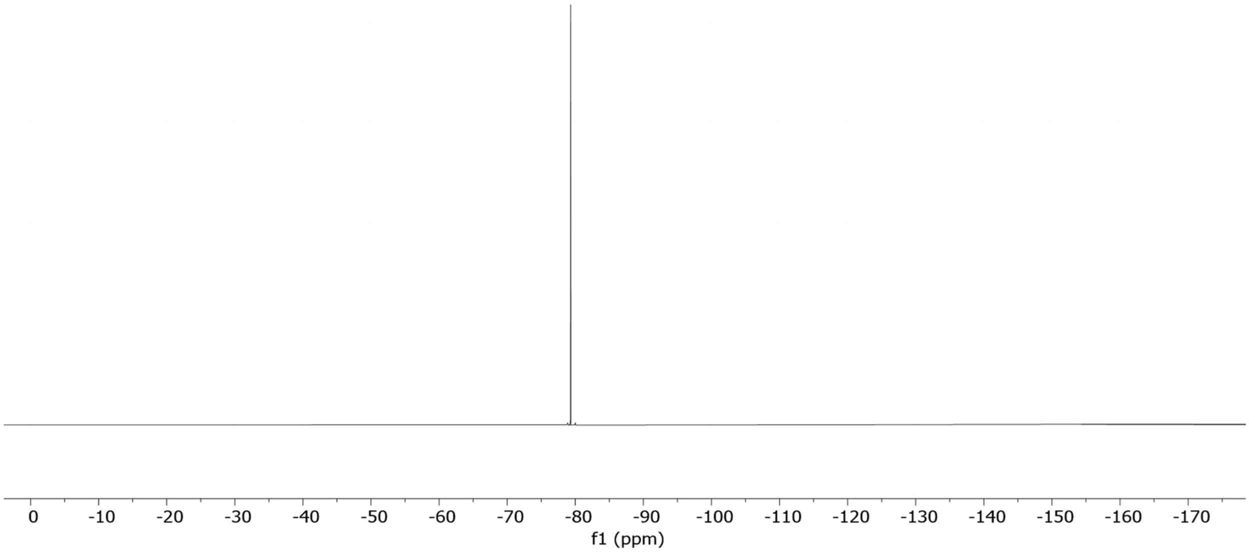

Figure S41. ^{19}F NMR spectrum of **Ag6**.

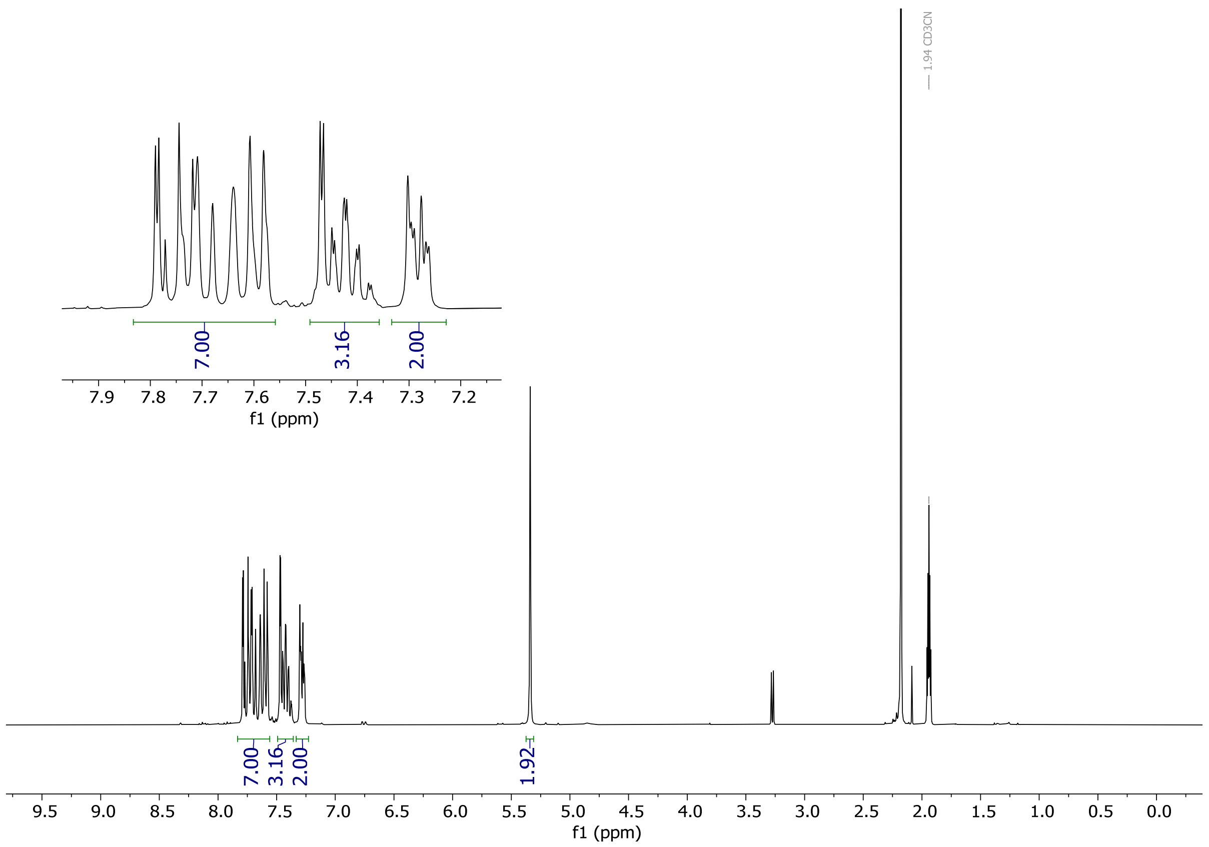

Figure S42. ^{1}H NMR spectrum of **Ag6**.

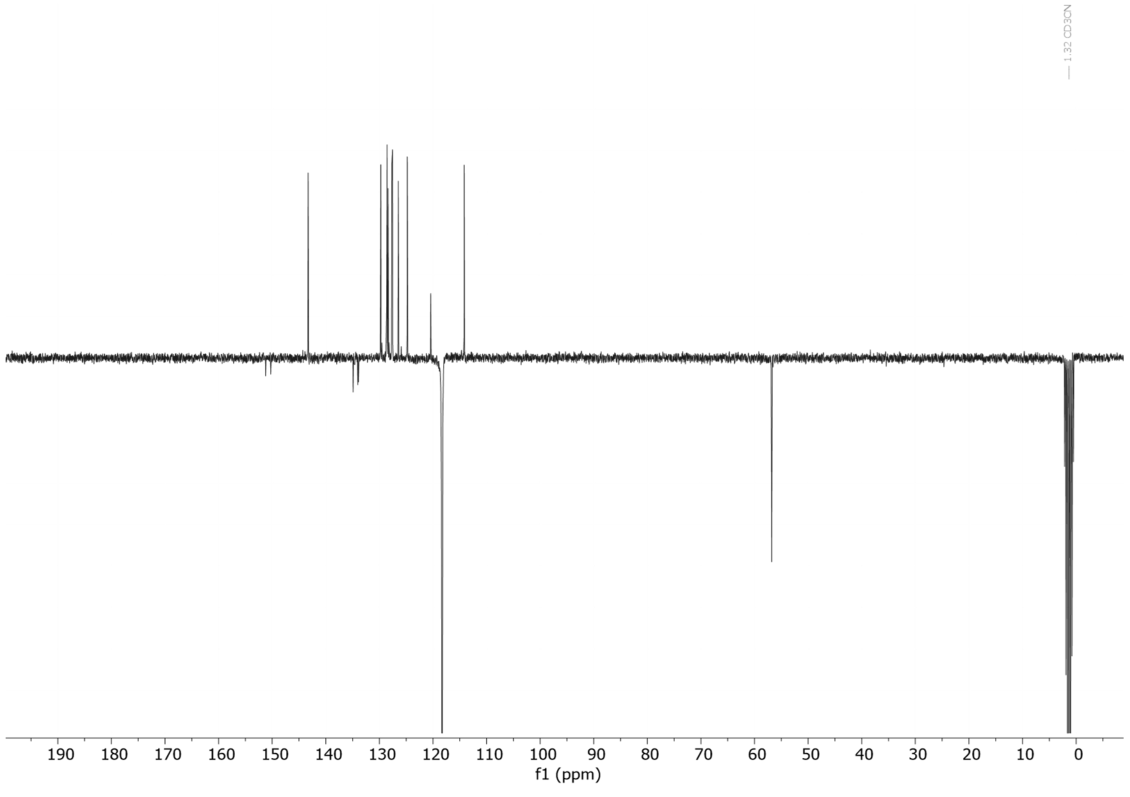

Figure S43. $^{13}C\{^{1}H\}$-APT NMR spectrum of **Ag6**.

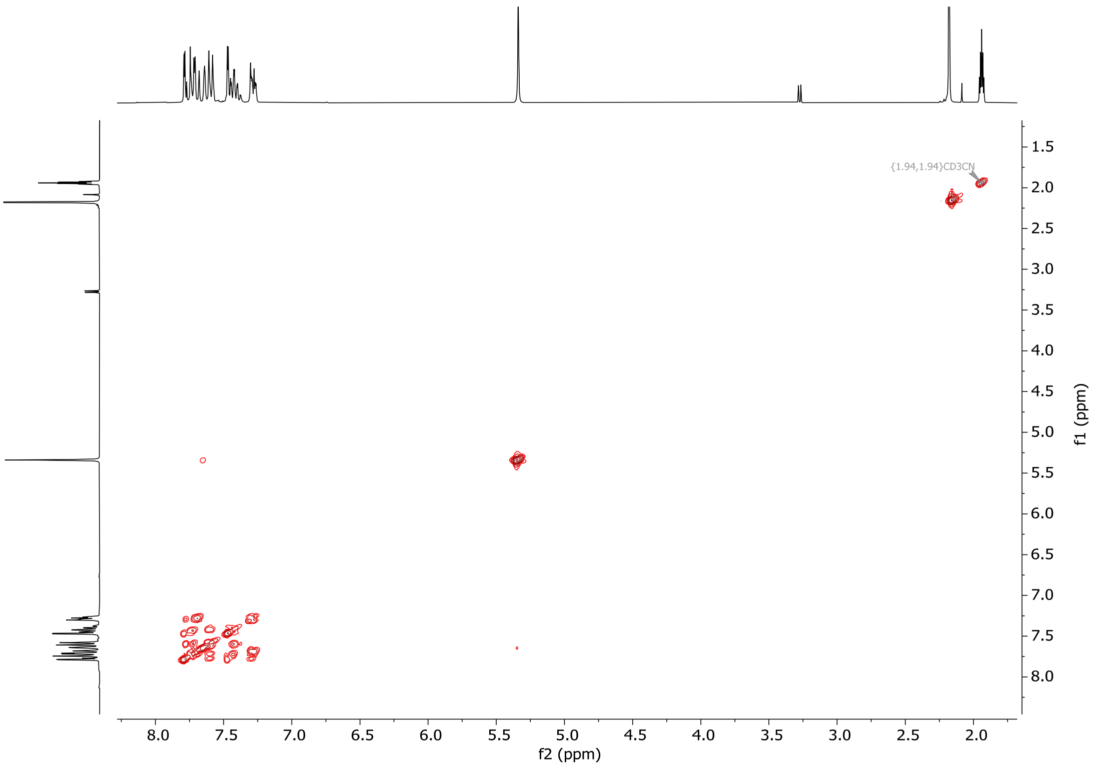

Figure S44. COSY ^{1}H-^{1}H NMR spectrum of **Ag6**.

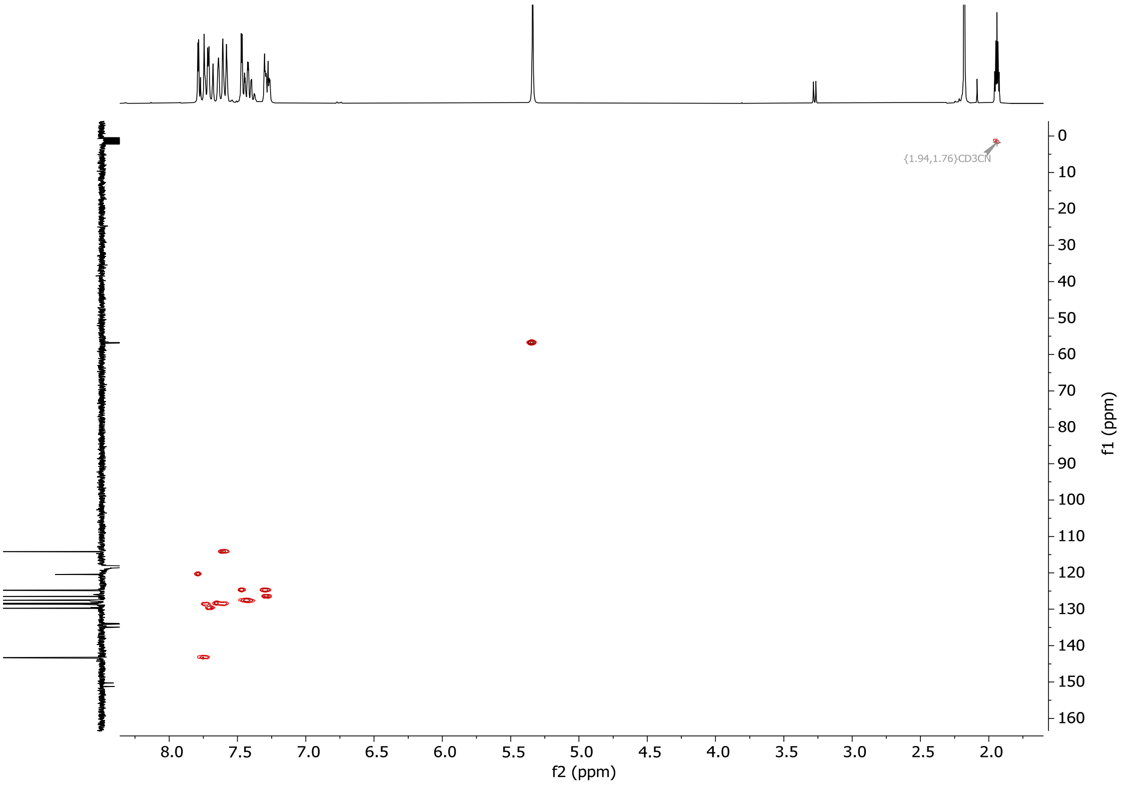

Figure S45. HSQC ^{1}H-^{13}C NMR spectrum of **Ag6**.

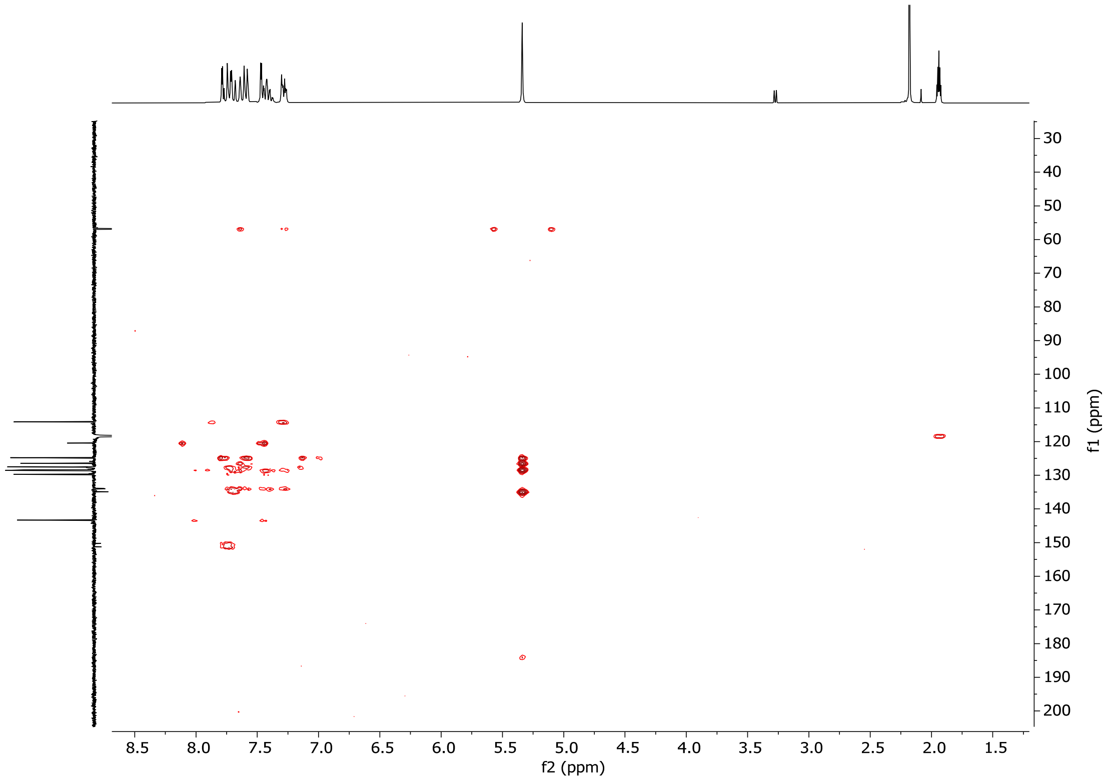

Figure S46. HMBC ^1H-^{13}C NMR spectrum of **Ag6**.

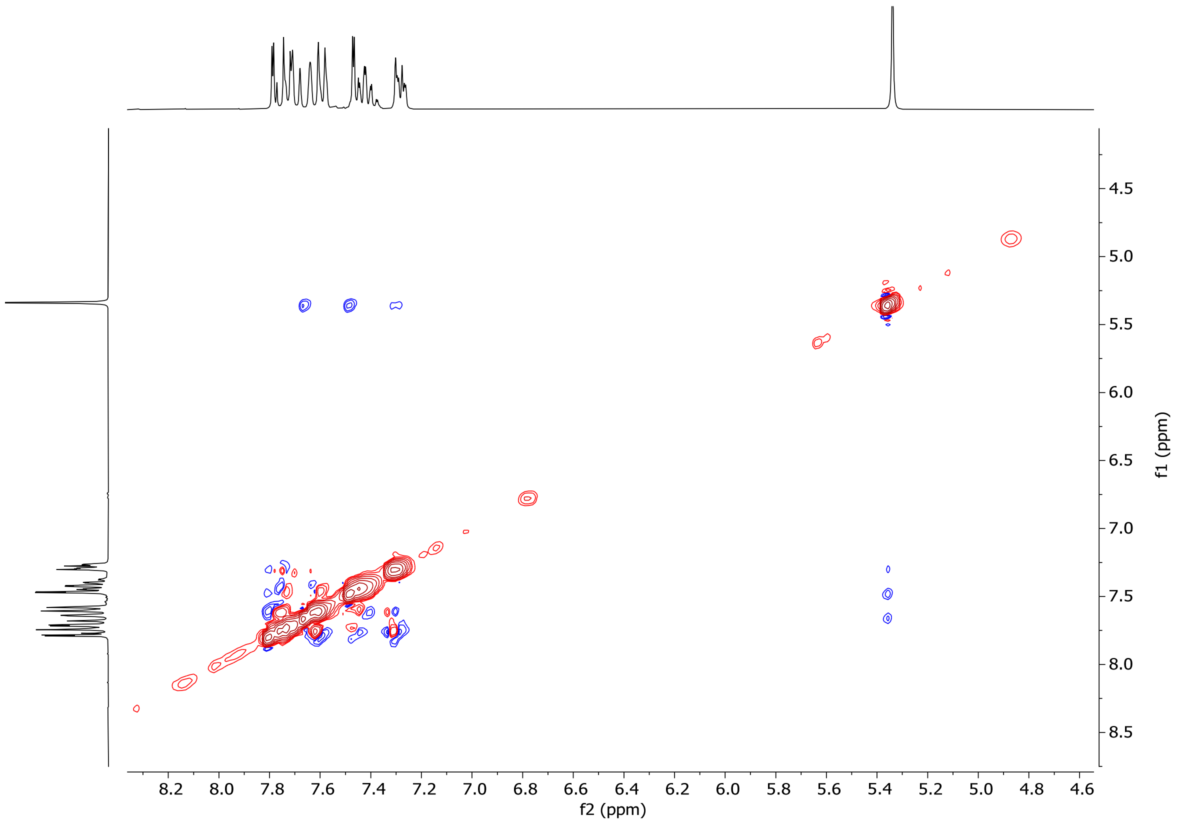

Figure S47. NOESY ^1H-^1H NMR spectrum of **Ag6**.

pAg1

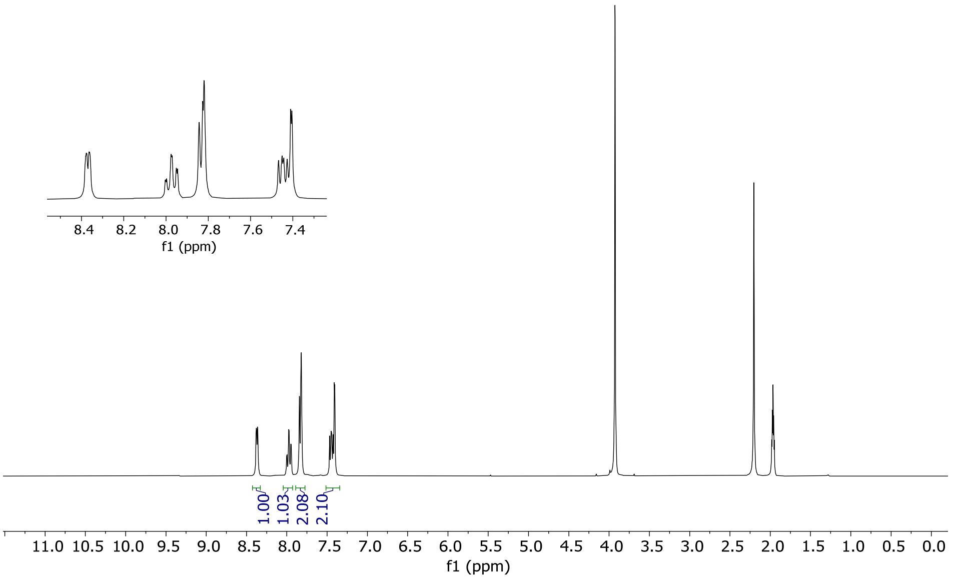

Figure S48. ^{1}H NMR spectrum of **pAg1**.

pAg2

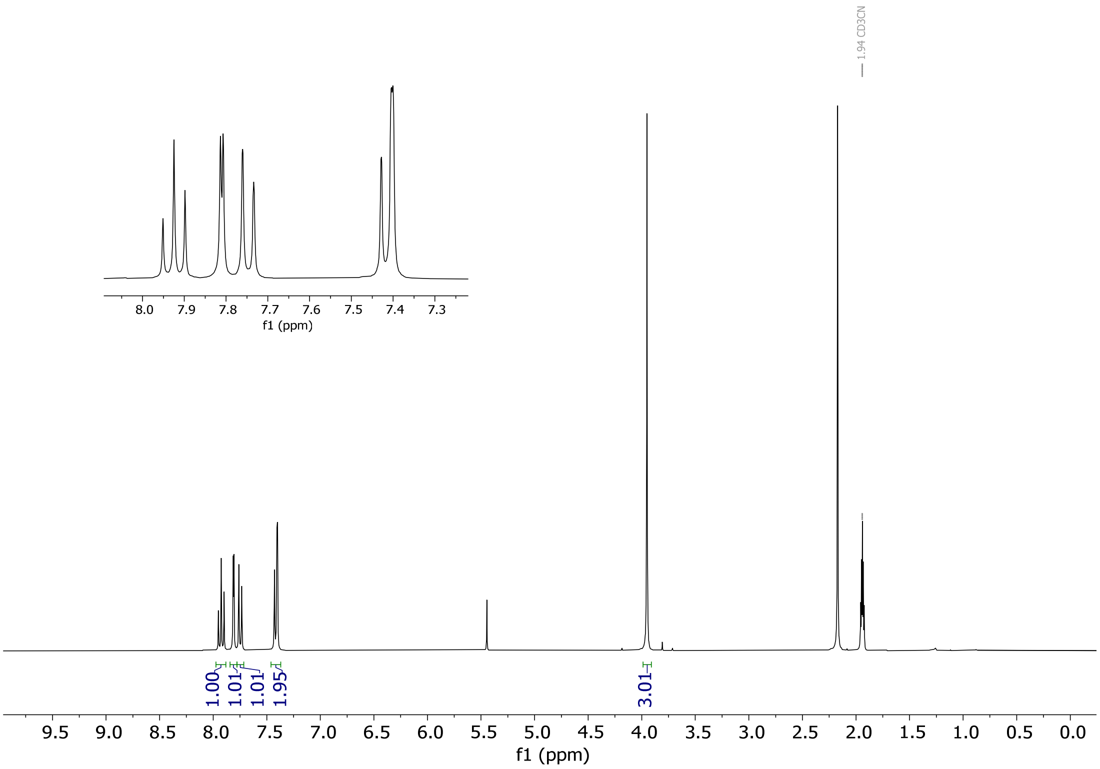

Figure S49. ^{1}H NMR spectrum of **pAg2**.

pAg3

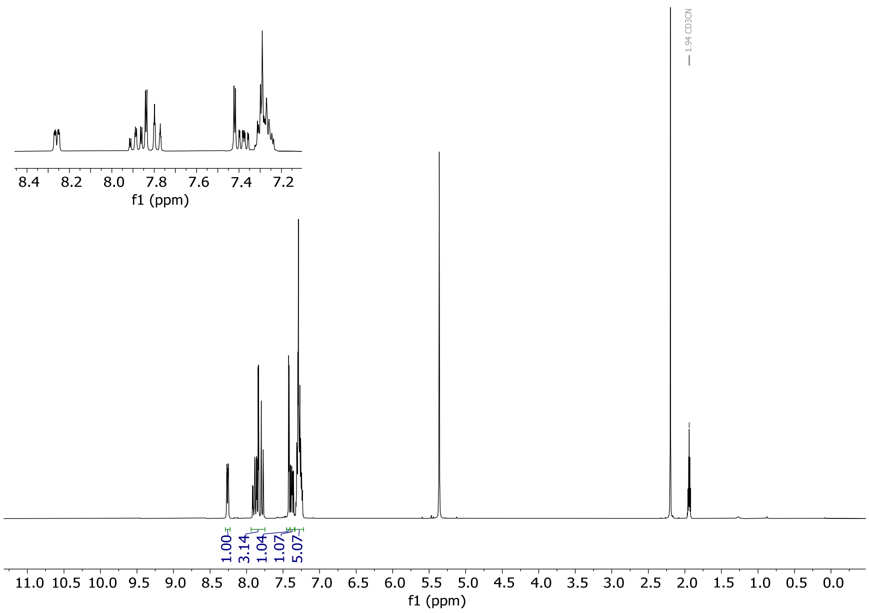

Figure S50. ^{1}H NMR spectrum of **pAg3**.

pAg4

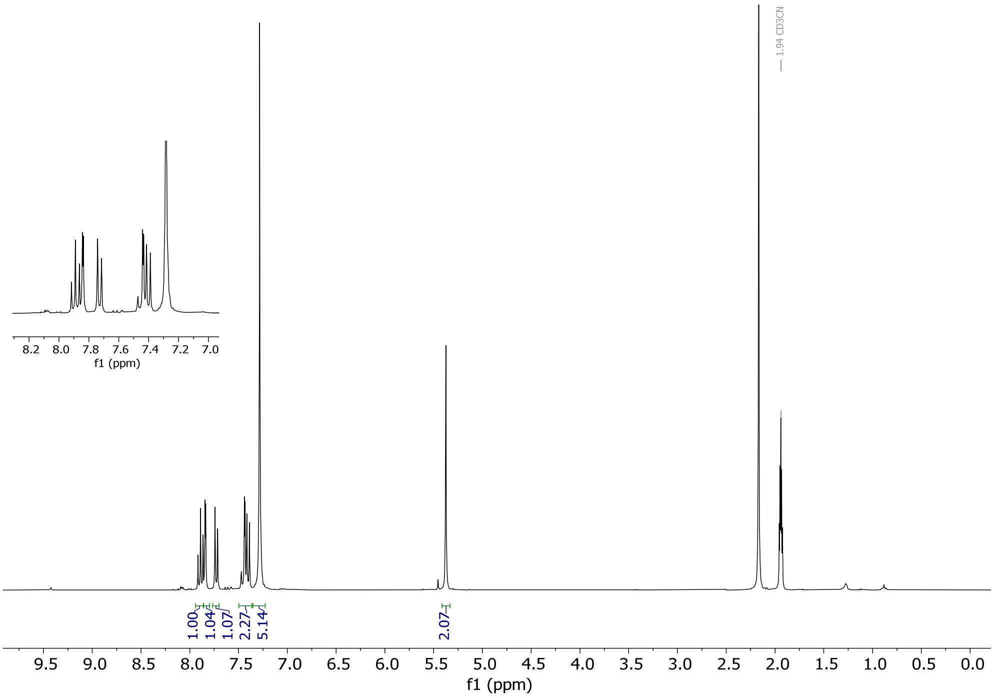

Figure S51. ^{1}H NMR spectrum of **pAg4**.

pAg5

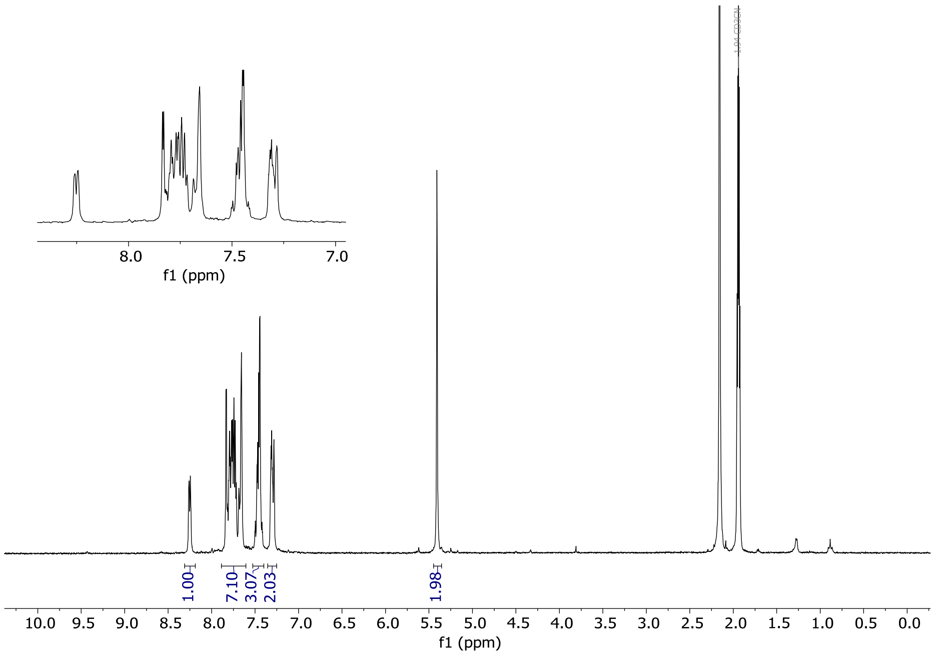

Figure S52. ^{1}H NMR spectrum of **pAg5**.

pAg6

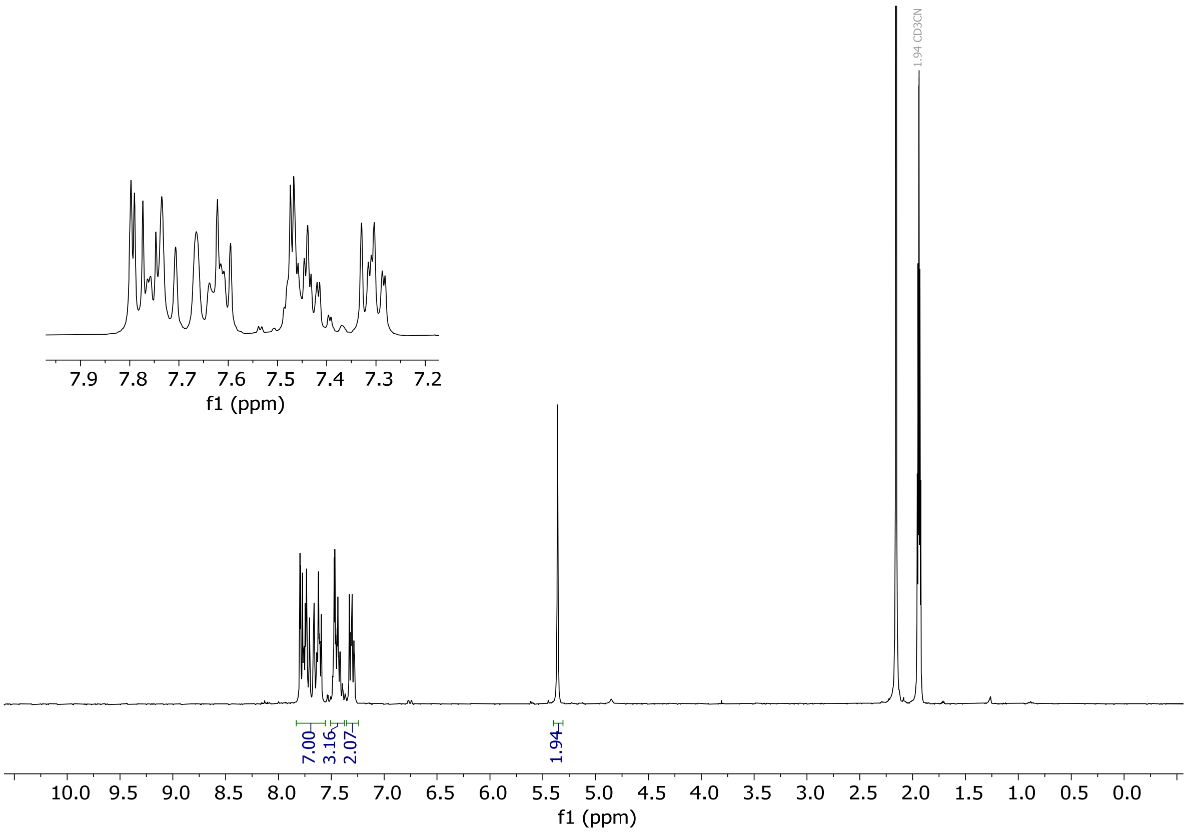

Figure S53. ^{1}H NMR spectrum of **pAg6**

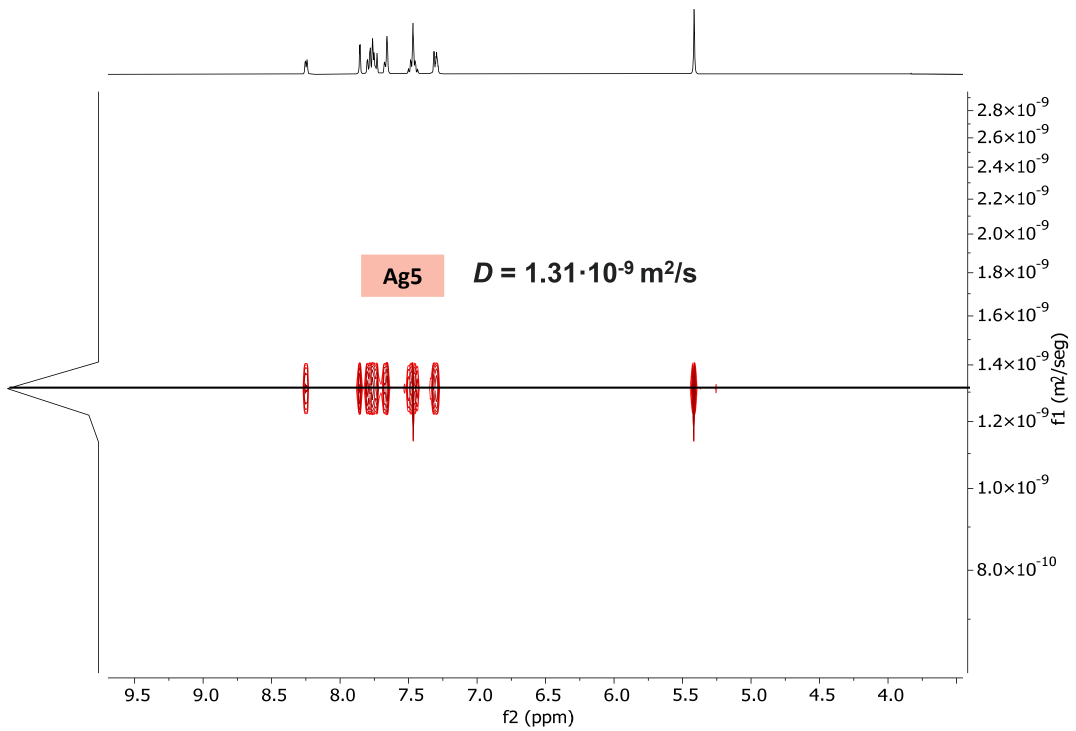

Figure S54. DOSY (^{1}H-^{1}H) of **Ag5** in CD_3CN, at constant concentration of 5 mM. The displayed diffusion coefficient is the result of calculating the average of the individual diffusion coefficients associated with each proton signal.

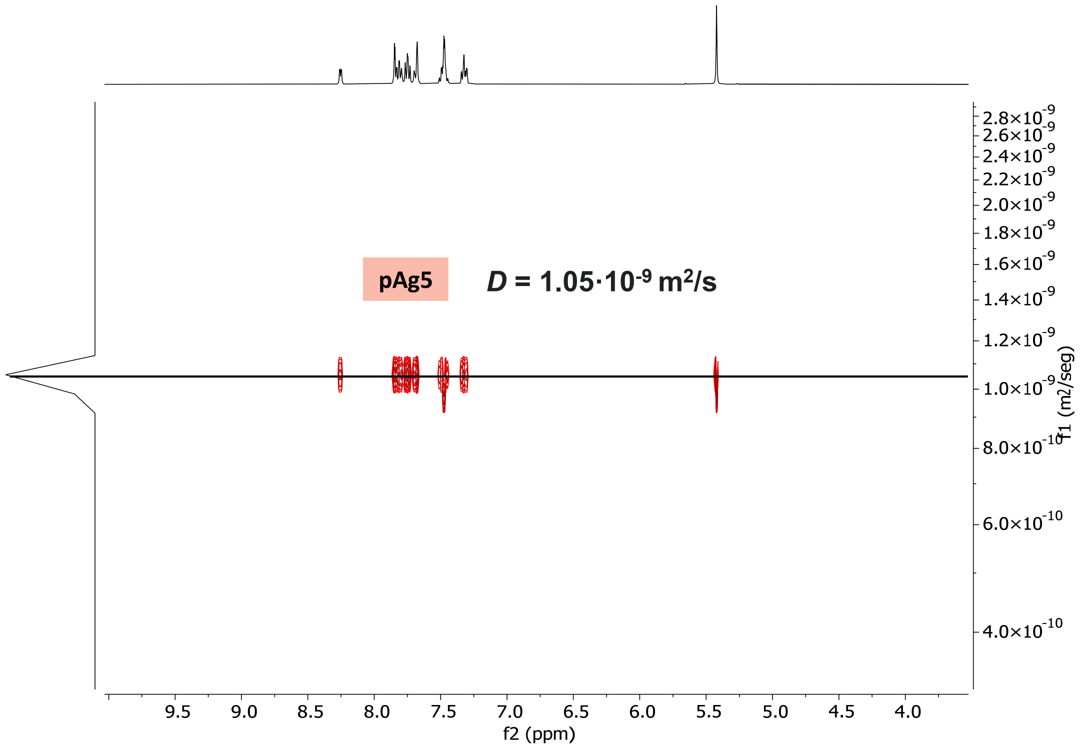

Figure S55. DOSY (^{1}H-^{1}H) of **pAg5** in CD_3CN, at constant concentration of 5 mM. The displayed diffusion coefficient is the result of calculating the average of the individual diffusion coefficients associated with each proton signal.

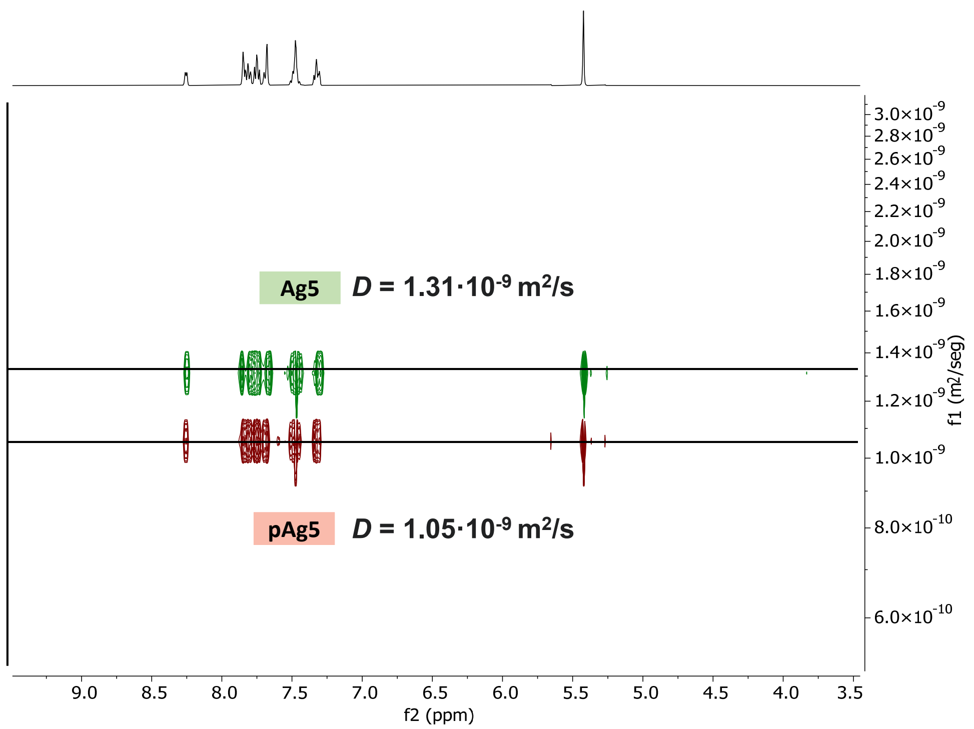

Figure S56. Superposition of DOSY (^{1}H-^{1}H) spectra of **Ag5** and **pAg5**

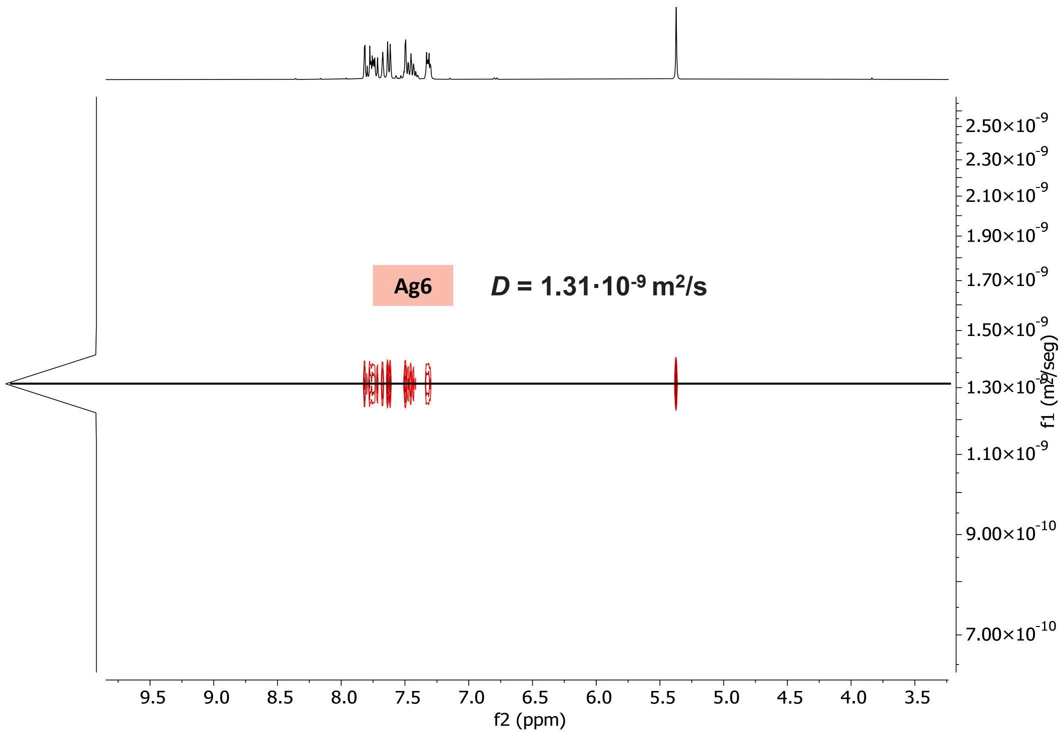

Figure S57. DOSY (^{1}H-^{1}H) of **Ag6** in CD_3CN, at constant concentration of 5 mM. The displayed diffusion coefficient is the result of calculating the average of the individual diffusion coefficients associated with each proton signal.

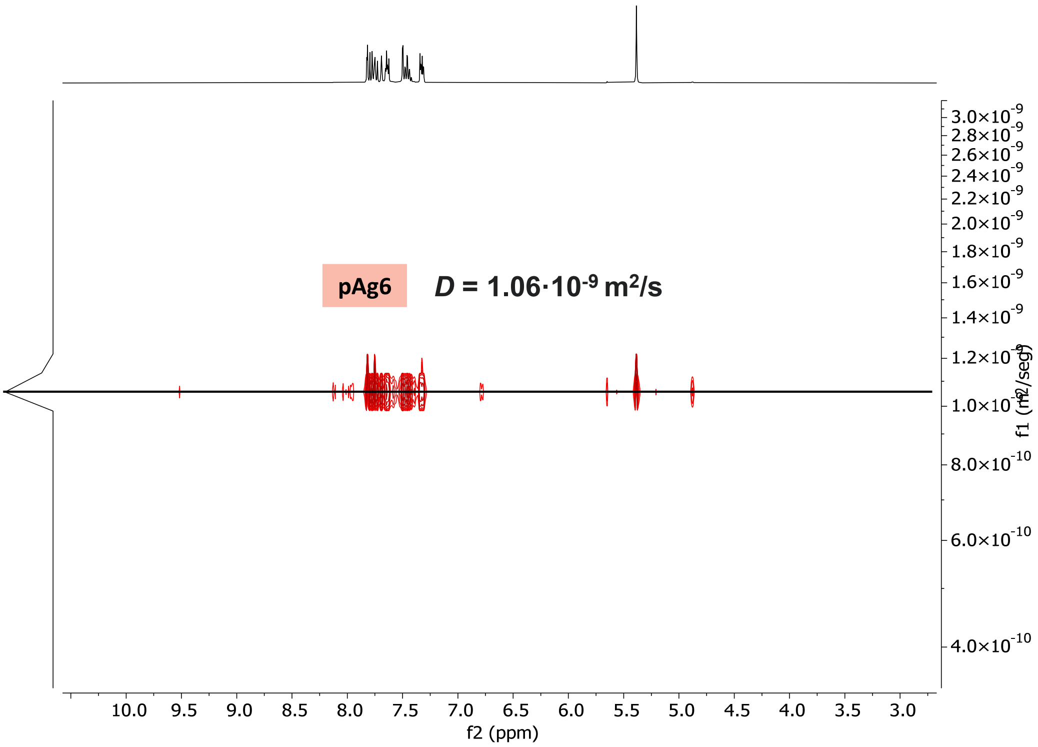

Figure S58. DOSY (^1H-^1H) of **pAg6** in CD_3CN, at constant concentration of 5 mM. The displayed diffusion coefficient is the result of calculating the average of the individual diffusion coefficients associated with each proton signal.

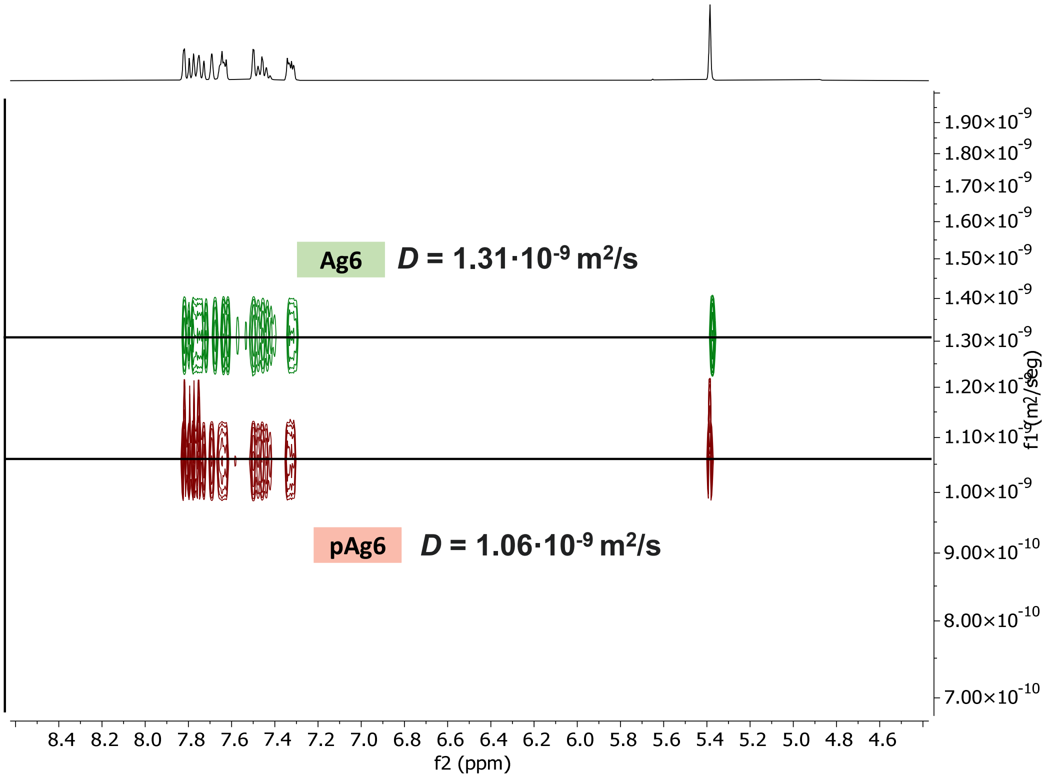

Figure S59. Superposition of DOSY (^1H-^1H) spectra of **Ag6** and **pAg6**

EMISSION AND EXCITATION SPECTRA

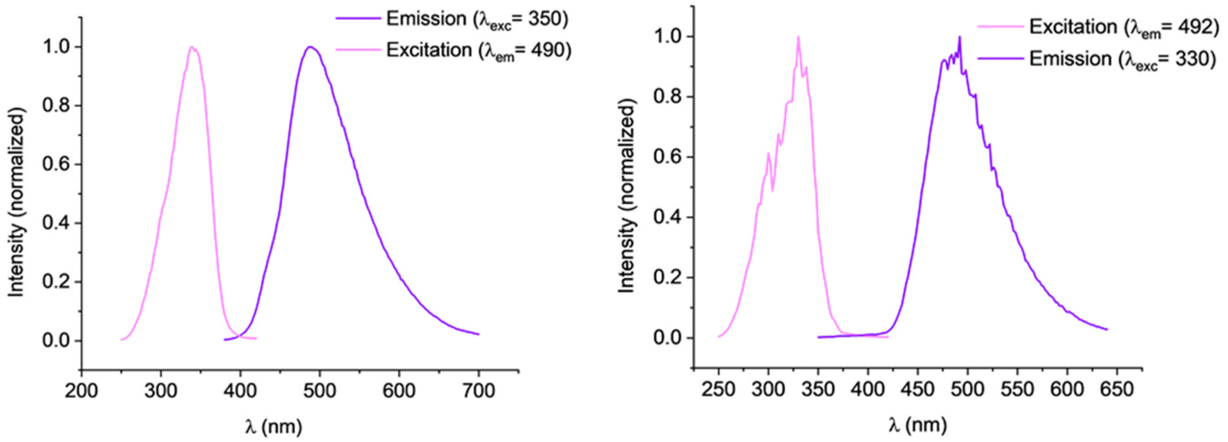

Figure S60. Excitation and emission spectra at rt (left) and 77K (right) of **Ag4**

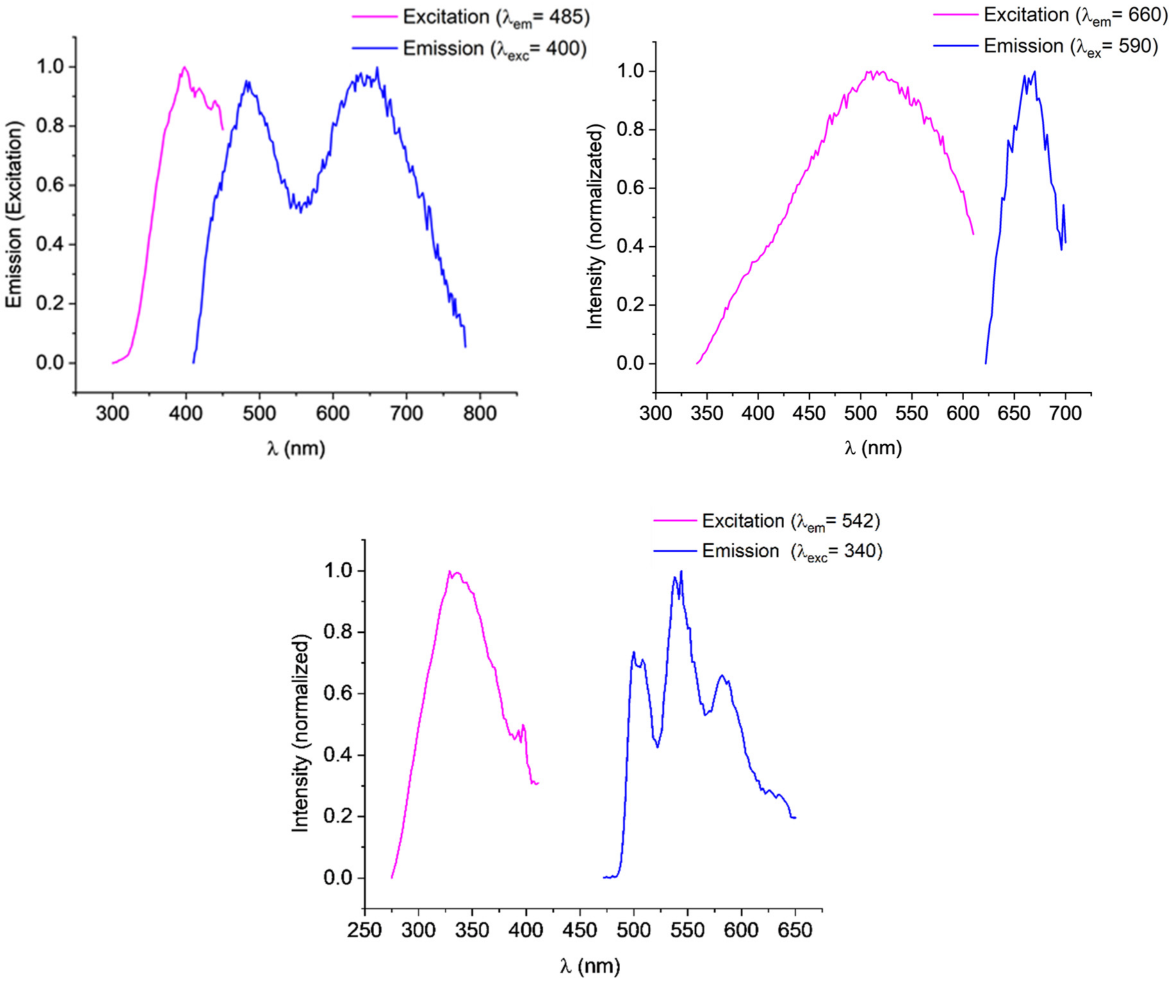

Figure S61. Excitation and emission spectra at rt (up) and 77K (bottom) of **Ag5**

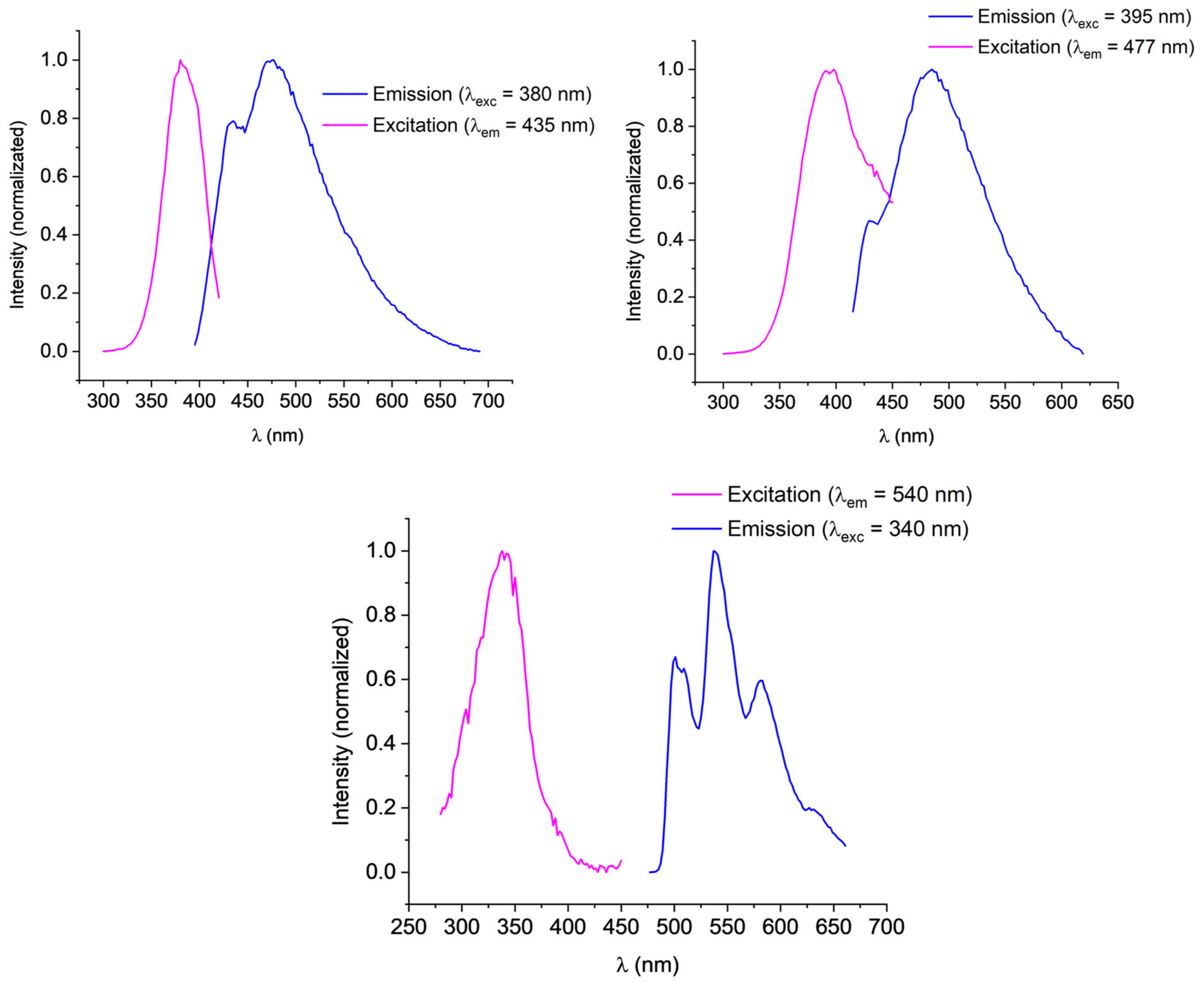

Figure S62. Excitation and emission spectra at rt (up) and 77K (bottom) of **Ag6**

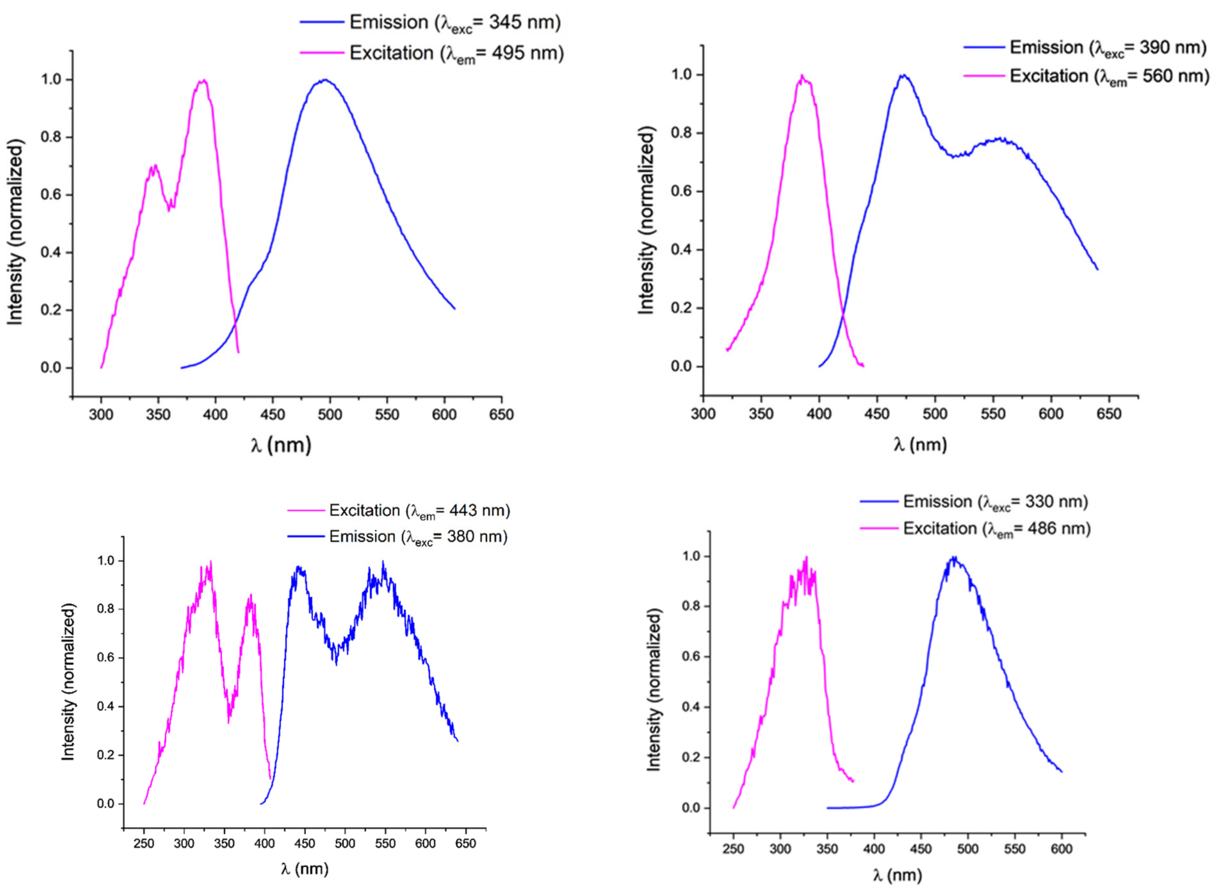

Figure S63. Excitation and emission spectra at rt (up) and 77K (bottom) of **pAg3**

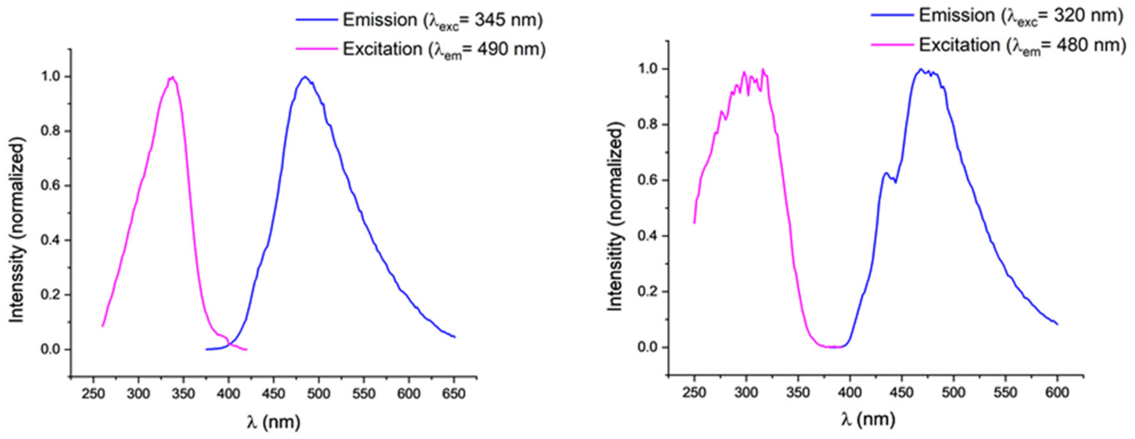

Figure S64. Excitation and emission spectra at rt (left) and 77K (right) of **pAg4**

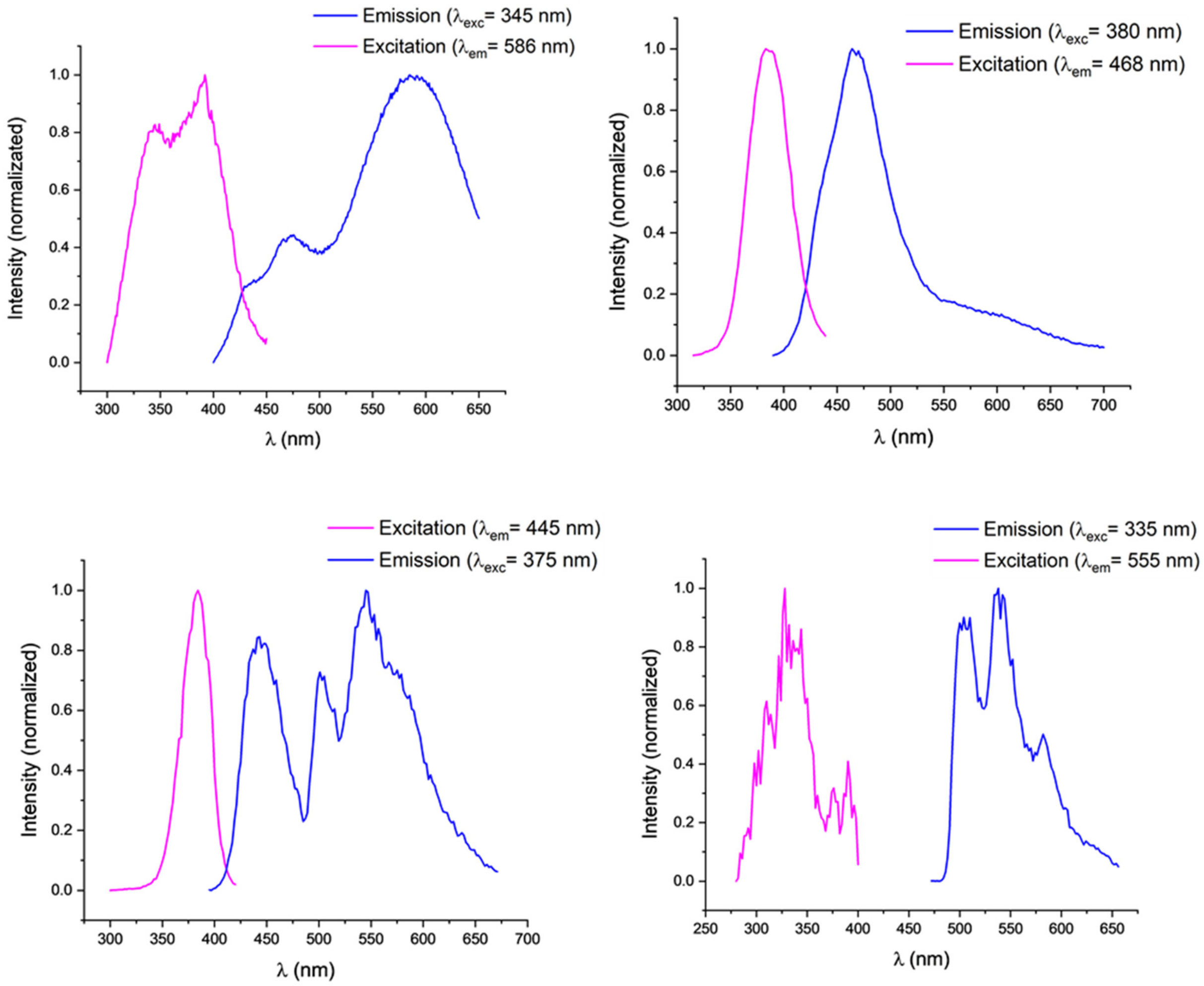

Figure S65. Excitation and emission spectra at rt (up) and 77K (bottom) of **pAg5**

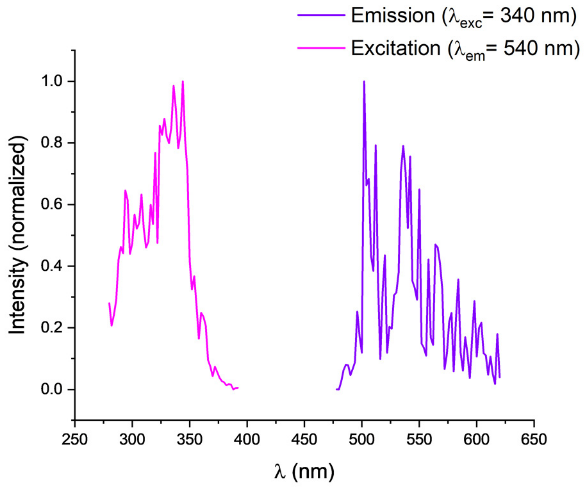

Figure S66. Excitation and emission spectra at 77K of **pAg6**

LIFETIME FITTING CURVES

Ag4 (rt)

Container Properties

Measurement Context: Decay
Summary:

Sample:	Sample_8
Solvent:	<unspecified>
Excitation:	U pol 350±5nm with Xe-Lamp
Detection:	U pol 485±5nm 10000 peak counts<br>grating 1200/500+<br>detector UV-red [PMT]

Fitted Parameters

Parameter	Value	Δ	δ
A_1[kCnts/Chnl]	7.175	±0.026	0.4%
τ_1[ns]	18 136	±48	0.3%
I_1[kCnts]	406.64	±0.52	0.1%
$Bkgr_{Dec}$[kCnts]	0.0044	±0.0001	0.3%
τ_{AvInt}[ns]	18 136	±48	0.3%

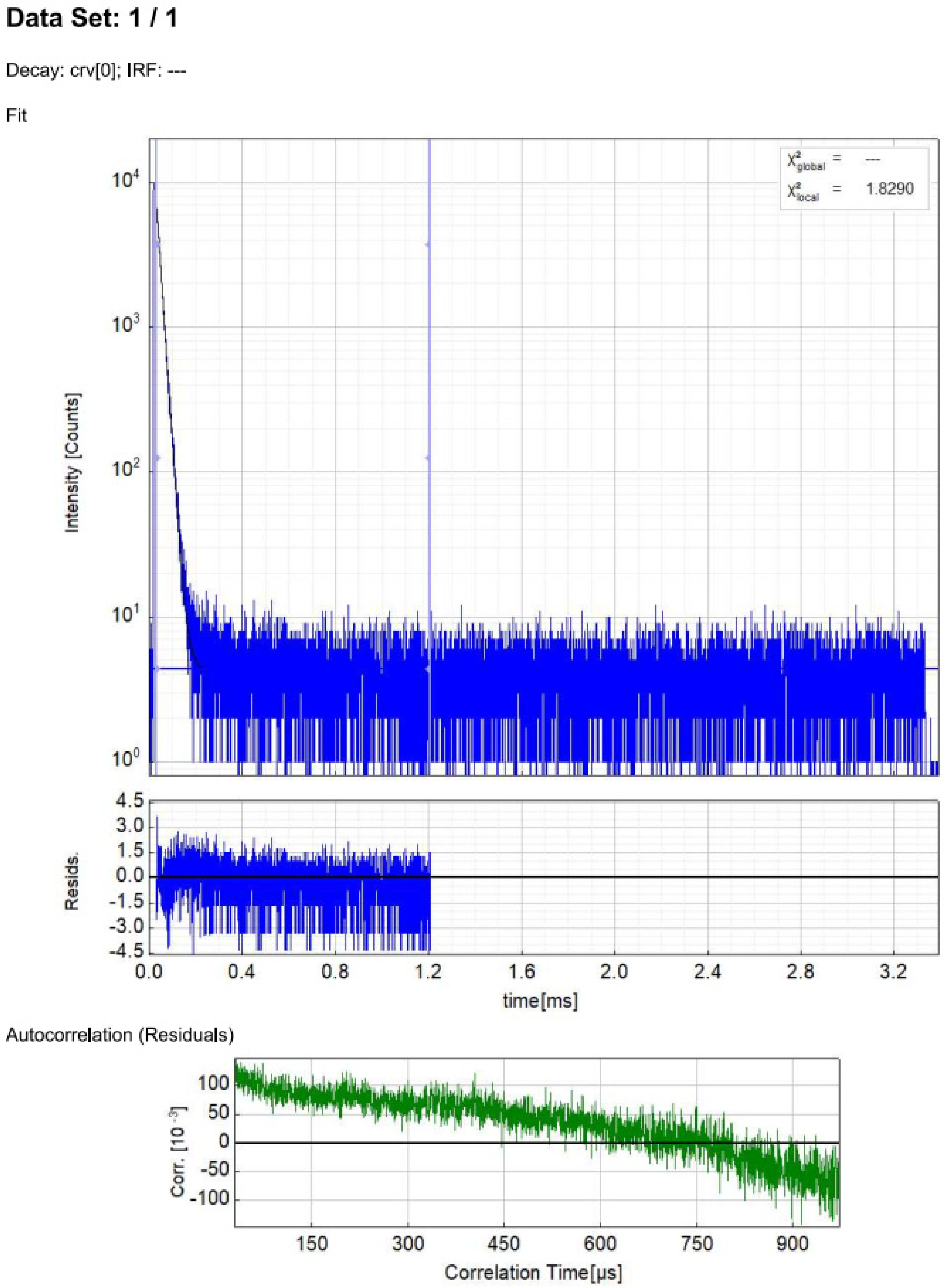

Figure S67. Fitting curve for **Ag4** at room temperature

Ag4 (77K)

Container Properties

Measurement Context: Decay
Summary:
Sample: IBP-91-S-TV-77K-PUL-ex330-em495
Solvent: <unspecified>
Excitation: U pol 330±5nm with Xe-Lamp
Detection: U pol 495±5nm 100000 peak counts
grating 1200/500+
detector UV-red [PMT]

Fitted Parameters

Parameter	Value	Δ	δ
A_1[kCnts/Chnl]	0.26	±0.12	42%
τ_1[ns]	184 000	±31 000	17%
I_1[kCnts]	9.4	±2.8	30%
A_2[kCnts/Chnl]	75.99	±0.13	0.2%
τ_2[ns]	59 650	±150	0.2%
I_2[kCnts]	885.1	±2.4	0.3%
$Bkgr_{Dec}$[kCnts]	0.0212	±0.0003	1.1%
τ_{AvInt}[ns]	60 937	±70	0.1%

Data Set: 1 / 1

Decay: crv[0]; IRF: ---

Fit

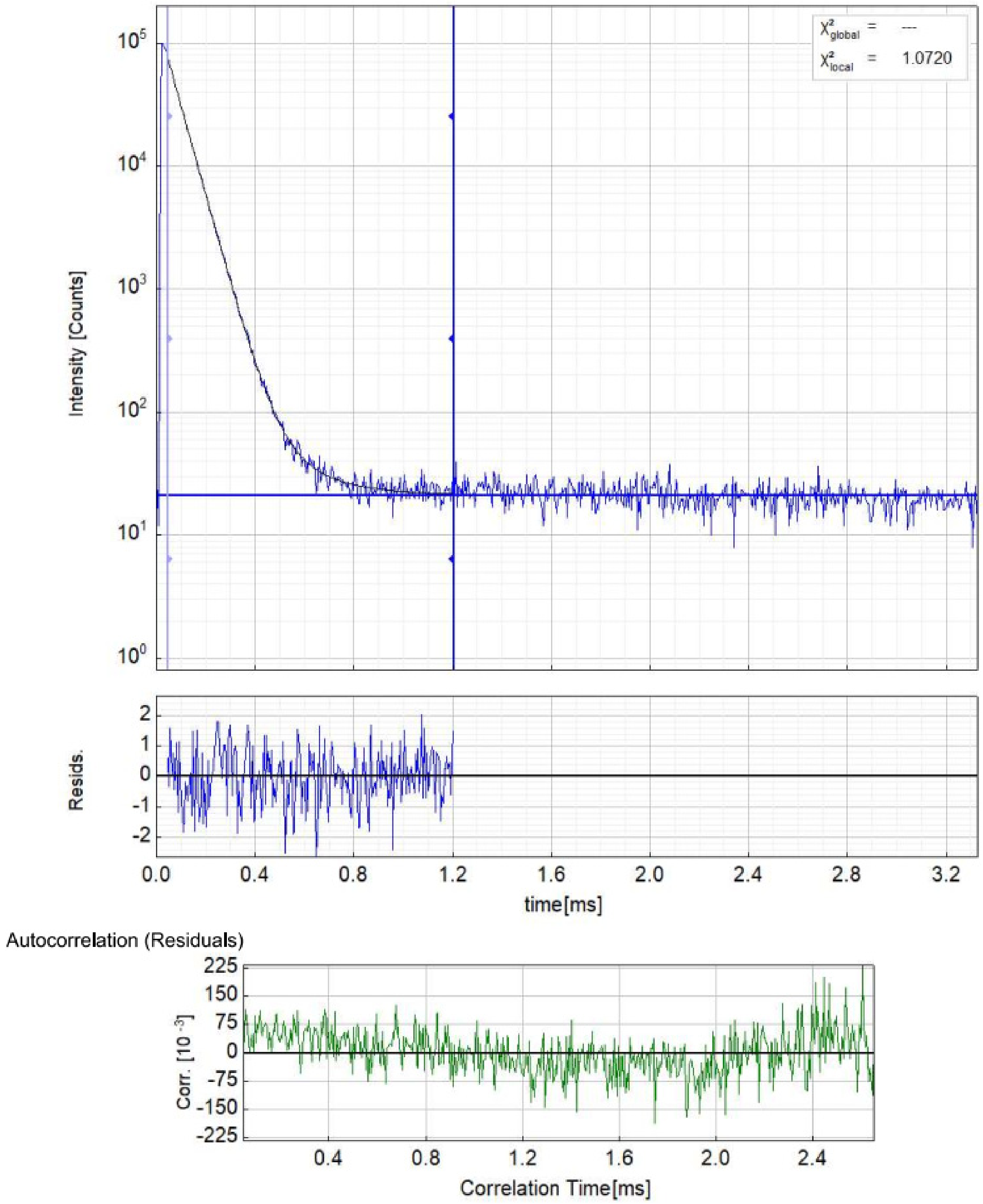

Figure S68. Fitting curve for **Ag4** at 77 K

Ag5 (rt (λ exc = 400 nm)

Container Properties

Measurement Context: Decay

Summary:

Sample:	Sample_14
Solvent:	<unspecified>
Excitation:	U pol 405±15nm with PLS-400
Detection:	U pol 480±8nm 100000 peak counts<br>grating 1200/500+<br>detector UV-red [PMT]

Fitted Parameters

Parameter	Value	Δ	δ
A_1[kCnts/Chnl]	0.0384	±0.0075	19%
τ_1[ns]	172 000	±19 000	11%
I_1[kCnts]	1.284	±0.082	6.4%
A_2[kCnts/Chnl]	0.1020	±0.0064	6.2%
τ_2[ns]	30 200	±6 200	20%
I_2[kCnts]	0.61	±0.11	17%
$Bkgr_{Dec}$[kCnts]	0.0012	±0.0002	8.5%
τ_{AvInt}[ns]	126 500	±6 700	5.3%

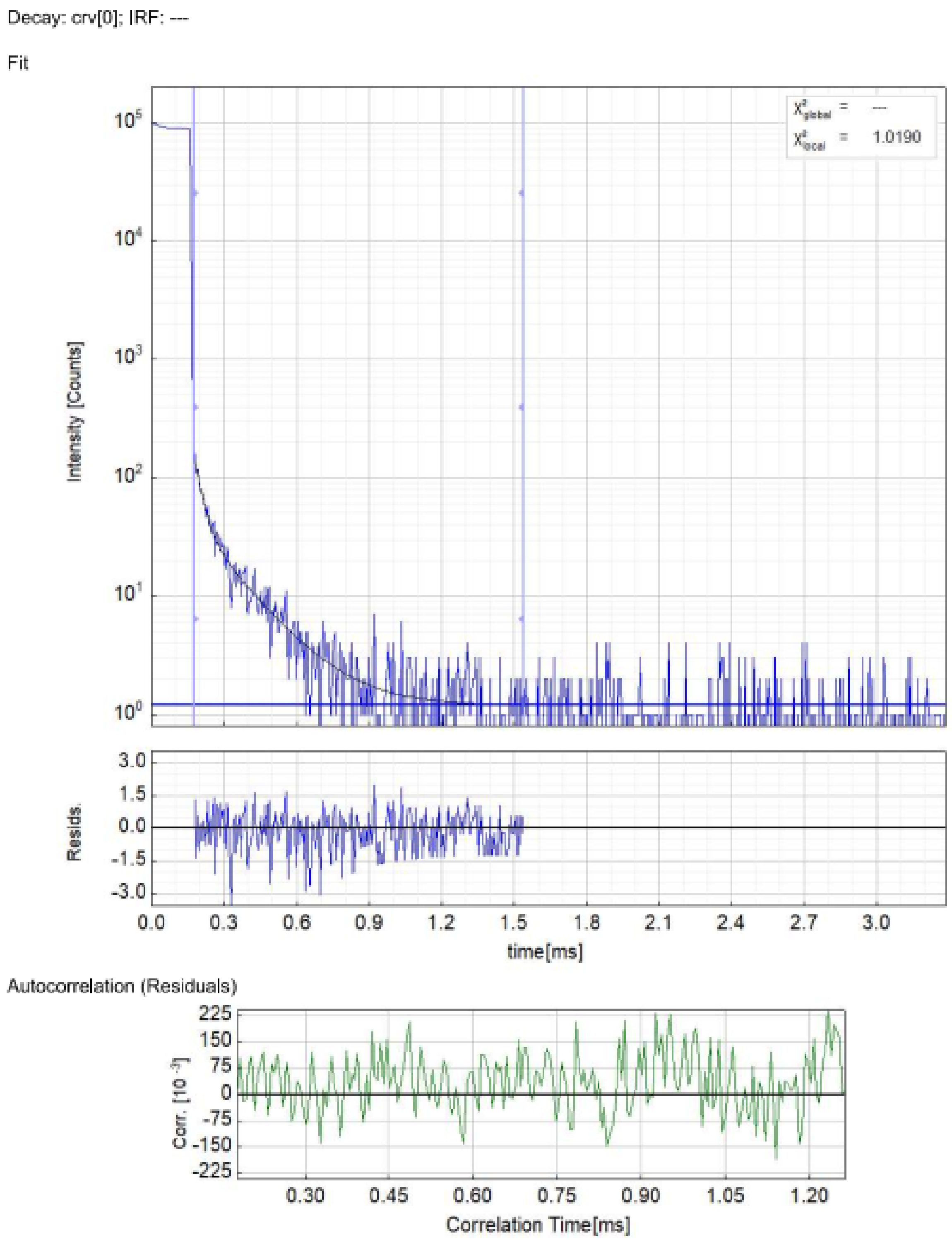

Figure S69. Fitting curve for **Ag5** at room temperature (λ exc = 400 nm).

Ag5 (rt (λ exc = 530 nm selected in order to obtain a clearer spectrum)

Container Properties

Measurement Context: Decay

Summary:

Sample:	Sample_4
Solvent:	<unspecified>
Excitation:	U pol 530±14nm with Xe-Lamp
Detection:	U pol 612±14nm 100000 peak counts<br>grating 1200/500+<br>detector UV-red [PMT]

Fitted Parameters

Parameter	Value	Δ	δ
A_1[kCnts/Chnl]	0.665	±0.063	9.4%
τ_1[ns]	208 900	±5 900	2.8%
I_1[kCnts]	27.2	±1.8	6.3%
A_2[kCnts/Chnl]	1.729	±0.044	2.5%
τ_2[ns]	74 900	±2 800	3.6%
I_2[kCnts]	25.3	±1.7	6.7%
$Bkgr_{Dec}$[kCnts]	0.0034	±0.0002	5.2%
τ_{AvInt}[ns]	144 240	±580	0.4%

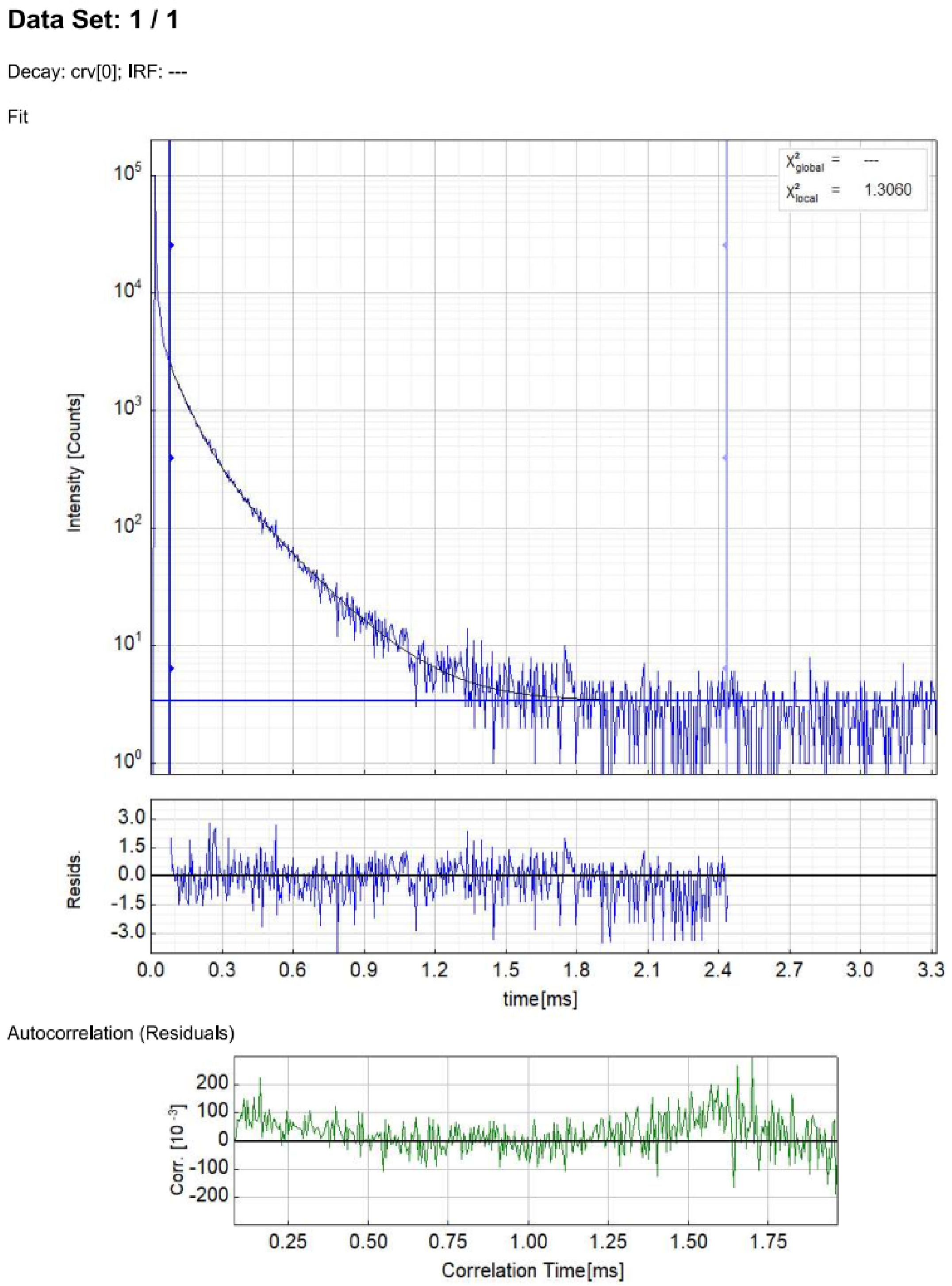

Figure S70. Fitting curve for **Ag5** at room temperature (λ exc = 530 nm).

Ag5 (77K)

Container Properties

Measurement Context: Decay
Summary:
Sample: IBP-109-TV-77K-FLASH-XENON
Solvent: <unspecified>
Excitation: U pol 340±5nm with Xe-Lamp
Detection: U pol 540±5nm 10000 peak counts
grating 1200/500+
detector UV-red [PMT]

Fitted Parameters

Parameter	Value	Δ	δ
A_1[kCnts/Chnl]	0.782	±0.053	6.7%
τ_1[ns]	68 900 000	±3 000 000	4.3%
I_1[kCnts]	41.08	±0.85	2.1%
A_2[kCnts/Chnl]	2.265	±0.060	2.6%
τ_2[ns]	17 950 000	±730 000	4.0%
I_2[kCnts]	31.1	±1.2	3.6%
$Bkgr_{Dec}$[kCnts]	0.0472	±0.0016	3.3%
τ_{AvInt}[ns]	47 000 000	±1 400 000	2.8%

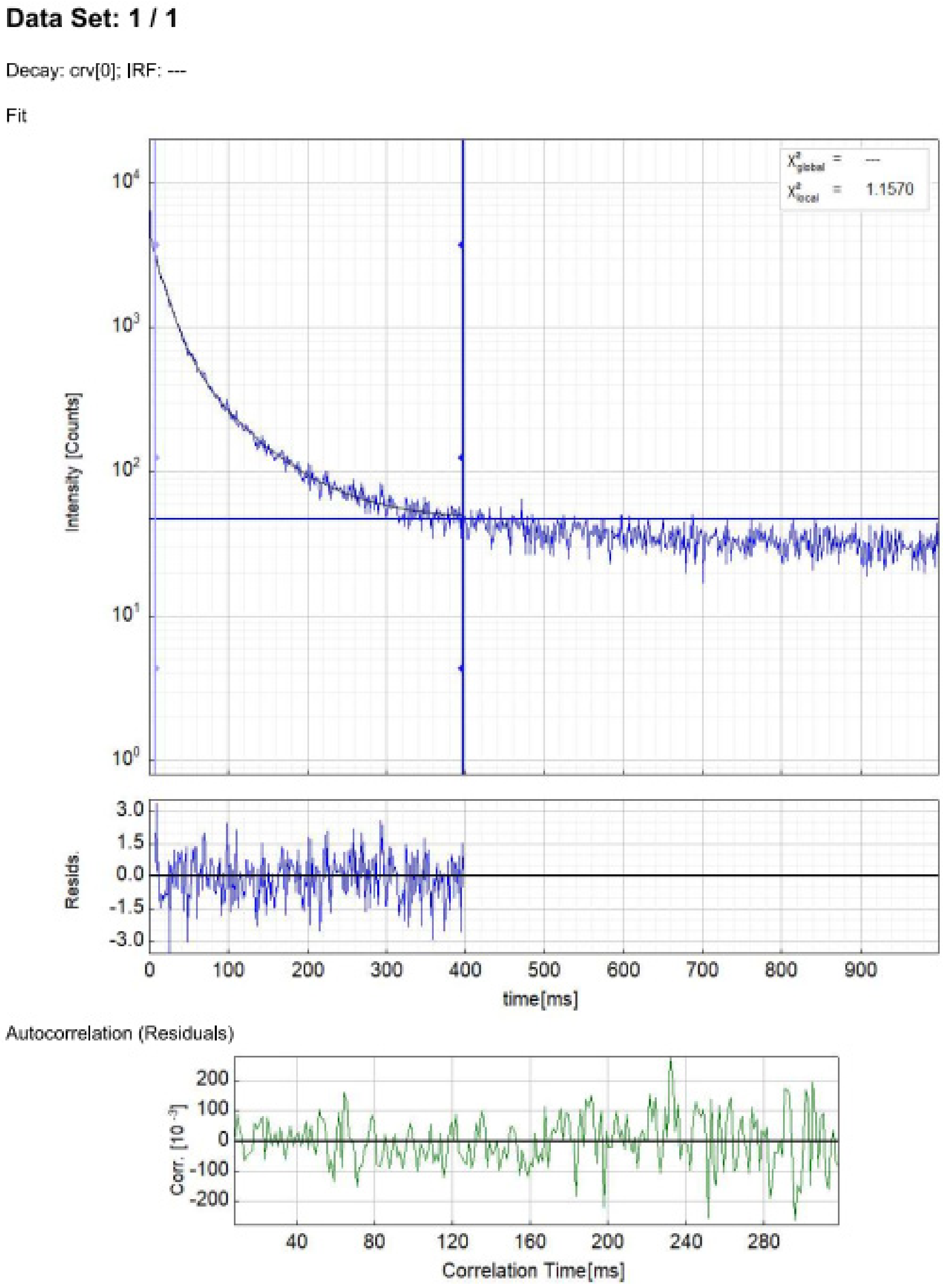

Figure S71. Fitting curve for **Ag5** 77 K.

Ag6 rt (λ exc = 400 nm)

Container Properties

Measurement Context: Decay
Summary:
Sample: Sample_8
Solvent: <unspecified>
Excitation: U pol 405±15nm with PLS-400
Detection: U pol 477±14nm 100000 peak counts
grating 1200/500+
detector UV-red [PMT]

Fitted Parameters

Parameter	Value	Δ	δ
A_1[kCnts/Chnl]	0.0412	±0.0066	16%
τ_1[ns]	336 000	±37 000	11%
I_1[kCnts]	2.70	±0.19	7.0%
A_2[kCnts/Chnl]	0.1637	±0.0064	3.9%
τ_2[ns]	62 700	±7 900	12%
I_2[kCnts]	2.01	±0.23	11%
$Bkgr_{Dec}$[kCnts]	0.0029	±0.0003	9.2%
τ_{AvInt}[ns]	220 000	±16 000	7.1%

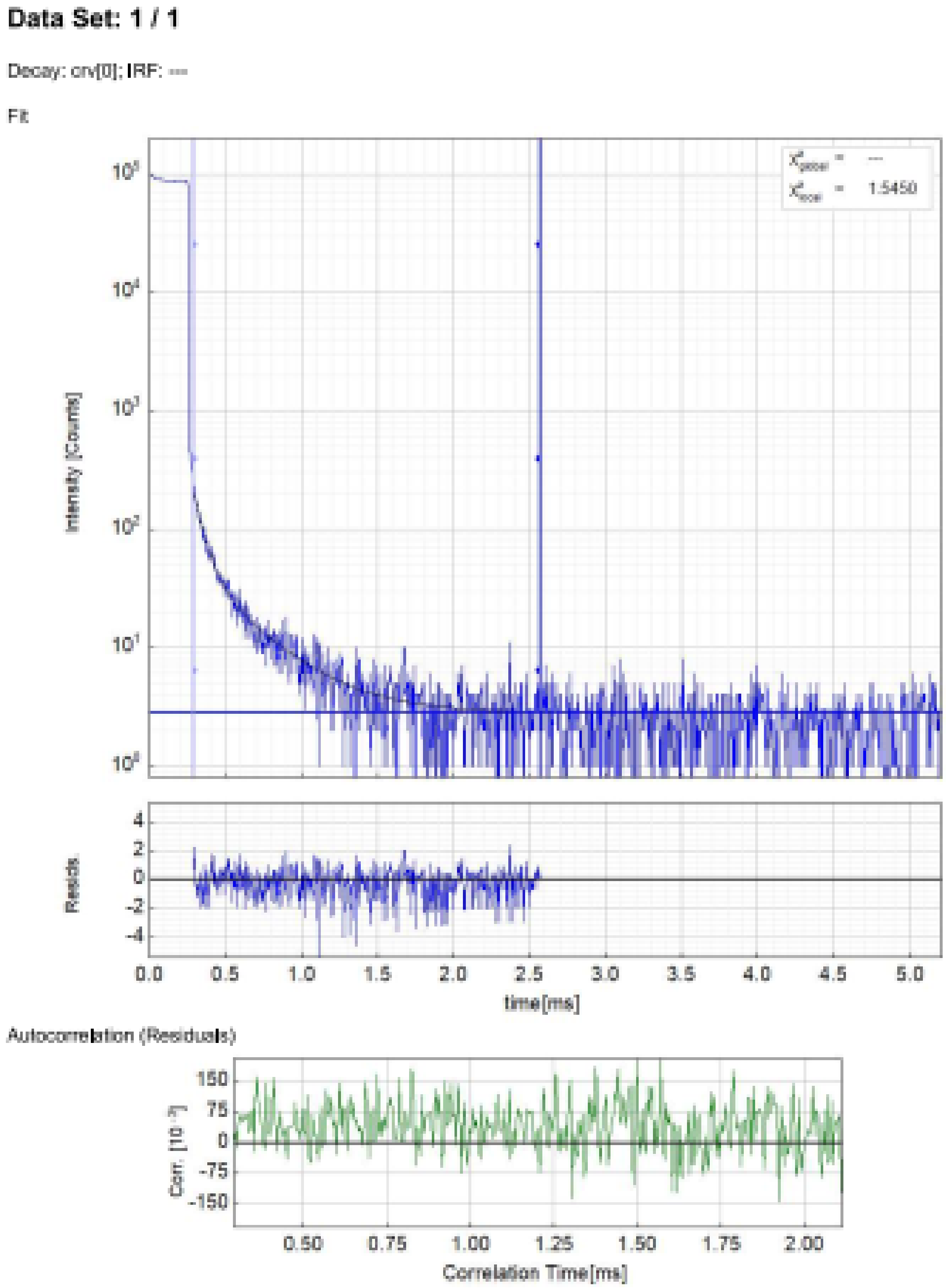

Figure S72. Fitting curve for **Ag6** at room temperature (λ exc = 400 nm).

Ag6 rt (λ exc = 380nm

Container Properties

Measurement Context: Decay
Summary:

Sample:	TV-FLASH-XENON
Solvent:	<unspecified>
Excitation:	U pol 380±8nm with Xe-Lamp
Detection:	U pol 480±8nm 900000 peak counts<br>grating 1200/500+<br>detector UV-red [PMT]

Fitted Parameters

Parameter	Value	Δ	δ
A_1[kCnts/Chnl]	0.0258	±0.0009	3.4%
τ_1[ns]	211 000	±17 000	7.8%
I_1[kCnts]	0.532	±0.052	9.7%
$Bkgr_{Dec}$[kCnts]	0.0036	±0.0002	4.9%
τ_{AvInt}[ns]	211 000	±17 000	7.8%

Data Set: 1 / 1

Decay: crv[0]; IRF: ---

Fit

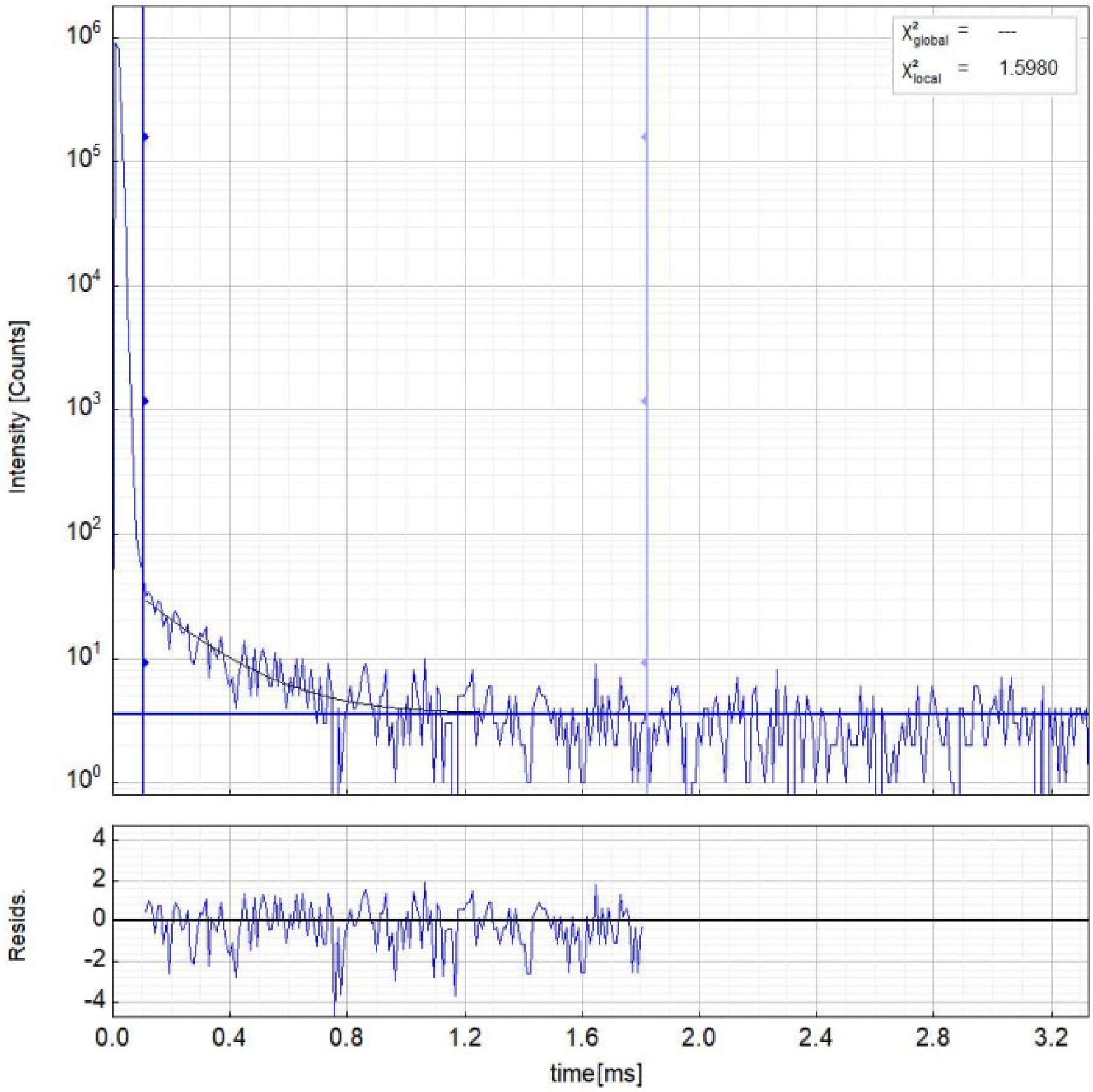

Autocorrelation (Residuals)

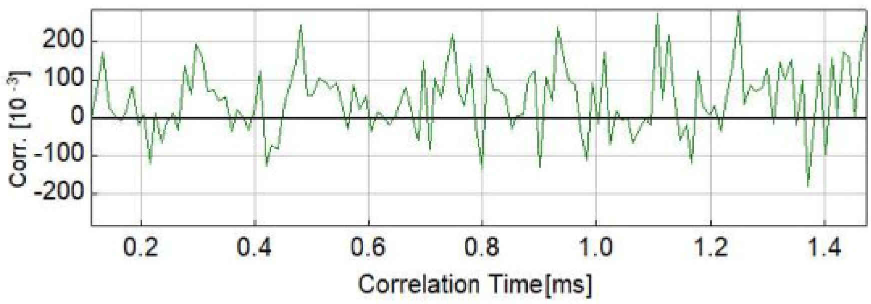

Figure S73. Fitting curve for **Ag6** at room temperature (λ exc = 380 nm).

Ag6 (77K)

Container Properties

Measurement Context: Decay
Summary:
Sample: TV-77K-FLASH-XENON
Solvent: <unspecified>
Excitation: U pol 340±5nm with Xe-Lamp
Detection: U pol 540±5nm 10000 peak counts
grating 1200/500+
detector UV-red [PMT]

Fitted Parameters

Parameter	Value	Δ	δ
A_1[kCnts/Chnl]	0.333	±0.036	11%
τ_1[ns]	48 600 000	±2 800 000	5.6%
I_1[kCnts]	24.7	±1.2	4.8%
A_2[kCnts/Chnl]	0.862	±0.033	3.7%
τ_2[ns]	15 750 000	±630 000	4.0%
I_2[kCnts]	20.7	±1.6	7.5%
$Bkgr_{Dec}$[kCnts]	0.0112	±0.0006	4.8%
τ_{AvInt}[ns]	33 550 000	±810 000	2.4%

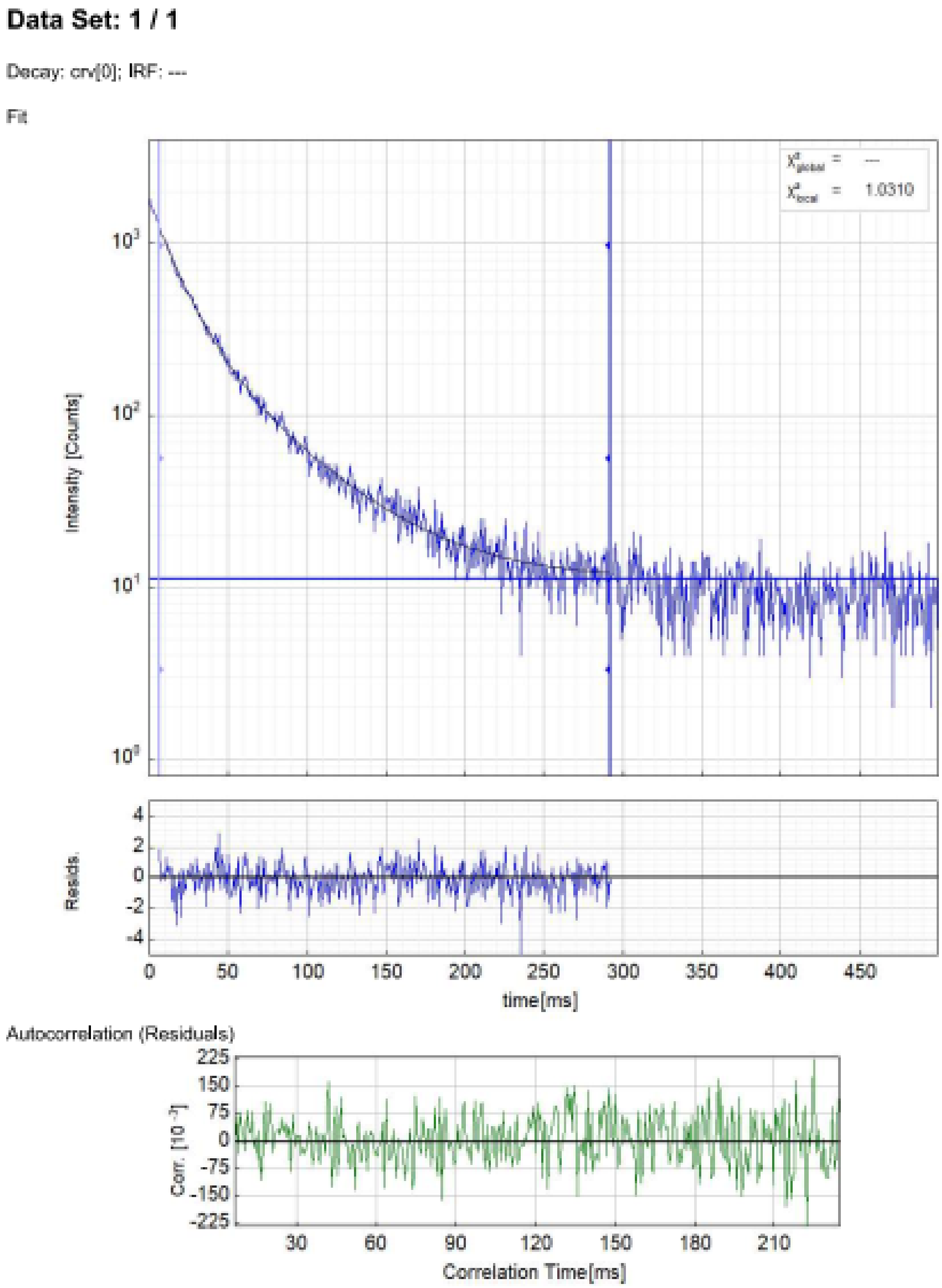

Figure S74. Fitting curve for **Ag6** at 77 K.

pAg3 rt (λ exc = 345nm)

Container Properties

Measurement Context: Decay

Summary:

Sample:	IBP-50-TV-FlashXe-em490
Solvent:	<unspecified>
Excitation:	U pol 345±5nm with Xe-Lamp
Detection:	U pol 490±5nm 90000 peak counts<br>grating 1200/500+<br>detector UV-red [PMT]

Fitted Parameters

Parameter	Value	Δ	δ
A_1[kCnts/Chnl]	0.370	±0.040	11%
τ_1[ns]	176 000	±12 000	6.7%
I_1[kCnts]	12.65	±0.54	4.2%
A_2[kCnts/Chnl]	2.125	±0.032	1.5%
τ_2[ns]	49 700	±1 300	2.5%
I_2[kCnts]	20.63	±0.82	4.0%
$Bkgr_{Dec}$[kCnts]	0.0071	±0.0009	12%
τ_{AvInt}[ns]	97 500	±2 500	2.5%

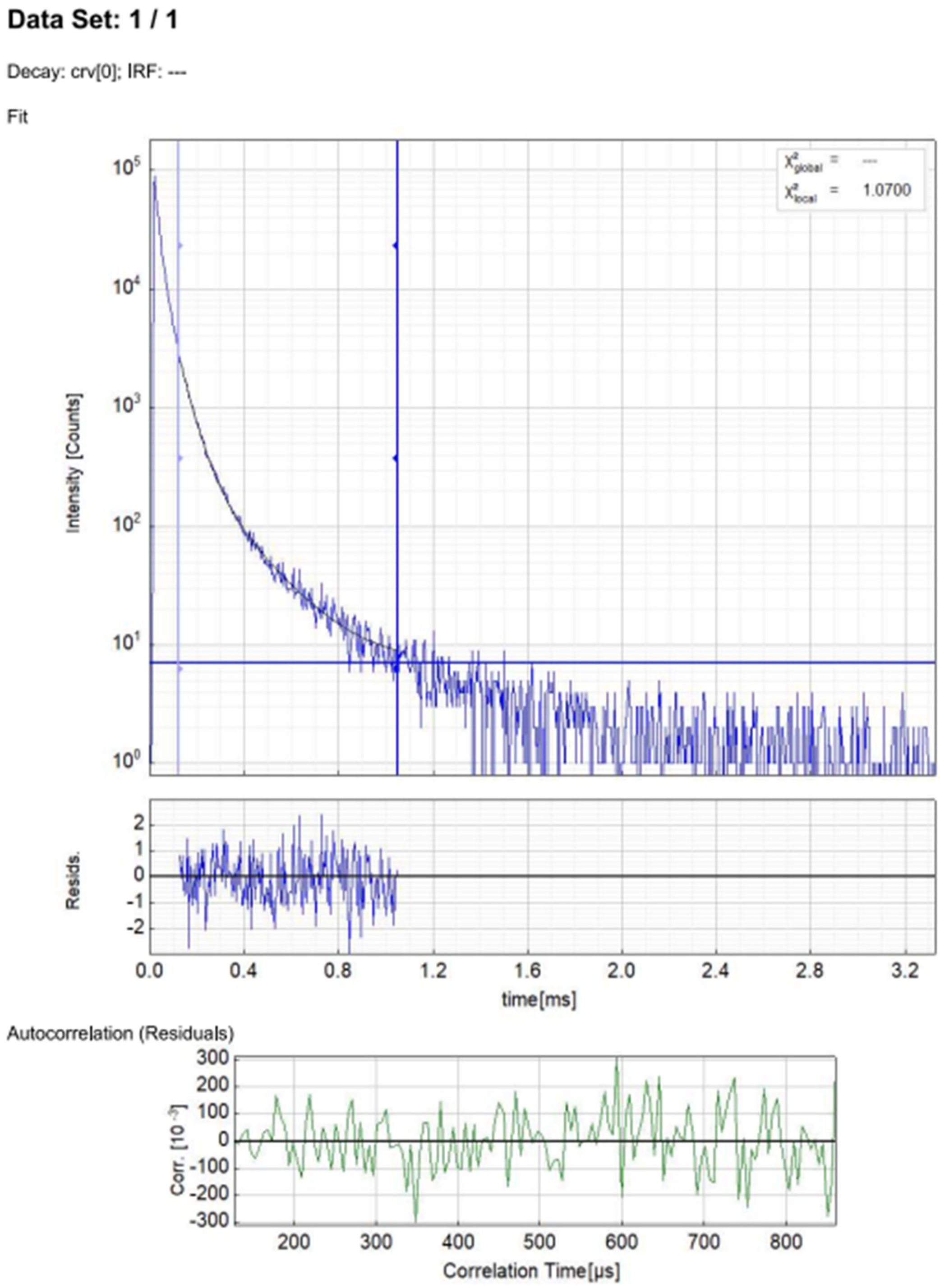

Figure S75. Fitting curve for **pAg3** at room temperature (λ exc = 345 nm)

pAg3 rt (λ exc = 390nm)

Container Properties

Measurement Context: Decay

Summary:

Sample:	IBP-50-TV-FlashXe-em555
Solvent:	<unspecified>
Excitation:	U pol 390±5nm with Xe-Lamp
Detection:	U pol 555±5nm 90000 peak counts<br>grating 1200/500+<br>detector UV-red [PMT]

Fitted Parameters

Parameter	Value	Δ	δ
A_1[kCnts/Chnl]	5.0	±1.9	36%
τ_1[ns]	121 200	±9 000	7.4%
I_1[kCnts]	118	±33	28%
A_2[kCnts/Chnl]	22.01	±0.69	3.1%
τ_2[ns]	62 400	±4 300	6.8%
I_2[kCnts]	269	±25	9.3%
A_3[kCnts/Chnl]	10.66	±0.48	4.5%
τ_3[ns]	14 200	±1 200	8.1%
I_3[kCnts]	29.6	±4.6	15%
$Bkgr_{Dec}$[kCnts]	0.0075	±0.0010	12%
τ_{AvInt}[ns]	75 590	±270	0.4%

Data Set: 1 / 1

Decay: crv[0]; IRF: ---

Fit

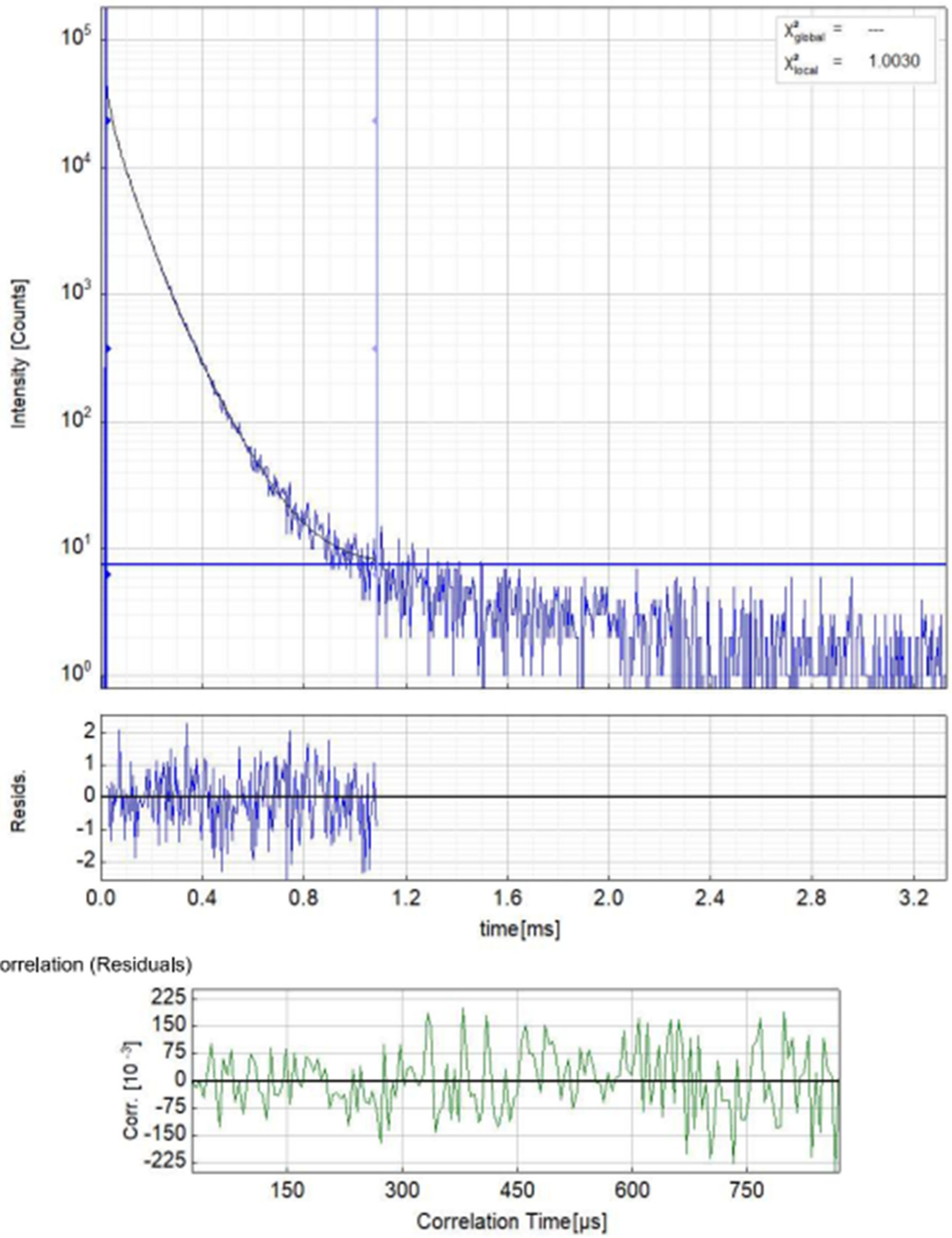

Figure S76. Fitting curve for **pAg3** at room temperature (λ exc = 390 nm)

pAg3 (77K)

Container Properties

Measurement Context: Decay
Summary:
Sample: IBP-50-TV-77K-exc330-FlashXe
Solvent: <unspecified>
Excitation: U pol 330±14nm with Xe-Lamp
Detection: U pol 490±14nm 900000 peak counts
grating 1200/500+
detector UV-red [PMT]

Fitted Parameters

Parameter	Value	Δ	δ
A_1[kCnts/Chnl]	0.86	±0.11	12%
τ_1[ns]	34 400 000	±2 300 000	6.5%
I_1[kCnts]	22.5	±1.3	5.5%
A_2[kCnts/Chnl]	2.292	±0.048	2.1%
τ_2[ns]	8 860 000	±590 000	6.7%
I_2[kCnts]	15.5	±1.8	11%
$Bkgr_{Dec}$[kCnts]	0.0311	±0.0021	6.7%
τ_{AvInt}[ns]	23 940 000	±840 000	3.5%

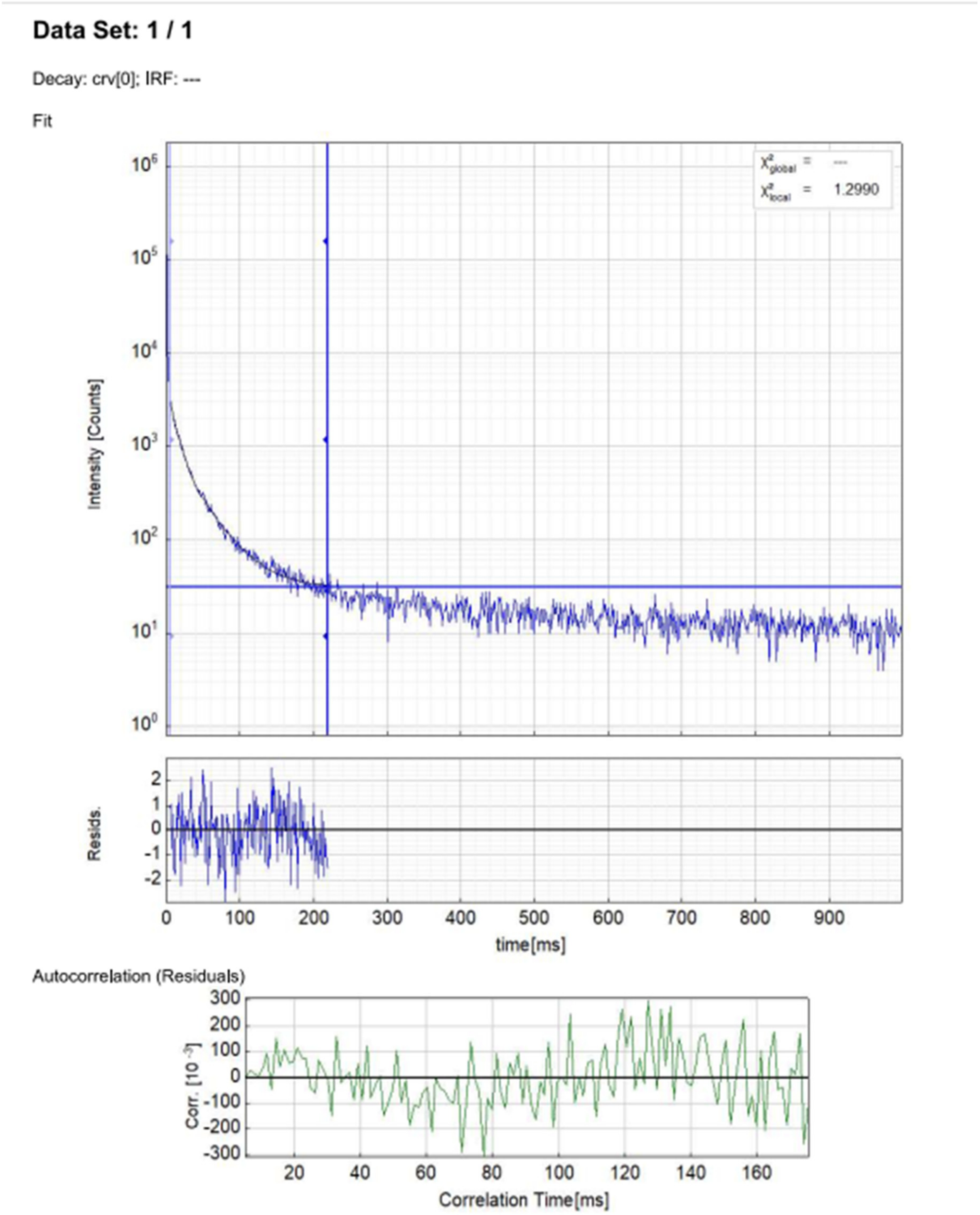

Figure S77. Fitting curve for **pAg3** at 77 K.

pAg4 (rt)

Container Curves

Container Properties

Measurement Context: Decay
Summary:
Sample: IBP-102-TV-RT-FLASH-ex345
Solvent: <unspecified>
Excitation: U pol 345±5nm with Xe-Lamp
Detection: U pol 495±5nm 90000 peak counts
grating 1200/500+
detector UV-red [PMT]

Fitted Parameters

Parameter	Value	Δ	δ
A_1[kCnts/Chnl]	1.179	±0.066	5.5%
τ_1[ns]	268 200	±5 400	2.0%
I_1[kCnts]	61.8	±2.2	3.4%
A_2[kCnts/Chnl]	3.067	±0.047	1.5%
τ_2[ns]	87 400	±2 500	2.9%
I_2[kCnts]	52.4	±2.2	4.1%
$Bkgr_{Dec}$[kCnts]	0.0091	±0.0008	8.1%
τ_{AvInt}[ns]	185 200	±1 400	0.8%

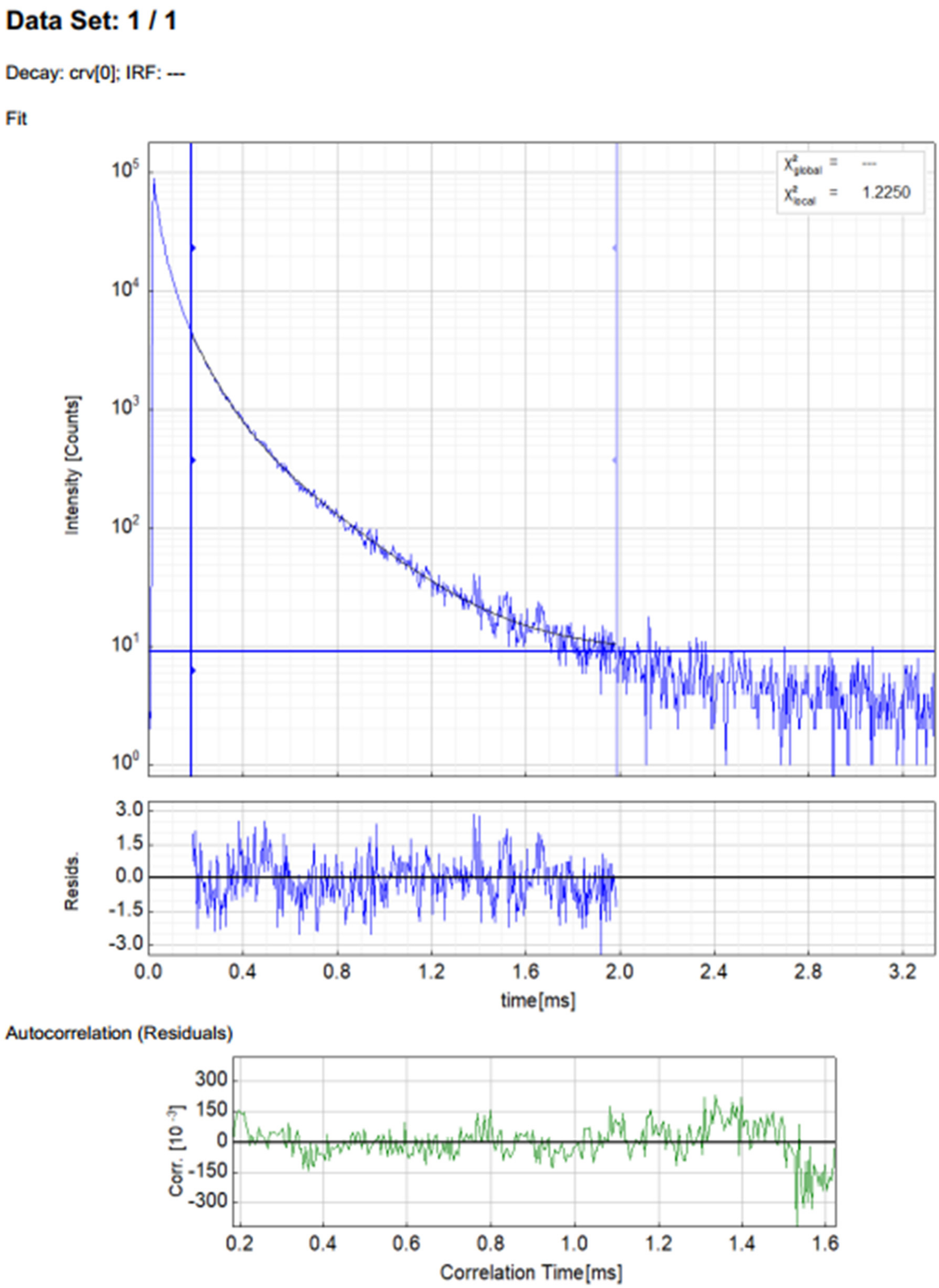

Figure S78. Fitting curve for **pAg4** at room temperature.

pAg4 (77K)

Container Properties

Measurement Context: Decay

Summary:

Sample:	IBP-102-TV-77K-flash-ex320
Solvent:	<unspecified>
Excitation:	U pol 320±14nm with Xe-Lamp
Detection:	U pol 470±14nm 90000 peak counts<br>grating 1200/500+<br>detector UV-red [PMT]

Fitted Parameters

Parameter	Value	Δ	δ
A_1[kCnts/Chnl]	0.0417	±0.0021	4.9%
τ_1[ns]	26 900 000	±2 000 000	7.2%
I_1[kCnts]	0.426	±0.019	4.4%
$Bkgr_{Dec}$[kCnts]	0.0022	±0.0002	8.2%
τ_{AvInt}[ns]	26 900 000	±2 000 000	7.2%

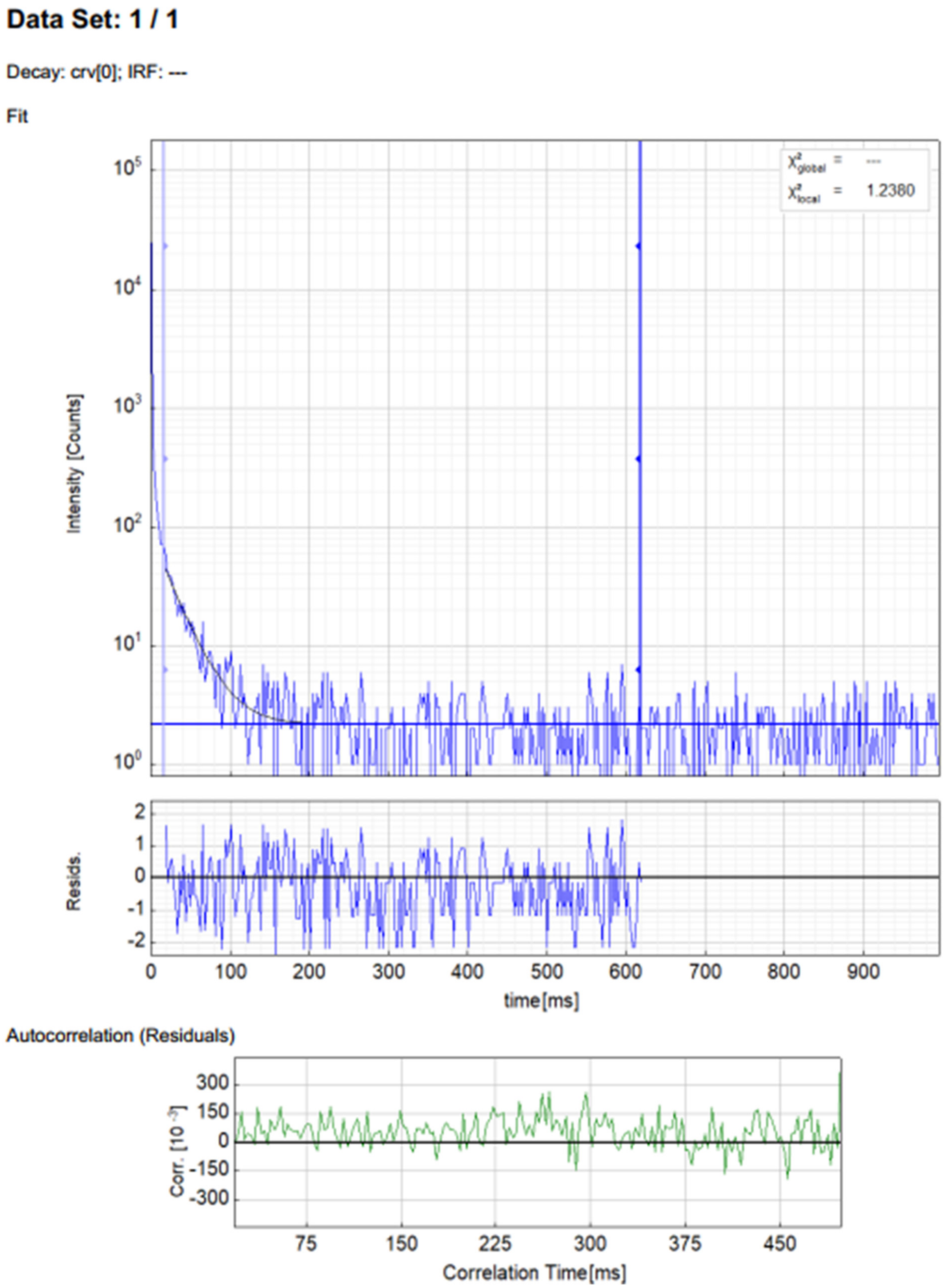

Figure S79. Fitting curve for **pAg4** at 77 K.

pAg5 (rt)

Container Properties

Measurement Context: Decay
Summary:

Sample:	Sample_16
Solvent:	<unspecified>
Excitation:	U pol 405±15nm with PLS-400
Detection:	U pol 468±12nm 500000 peak counts<br>grating 1200/500+<br>detector UV-red [PMT]

23/8/23, 8:24 EasyTau Report

Fitted Parameters

Parameter	Value	Δ	δ
A_1[kCnts/Chnl]	0.1287	±0.0094	7.3%
τ_1[ns]	143 700	±7 300	5.1%
I_1[kCnts]	7.22	±0.16	2.2%
A_2[kCnts/Chnl]	0.3370	±0.0046	1.4%
τ_2[ns]	28 200	±2 300	7.9%
I_2[kCnts]	3.71	±0.27	7.1%
$Bkgr_{Dec}$[kCnts]	0.0101	±0.0006	5.4%
τ_{AvInt}[ns]	104 500	±3 300	3.1%

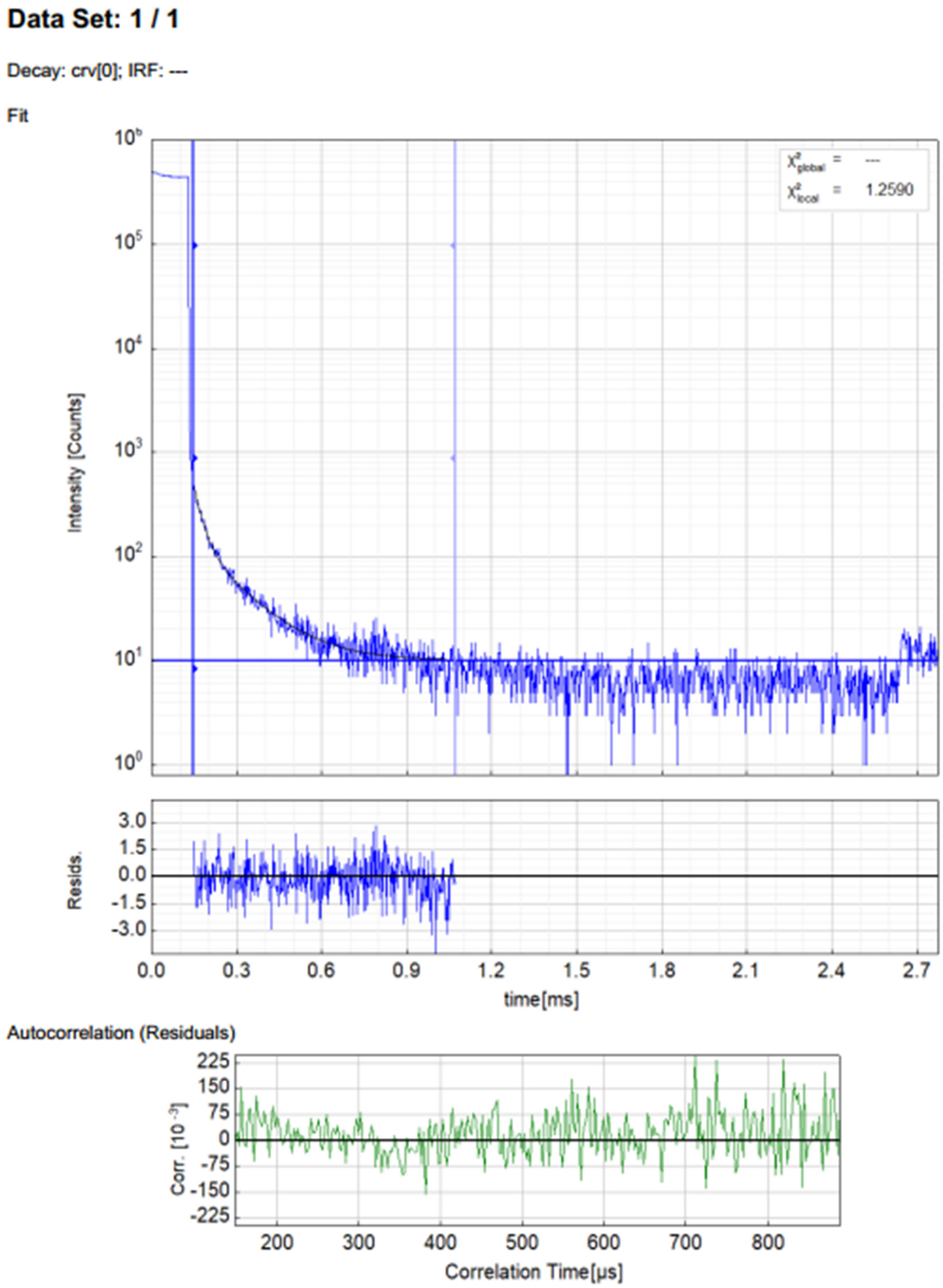

Figure S80. Fitting curve for **pAg5** at room temperature.

pAg5 (77K)

Container Properties

Measurement Context: Decay

Summary:

Sample:	Sample_19
Solvent:	<unspecified>
Excitation:	U pol 340±5nm with Xe-Lamp
Detection:	U pol 540±5nm 10000 peak counts<br>grating 1200/500+<br>detector UV-red [PMT]

23/8/23, 8:25 EasyTau Report

Fitted Parameters

Parameter	Value	Δ	δ
A_1[kCnts/Chnl]	0.175	±0.018	9.8%
τ_1[ns]	53 300 000	±3 200 000	5.9%
I_1[kCnts]	7.08	±0.45	6.3%
A_2[kCnts/Chnl]	0.726	±0.029	3.9%
τ_2[ns]	14 810 000	±710 000	4.7%
I_2[kCnts]	8.20	±0.52	6.3%
$Bkgr_{Dec}$[kCnts]	0.0110	±0.0006	4.8%
τ_{AvInt}[ns]	32 620 000	±390 000	1.2%

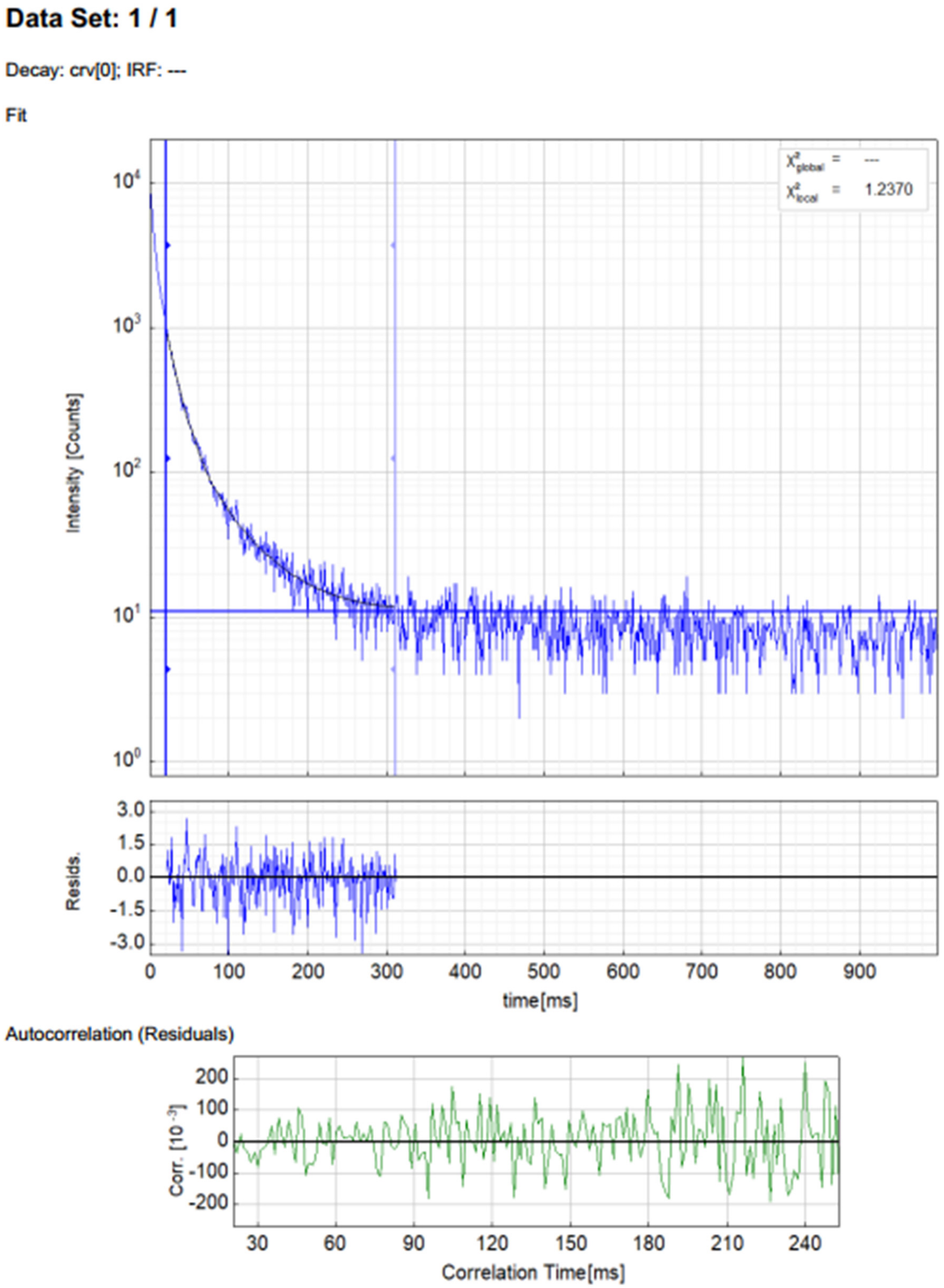

Figure S81. Fitting curve for **pAg5** at 77 K.

pAg6 (77K)

Container Properties

Measurement Context: Decay
Summary:
Sample: TV-IBP-133-Flash-exc340
Solvent: <unspecified>
Excitation: U pol 340±5nm with Xe-Lamp
Detection: U pol 540±5nm 5000 peak counts
grating 1200/500+
detector UV-red [PMT]

23/8/23, 8:39 EasyTau Report

Fitted Parameters

Parameter	Value	Δ	δ
A_1[kCnts/Chnl]	0.83	±0.11	13%
τ_1[ns]	15 140 000	±860 000	5.7%
I_1[kCnts]	38.1	±2.9	7.6%
A_2[kCnts/Chnl]	2.27	±0.12	4.9%
τ_2[ns]	5 530 000	±250 000	4.4%
I_2[kCnts]	38.2	±3.8	9.8%
$Bkgr_{Dec}$[kCnts]	0.0120	±0.0012	9.9%
τ_{AvInt}[ns]	10 330 000	±130 000	1.2%

Data Set: 1 / 1

Decay: crv[0]; IRF: ---

Fit

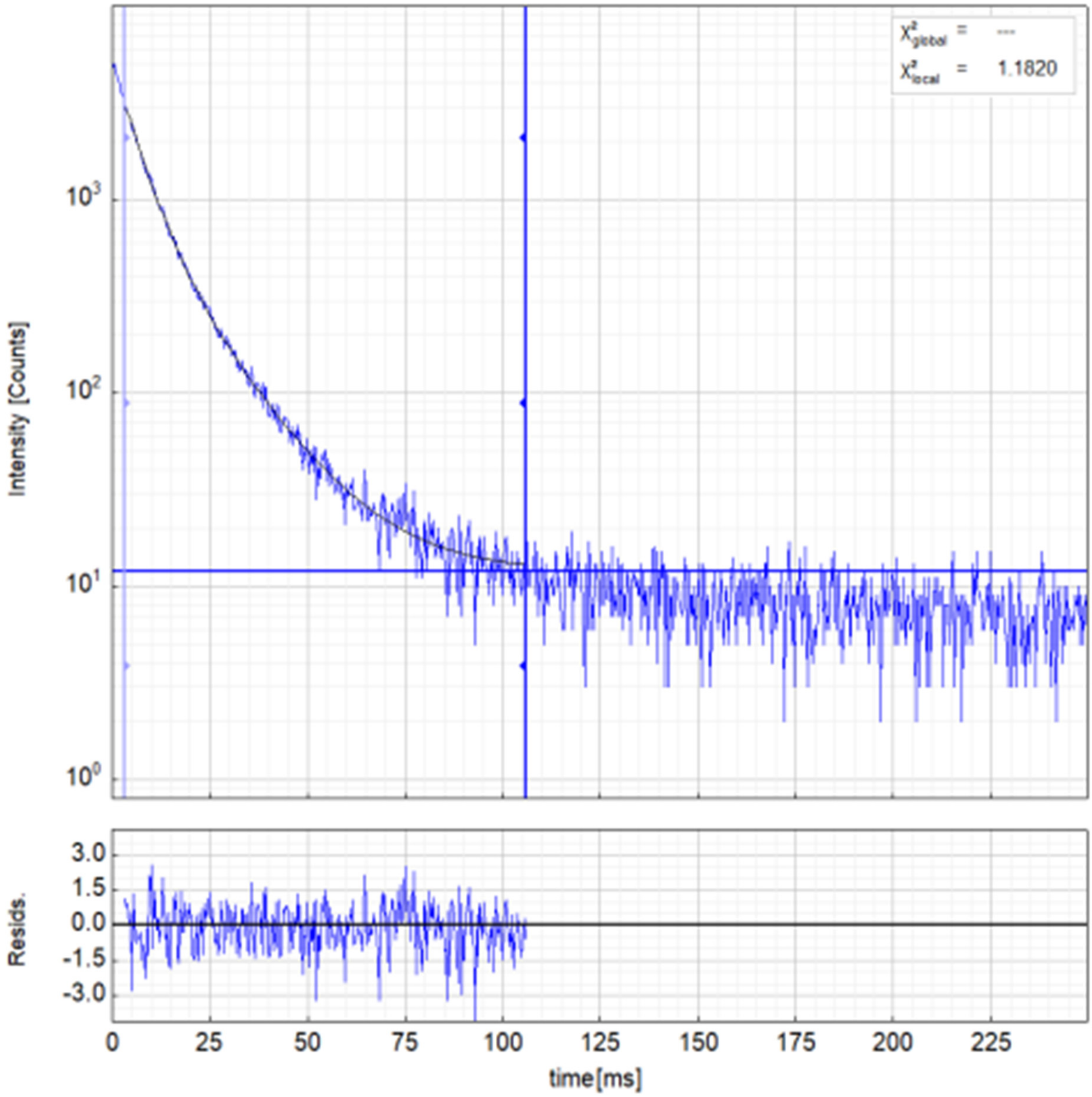

Autocorrelation (Residuals)

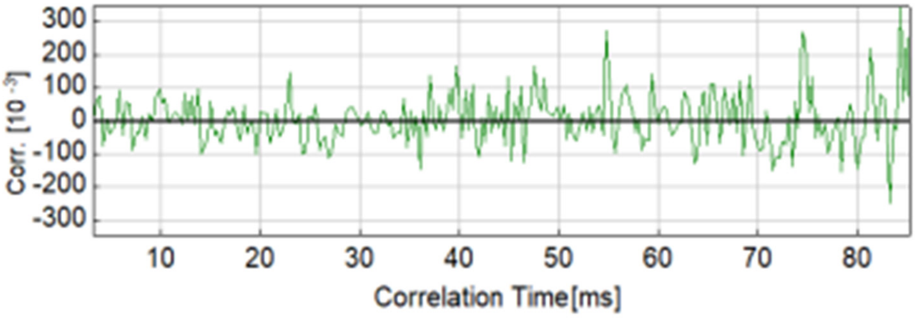

Figure S82. Fitting curve for **pAg6** at 77 K.

X-ray powder diffraction (XRD) spectra

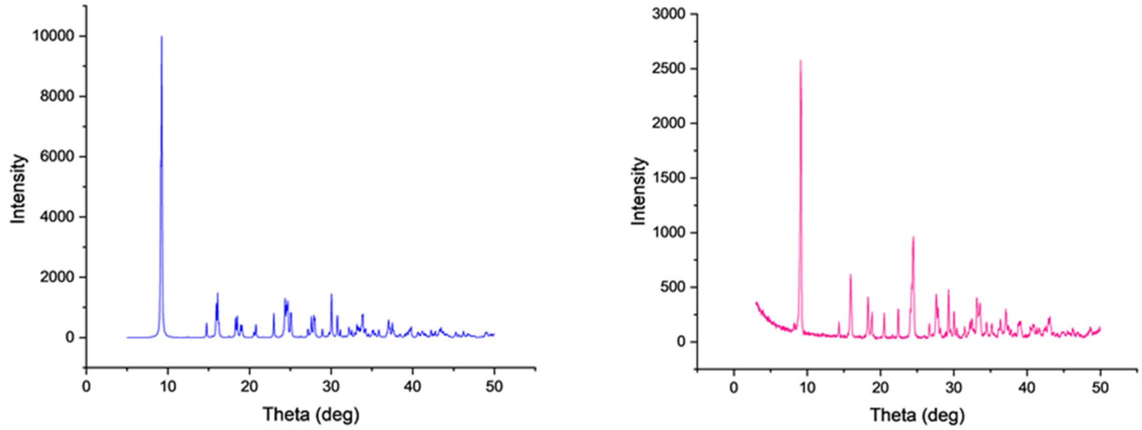

Figure S83. X-ray powder diffraction (XRD) spectra of **pAg1** (experimental, right; calculated from crystal X-Ray studies (left)

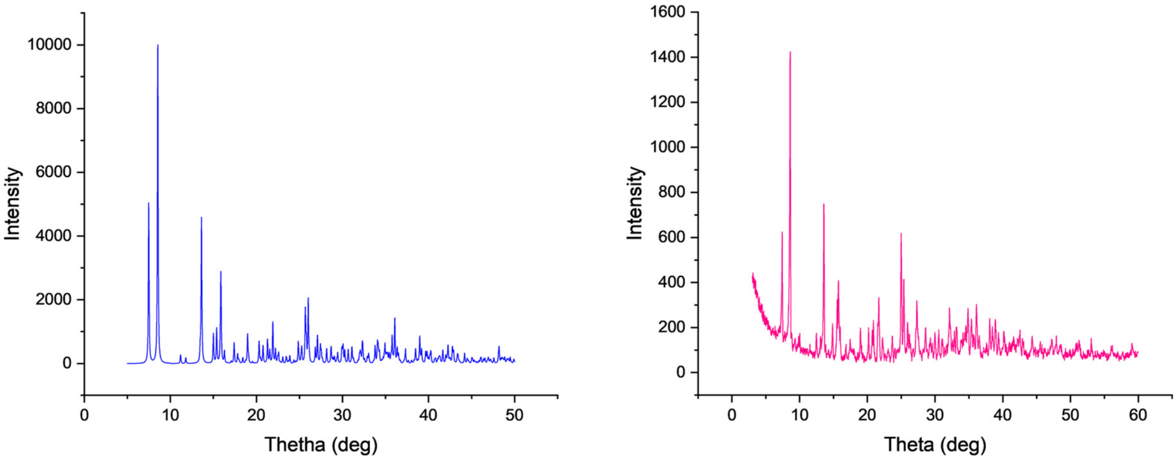

Figure S84. X-ray powder diffraction (XRD) spectra of **pAg2** (experimental, right; calculated from crystal X-Ray studies (left)

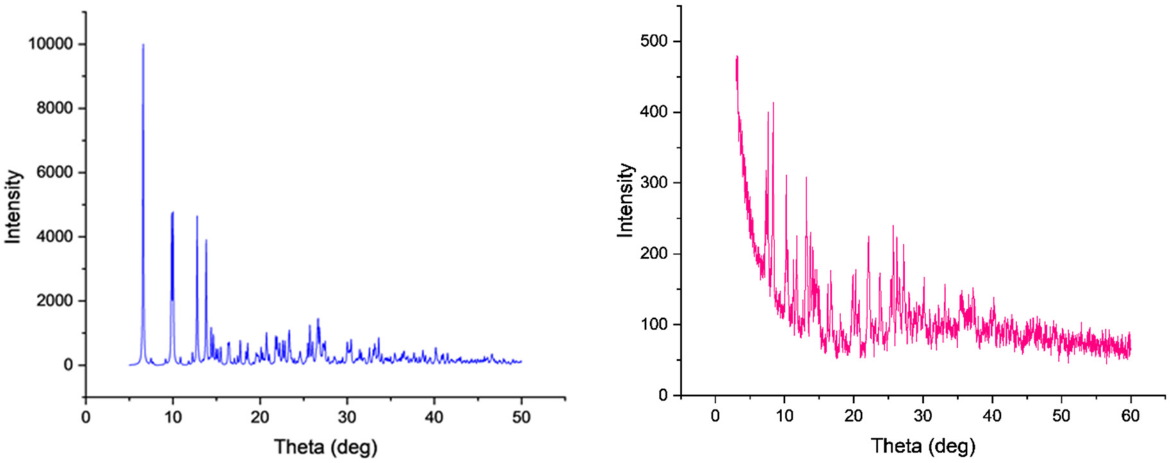

Figure S85. X-ray powder diffraction (XRD) spectra of **pAg4** (experimental, right; calculated from crystal X-Ray studies, left)

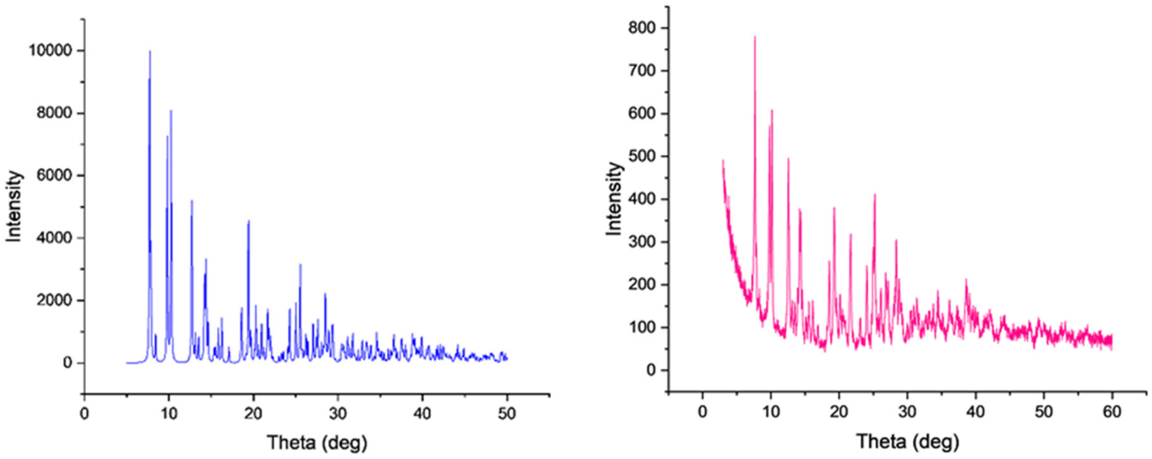

Figure S86. X-ray powder diffraction (XRD) spectra of **pAg6** (experimental, right; calculated from crystal X-Ray studies, left)

Crystal X-ray data

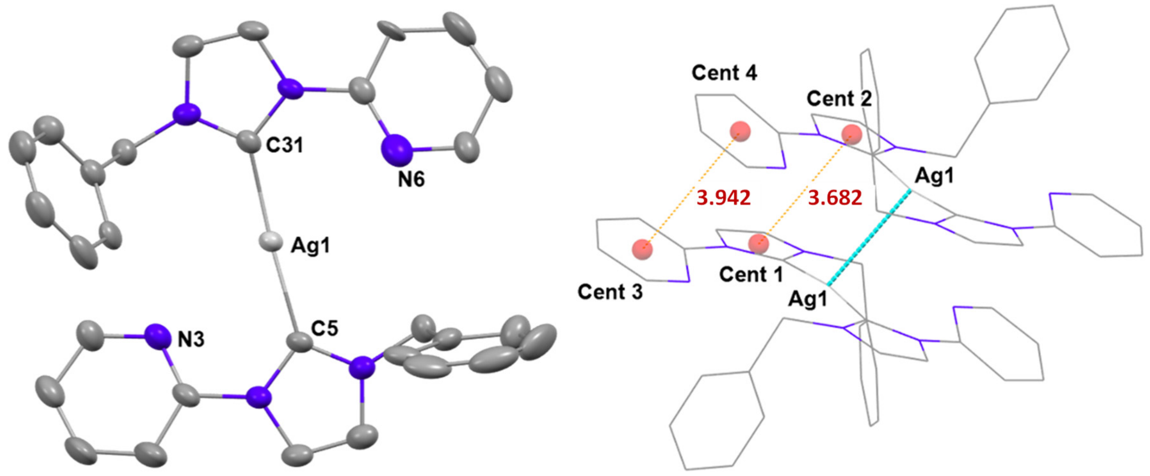

Figure S87. Diagrams of the cation of **Ag3** Hydrogen atoms have been omitted for clarity. Left: Ortep diagram showing the labeling scheme. Ellipsoids represent 50% probability level. Right: Diagram showing formation of dimers and distances between centroids of different rings.

The angle between the planes defined by the carbon atoms of the phenyl rings of the benzyl groups of different ligands is 66.06°.

Bond distances (Å): C5-Ag1 2.093(7), C31-Ag1 2.095(8), N6-Ag1 3.318(8), N3-Ag1 3.027(7), Ag1-Ag2 3.2950(11). Bond angles (°): C5-Ag1-C31 171.8(3).

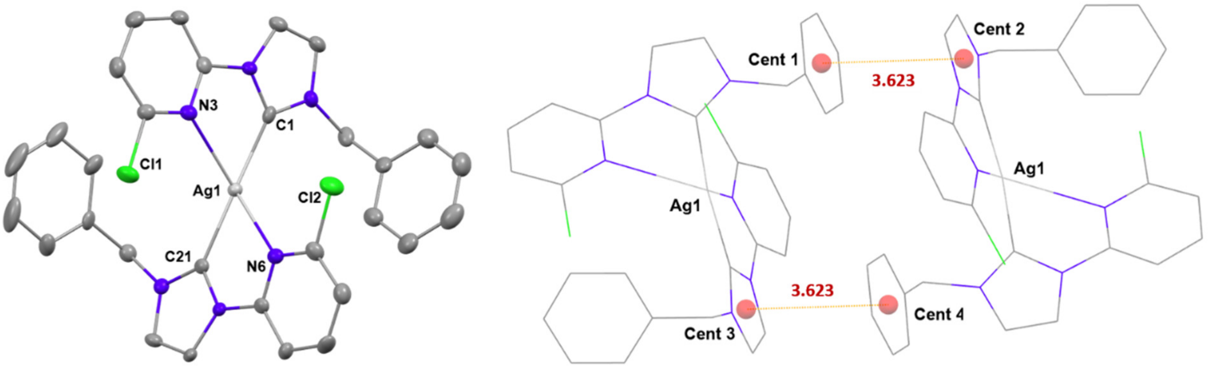

Figure S88. Diagrams of the cation of **Ag4**. Hydrogen atoms have been omitted for clarity. Left: Molecular diagram showing the atom labeling scheme. Right: Detail of distances between centroids of carbene and phenyl rings of different ligands.

The angle between planes defined by the carbon atoms of the phenyl rings of the benzyl groups of both ligands is 73.62°.

Bond distances (Å) and angles (°): C1-Ag1 2.092(10), C21-Ag1 2.095(9), N3-Ag1 2.691(8), N6-Ag1 2.716(7). Bond angles (°): C1 Ag1-C21 166.8(4), N3 Ag1-N6 102.8(3), C1-Ag1-N6 121.3(3), C21-Ag1-N3 120.8(3), N6-Ag1-C21 68.0(3), N3-Ag1 C1 68.4(3).

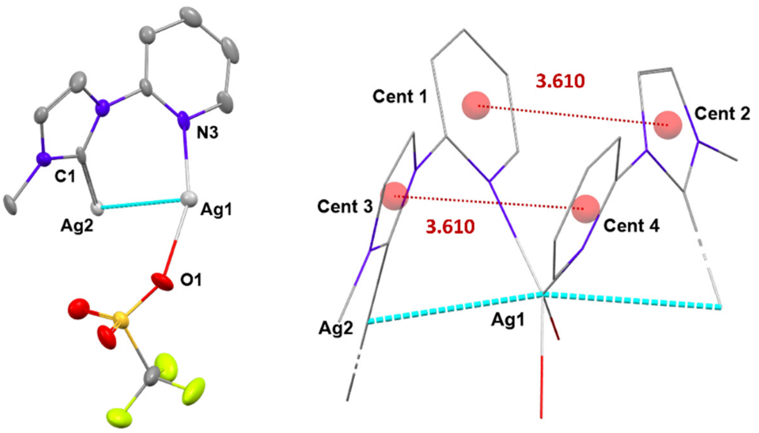

Figure S89. Diagrams corresponding to **pAg1**. Hydrogen atoms have been omitted for clarity. Left: Ortep diagram of the asymmetric unit of **pAg1**. Right: Distances between centroids of pyridine and carbene rings. To ensure clarity, the triflate anions are not included, except for the oxygen atoms that coordinate to silver. The angle between the planes originated by the pyridine and carbene rings of each ligand is 39.21°.
Bond distances (Å): Ag1-Ag2 3.1229(4), Ag1-O1 2.360(4), Ag2-N3 94.7(2), Ag2-N6 2.371(3), Ag2-C1 2.116(6). Bond angles (°): O1-Ag2-O1 101.30(10), N3-Ag1-N3 101.4(2), O1-Ag1-N3 86.71(17).

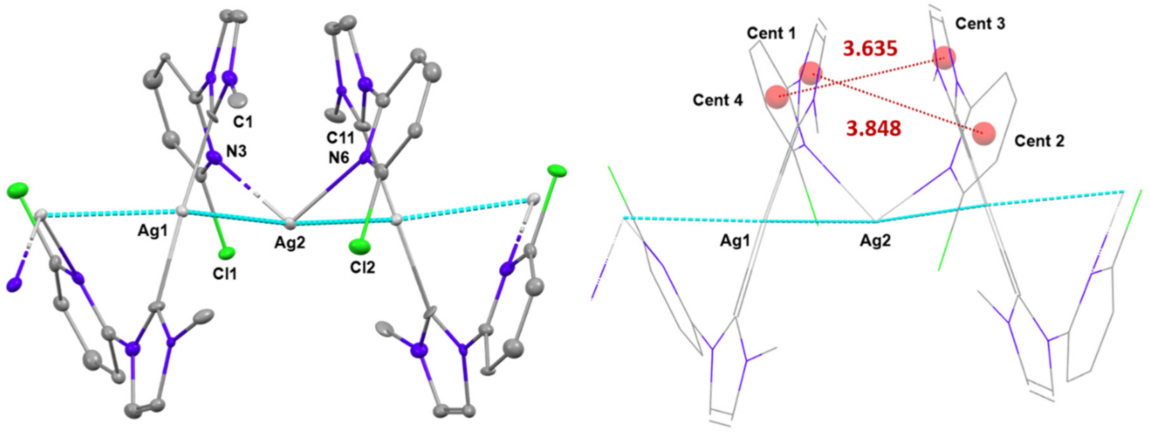

Figure S90. Diagrams of the cation of **pAg2**. hydrogen atoms have been omitted for clarity. Left: ORTEP diagram of the chain arrangement. Ellipsoids represent 50% probability. Right: Detail of distances between centroids of carbene and pyridine rings.
Bond distances (Å): Ag1-Ag2 2.91656(17), Ag1-Ag2 2.9799(17), C1-Ag1 2.086(15), C11-Ag1 2.083(16), N3-Ag2 2.368(14), N6-Ag2 2.317(13). Bond angles (°): N6-Ag2-N3 107.5(5), C11-Ag1-C1 175.3(6).

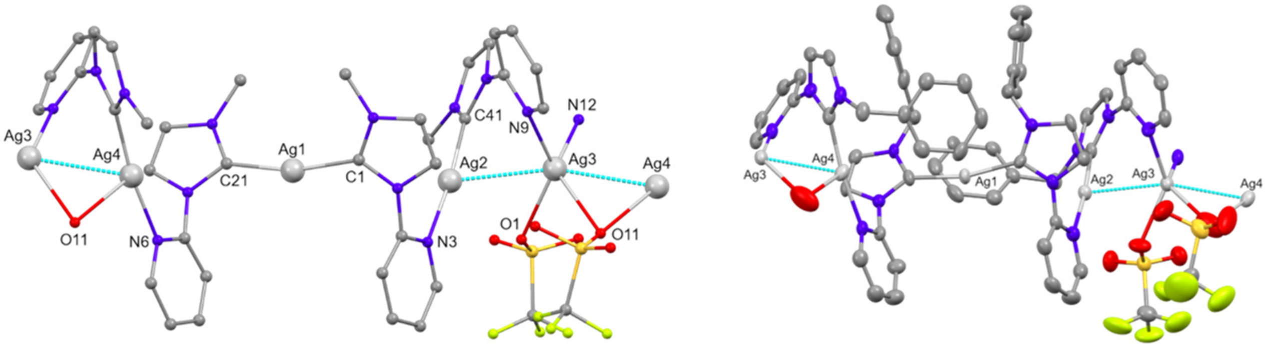

Figure S91. Diagrams of the cation of **pAg3**. Hydrogen atoms have been omitted for clarity. Left: Formation of chains. The phenyl rings of the benzyl groups, as well as the triflate anions non bonded to the silver atoms have been omitted for clarity. Right: molecular diagram displaying the atom labelling. Left: Ortep diagram. The distances between centroids of the phenyl rings of the benzyl groups of $\mathbf{L^3}$ ligands bonded to Ag(1) are longer than 4.5 Å and that between centroids of the pyridine fragments of $\mathbf{L^3}$ ligands bonded to Ag(1) is longer than 6 Å.

Bond distances (Å): Ag1-C21 2.086(6), Ag1-C1 2.087(6), Ag2-C41 2.081(7), Ag2-N3 2.145(6), Ag2-Ag3 2.9878(8), Ag3-N9 2.375(5), Ag3-O1 2.394(5), Ag3-N12 2.466(6), Ag3-Ag4 2.9465(8), Ag4-C61 2.074(7), Ag4-N6 2.141(6). Bond angles (°) C21-Ag1-C1 166.3(3), C41-Ag2-N3 174.5(2), C61-Ag4-N6 172.4(3), N9 Ag3 N12 102.72(19).

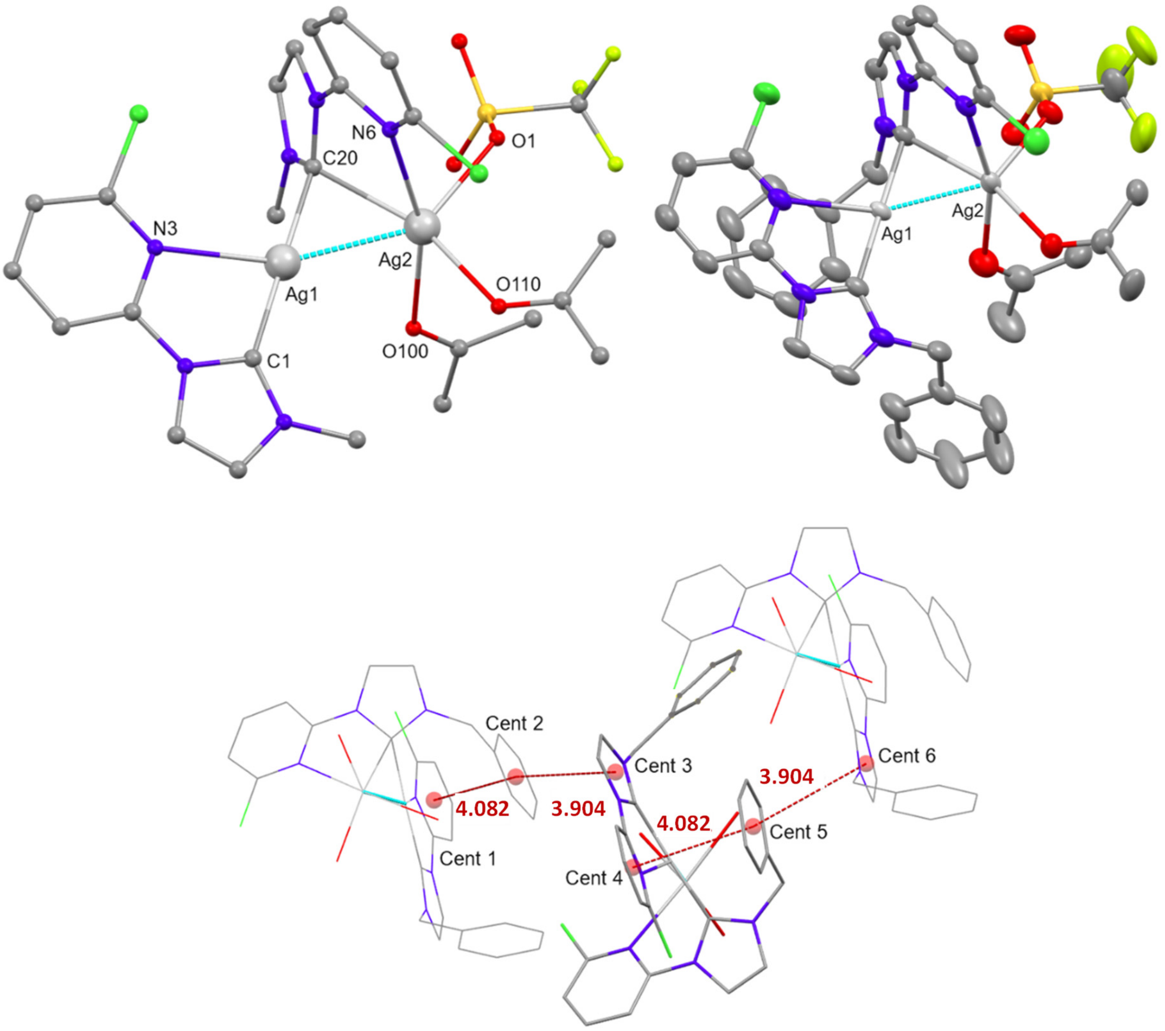

Figure S92. Diagrams of the cation of **pAg4**. Hydrogen atoms have been omitted for clarity. Up left: Simplified molecular diagram showing the labeling scheme in which the phenyl substituents of the benzyl groups have been omitted for clarity. Up right: Ortep diagram, ellipsoids represent 50% probability level. Hydrogen atoms have been omitted for clarity. Down: Detail of the distances between centroids of different rings belonging to different molecules.

Bond distances (Å): C1-Ag1 2.101(2), C20-Ag1 2.133(2), C20-Ag2 2.370(2), N3-Ag1 2.599(2), O100-Ag2 2.542(2), O110-Ag2 2.3399(16), O1-Ag2 2.4185(17), Ag1-Ag2 2.7353(3), Ag2-N6 2.6432(2).

Bond angles (°): C1-Ag1-C20 177.59(9), C1-Ag1-N3 70.71(8), C20-Ag1-N3 110.37(8), O110-Ag2-C20 157.11(7), O110-Ag2-O1 107.24(6), C20-Ag2-O1 94.48(7), O1-Ag2-O100 104.27(7), O110-Ag2-C20 157.11(6), O110-Ag2-O1 107.24(6), O110-Ag2-O100 83.08(7), C20-Ag2-O100 98.79(7), O1-Ag2-O100 104.27(6).

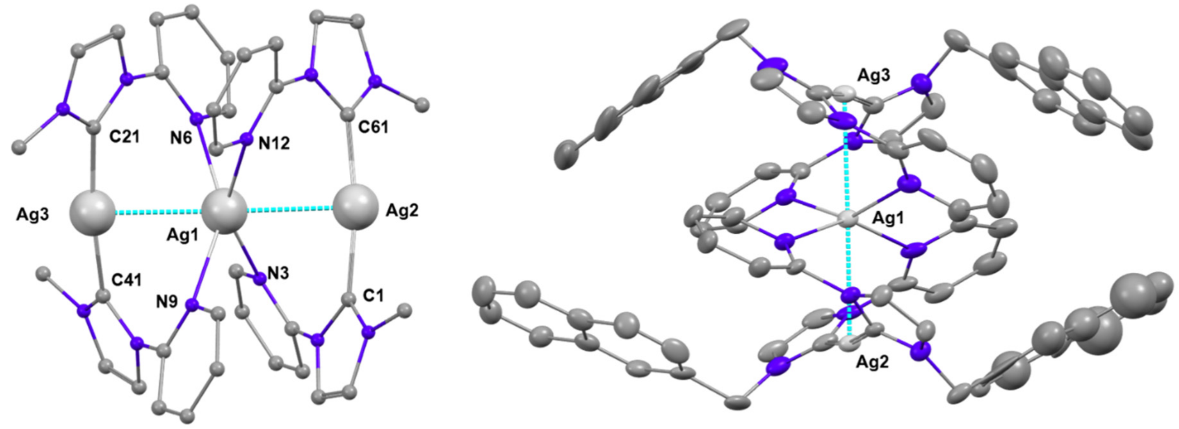

Figure S93. Diagrams of the cation of complex **pAg5**. Hydrogen atoms have been omitted for clarity. Left: Molecular diagram showing the labeling scheme. The naphtyl groups have been omitted for clarity. Right: Ortep diagram.

Intramolecular distances between centroids of nearly parallel carbene and pyridine rings range from 3.703 to 3.522 Å. Ligands are twisted, so that the plane defined by the naphthyl groups are twisted about 70° from the plane defined by the carbene fragment and this carbene unit is twisted about 40° from the plane defined by the pyridine ring.

Bond distances (Å): Ag1-Ag2: 2.9294(7), Ag1-Ag3 2.9370(7), C1-Ag(2) 2.070(7), C61-Ag(2) 2.077(7), N12-Ag1 2.432(6), N9-Ag1 2.467(6), N6-Ag1 2.466(6), N3-Ag1 2.411(6). Bond angles (°): N3-Ag1-N12 149.09(19), N3-Ag1-N6 104.1(2), N12-Ag1-N6 82.47(19), N3-Ag1-N9 84.1(2), N12-Ag1-N9 106.32(19), N6-Ag1-N9 148.31(19), C1-Ag2-C61 170.7(3), C21-Ag3-C41 172.5(3).

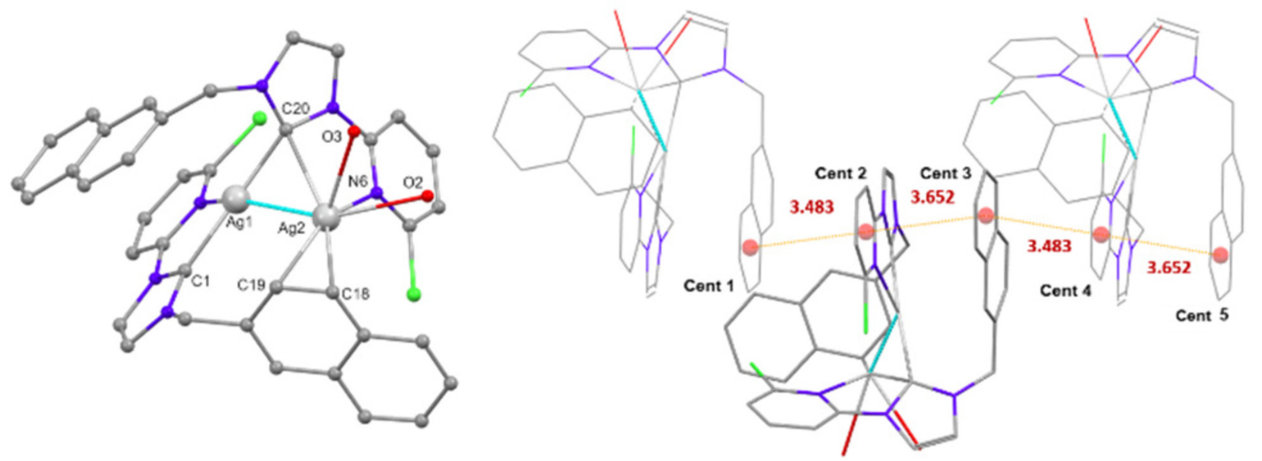

Figure S94. Diagrams of the cation of **pAg6**. Hydrogen atoms have been omitted for clarity. Up left: Simplified molecular diagram showing the labeling scheme. Up right: Ortep diagram, ellipsoids represent 50% probability level. Down: Detail of the distances between centroids of different rings belonging to different molecules.

Bond distances (Å): C1-Ag1 2.102(4), C18-Ag2 2.444(4), C19-Ag2 2.504(4) C20-Ag1 2.117(4), C20-Ag2 2.535(4), N3-Ag1 2.551(4), N6-Ag2 2.410 (4), Ag2-O2 2.694(4), Ag-O3 2.693(4), Ag1-Ag2 2.7232(4).

Bond angles (°): C1-Ag1-C20 170.84(17), C1-Ag1-N3 71.34(14), C20-Ag1-N3 117.42(14), N6-Ag2-C18 135.49(15), N6-g2-C19 151.39(14), C18-Ag2-C19 32.04(15), N6-Ag2-C20 69.03(15), C18-Ag2-C20 155.49(15), C19-Ag2-C20 127.42(15).

Table S1. Bond distances (Å) and angles (°) for complexes **Ag3**, **Ag4**, **pAg1**-**pAg6**.

Compound	Crystal data	
**Ag3**	**Bond distances**	C5-Ag1 2.093(7), C31-Ag1 2.095(8), N6-Ag1 3.318(8), N3-Ag1 3.027(7), Ag1-Ag2 3.2950(11).
	**Bond angles**	C5-Ag1-C31 171.8(3).
**Ag4**	**Bond distances**	C1-Ag1 2.092(10), C21-Ag1 2.095(9), N3-Ag1 2.691(8), N6-Ag1 2.716(7).
	**Bond angles**	C1 Ag1-C21 166.8(4), N3 Ag1-N6 102.8(3), C1-Ag1-N6 121.3(3), C21-Ag1-N3 120.8(3), N6-Ag1-C21 68.0(3), N3-Ag1 C1 68.4(3).
**pAg1**	**Bond distances**	Ag1-Ag2 3.1229(4), Ag1-O1 2.360(4), Ag2-N3 94.7(2), Ag2-N6 2.371(3), Ag2-C1 2.116(6).
	**Bond angles**	N3-Ag1-N3 101.4(2), O1-Ag1-N3 86.71(17).
**pAg2**	**Bond distances**	Ag1-Ag2 2.91656(17), Ag1-Ag2 2.9799(17), C1-Ag1 2.086(15), C11-Ag1 2.083(16), N3-Ag2 2.368(14), N6-Ag2 2.317(13).
	**Bond angles**	N6-Ag2-N3 107.5(5), C11-Ag1-C1 175.3(6).
**pAg3**	**Bond distances**	Ag1-C21 2.086(6), Ag1-C1 2.087(6), Ag2-C41 2.081(7), Ag2-N3 2.145(6), Ag2···Ag3 2.9878(8), Ag3-N9 2.375(5), Ag3-O1 2.394(5), Ag3-N12 2.466(6), Ag3-Ag4 2.9465(8), Ag4-C61 2.074(7), Ag4-N6 2.141(6).
	**Bond angles**	C21-Ag1-C1 166.3(3), C41-Ag2-N3 174.5(2), C61-Ag4-N6 172.4(3), N9-Ag3-N12 102.72(19).
**pAg4**	**Bond distances**	C1-Ag1 2.101(2), C20-Ag1 2.133(2), C20-Ag2 2.370(2), N3-Ag1 2.599(2), O100-Ag2 2.542(2), O110-Ag2 2.3399(16), O1-Ag2 2.4185(17), Ag1-Ag2 2.7353(3), Ag2-N6 2.6432(2).
	**Bond angles**	C1-Ag1-C20 177.59(9), C1-Ag1-N3 70.71(8), C20-Ag1-N3 110.37(8), O110-Ag2-C20 157.11(7), O110-Ag2-O1 107.24(6), C20-Ag2-O1 94.48(7), O1-Ag2-O100 104.27(7), O110-Ag2-C20 157.11(6), O110-Ag2-O1 107.24(6), O110-Ag2-O100 83.08(7), C20-Ag2-O100 98.79(7), O1-Ag2-O100 104.27(6).
**pAg5**	**Bond distances**	Ag1-Ag2 2.9294(7), Ag1-Ag3 2.9370(7), C1-Ag(2) 2.070(7), C61-Ag(2) 2.077(7), N12-Ag1 2.432(6), N9-Ag1 2.467(6), N6-Ag1 2.466(6), N3-Ag1 2.411(6).
	**Bond angles**	N3-Ag1-N12 149.09(19), N3-Ag1-N6 104.1(2), N12-Ag1-N6 82.47(19), N3-Ag1-N9 84.1(2), N12-Ag1-N9 106.32(19), N6-Ag1-N9 148.31(19), C1-Ag2-C61 170.7(3), C21-Ag3-C41 172.5(3).
**pAg6**	**Bond distances**	C1-Ag1 2.102(4), C18-Ag2 2.444(4), C19-Ag2 2.504(4) C20-Ag1 2.117(4), C20-Ag2 2.535(4), N3-Ag1 2.551(4), N6-Ag2 2.410(4), Ag2-O2 2.694(4), Ag-O3 2.693(4), Ag1-Ag2 2.7232(4).
	**Bond angles**	C1-Ag1-C20 170.84(17), C1-Ag1-N3 71.34(14), C20-Ag1-N3 117.42(14), N6-Ag2-C18 135.48(15), N6-Ag2-C19 151.39(14), C18-Ag2-C19 32.04(15), N6-Ag2-C20 69.03(15), C18-Ag2-C20 155.49.(15), C19-Ag2-C20 127.42(15).
